# Supplementary material for: Improving access to health services through health reform in Lesotho: Progress made towards achieving Universal Health Coverage
Source: PLOS Glob Public Health. 2022 Nov 16;2(11):e0000985. doi: 10.1371/journal.pgph.0000985 (PMC10021396; doi:10.1371/journal.pgph.0000985)
Supplement: S1 Text — (PDF) [file pgph.0000985.s002.pdf]

Bo-mphato
Litšebeletsong
Tsa Bophelo
Partners In Health

NATIONAL HEALTH REFORM IN LESOTHO: REFORMING THE PRIMARY HEALTHCARE SYSTEM

PARTNERS IN HEALTH
NOVEMBER 2019

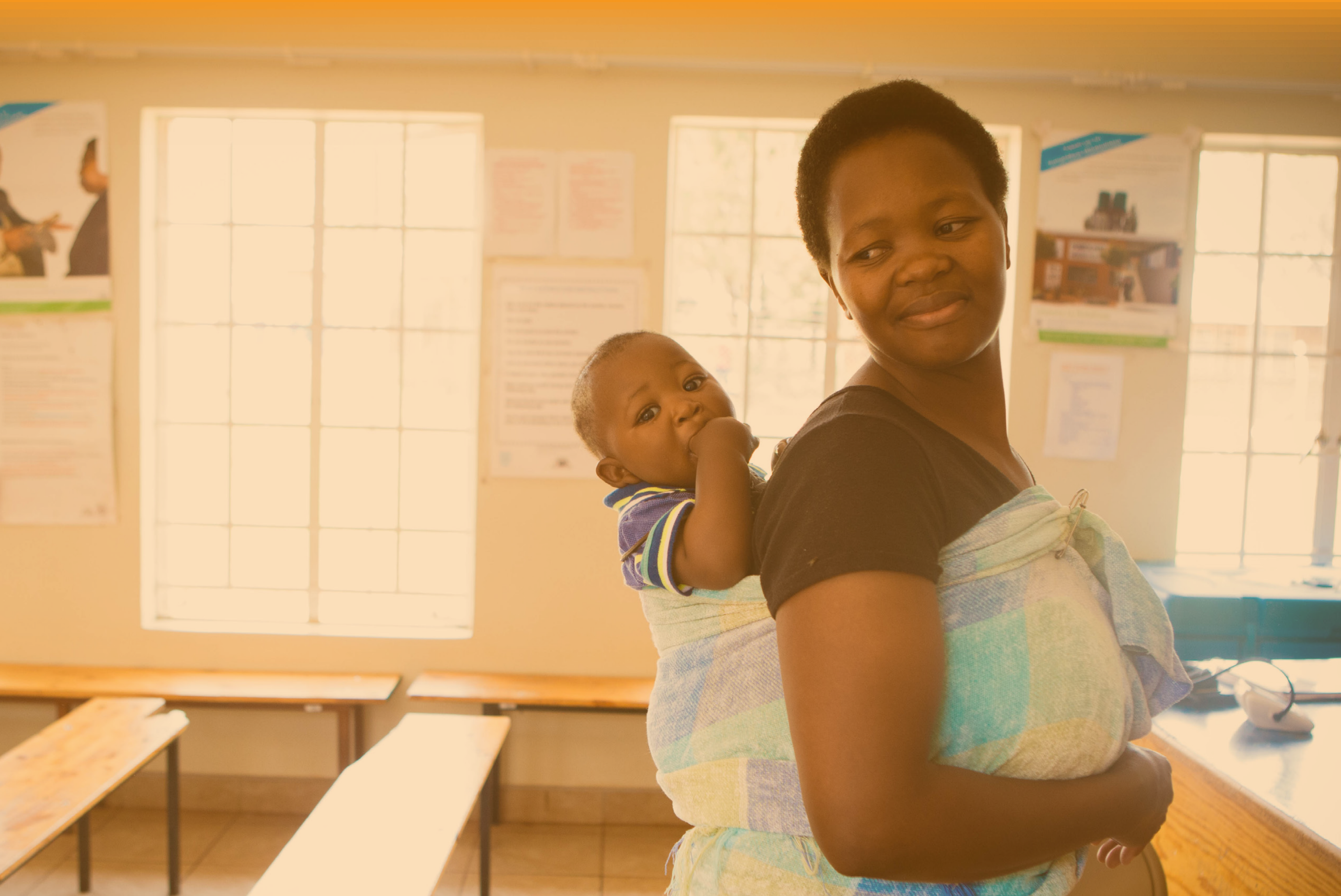

Malieketso Ntlele, 35, carries her 7-month-old son, Tlala Ntlele, during an early childhood checkup at Pontmain Health Center in Leribe District, Lesotho, in 2018. Ntlele stayed at Pontmain's maternal waiting home before giving birth, ensuring she was close to doctors when labor began. The family lives in the village of Ha Makhalhane, about 9 miles from the health center. (Photo by Cecille Joan Avila / Partners In Health)

TABLE OF CONTENTS

Executive Summary 4

Introduction 6

Methodology 7

Background of the National Health Reform....8

Results: Progress Toward UHC Targets 12

National Health Reform Interventions....25

1. Service Delivery 27
2. Community Engagement....33
3. Health Workforce 38
4. Medical Products and Technologies 39
5. Financing....41
6. Systems for Data Collection and Management 43
7. Leadership and Governance....44

Recommendations 47

Conclusion....52

LIST OF FIGURES

Figure 1: Methodology....8

Figure 2: Lesotho's Health Care Delivery System 9

Figure 3: National Health Reform Strategies and Levels of Implementation 10

Figure 4: Number of Health Facilities in the Reform Districts 11

Figure 5: Core Strategies and Interventions of the National Health Reform....11

Figure 6: Comparison of Quarterly Averages for Key Indicators Before and After Implementation of the National Health Reform 12

Figure 7: Number of Women Attending First ANC Visit....13

Figure 8: First ANC Visits by District 14

Figure 9: Number of Women Attending Fourth ANC Visit....15

Figure 10: Completion of Fourth ANC Visit by District....15

Figure 11: Number of Women Attending ANC Visits 16

Figure 12: First ANC Visits at Hospitals and Health Centers 17

Figure 13: Fourth ANC Visits at Hospitals and Health Centers 17
Figure 14: Number of Facility-Based Deliveries By District 18
Figure 15: Number of Women Delivering in a Health Center....18
Figure 16: Facility-Based Delivery and Maternity Mortality 19
Figure 17: Number of Women Attending First PNC Visit....20
Figure 18: PNC Visits at the Hospital and Health Centers....20
Figure 19: Children Fully Immunized at Year 1 at Health Centers....21
Figure 20: Children Fully Immunized at Year 1 by District 22
Figure 21: Number of HIV Tests Performed at the Health Center Level 23
Figure 22: Number of Positive HIV Tests and New ART Enrollments....23
Figure 23: HIV Viral Load Suppression by District....24
Figure 24: Health Systems Building Blocks, Adapted from the WHO Health Systems Framework 25
Figure 25: Comparison of Key Indicators Before and After Implementation of the National Health Reform 26
Figure 26: Facility-Based Deliveries at Hospitals and Health Centers....27
Figure 27: Average Distance from Home to Health Center for Expectant Mothers in Maternal Waiting Homes....29
Figure 28: Percent of Pregnant Women Admitted to Maternal Waiting Homes....30
Figure 29: Number of Pregnant Women Admitted to Maternal Waiting Homes 30
Figure 30: Number of Clients Active in the NCD Program in Berea District (January 2016-June 2019) ...31
Figure 31: Emergency Referrals During Pregnancy and Labor from Health Center to Hospital 32
Figure 32: Summary of Guidelines for TB/HIV and MMRPA VHWs....34
Figure 33: Maternal Accompaniments Compared to ANC and PNC Visits....35
Figure 34: Maternal Accompaniments and ANC/PNC Visits 36
Figure 35: VHW Meetings Per Quarter....37
Figure 36: Staffing Levels at Health Centers Before and After Implementation of the National Health Reform....38
Figure 37: Number of VHWs Pre and Post National Health Reform 39
Figure 38: National Health Reform Spending Per Person....42

ACRONYMS

**ANC**	Antenatal care
**ART**	Antiretroviral therapy
**ARV**	Antiretroviral drugs
**CHAL**	Christian Health Association of Lesotho
**CHIS**	Community Health Information System
**DHMT**	District Health Management Team
**EPHS**	Essential Package of Health Services
**HIV**	Human Immunodeficiency Virus
**GoL**	Government of Lesotho
**KPIs**	Key Performance Indicators
**LTFU**	Lost to follow-up
**MAF**	Mission Aviation Fellowship
**MMRP**	Maternal Mortality Reduction Program Assistants
**MoH**	Lesotho Ministry of Health
**NCD**	Non-communicable disease
**PHC**	Primary Health Care
**PIH**	Partners In Health
**PIH-L**	Partners In Health Lesotho
**PNC**	Postnatal Care
**RI**	Rural Health Initiative
**SDGs**	Sustainable Development Goals
**TB**	Tuberculosis
**UHC**	Universal Health Coverage
**VHW**	Village Health Worker
**WHO**	World Health Organization

EXECUTIVE SUMMARY

Introduction

At the invitation of the Lesotho Ministry of Health in 2006, Partners In Health commenced work in Lesotho by launching the Rural Health Initiative to support seven rural health clinics in the most inaccessible mountainous areas of the country. Partners In Health Lesotho's approach at the seven rural clinics delivered better outcomes at lower costs than other public facilities in the country. The Government of Lesotho was impressed by these results and asked Partners In Health Lesotho to serve as its technical advisor for reforming the primary health care system. The first phase of the National Health Reform was initiated in April 2014, with implementation in four districts: Berea, Leribe, Butha-Buthe and Mohale's Hoek. In 2018, the Ministry of Health in collaboration with Partners In Health commissioned an evaluation of the first phase of the National Health Reform, which showed statistically significant increases in service utilization indicators for maternal and child health. Building on findings of the National Health Reform evaluation, this report presents an in-depth analysis of the results, including key policy recommendations.

Methodology

This report used analyzed outputs as well as qualitative and quantitative data collected by the National Health Reform Evaluation. Data on quantitative performance indicators were collected from 68 health facilities for a five-year period between July 2012 and July 2017. Qualitative data was collected from 30 key informant interviews, one-on-one discussions with health center staff and district teams, and observations from site visits.

National Health Reform

The Government of Lesotho launched the National Health Reform to decentralize and improve the delivery of primary health care services and ultimately put the country on the path to achieve Universal Health Coverage. The National Health Reform promoted a system-wide improvement of district-led primary health care employing three core strategies: improving service delivery, strengthening health system management, and institutionalizing a professionalized Village Health Worker program.

Results

There was an upward trend in selected maternal health, child health, and HIV testing and treatment indicators during the implementation of the National Health Reform (April 2014 to July 2017) compared to the baseline (July 2012 to March 2014). These upward trends in utilization of key health services will contribute towards much needed change required to improve high maternal and child mortality and HIV prevalence in Lesotho. Overall, comparison of quarterly averages between the baseline and implementation periods showed:

- 85% increase in fourth ANC visits;
- 15-fold increase in Facility-Based Deliveries at the health center level;
- nearly a threefold increase in the number of children under one who are fully immunized;
- nearly a threefold increase in HIV testing; and
- double the number of new ART enrollments.

The implementation of the National Health Reform also created a shift in patient flow from hospitals to health centers, indicating better service delivery at the health center level. Improvements in health service utilization reflect the strong investments on health systems and building of management capacity at the district level to drive these results. With these improvements, the National Health Reform set the primary health care system of Lesotho on a path towards achieving Universal Health Coverage in the country.

Recommendations

Further analysis of data from the National Health Reform evaluation has highlighted several learnings which demand effective action from the Ministry of Health and partners in order to sustain the gains of the National Health Reform and accelerate Lesotho's progress towards Universal Health Coverage. The below policy recommendations are outlined for consideration by the Government of Lesotho and the Ministry of Health.

1. Empower District Health Medical Teams to manage financial and human resources
2. Build management and leadership capacity in primary health care
3. Enhance accountability using management standards and community scorecard
4. Use primary health care key performance indicators for performance management
5. Develop guidance and standards on mentoring and supervision
6. Harmonize incentive mechanisms
7. Strengthen community program though formalizing Village Health Workers and establishing community health information systems
8. Update establishment list, optimize human resources allocations based on demand
9. Update the Essential Package of Health Services (EPHS) to be aligned with Universal Health Coverage
10. Strengthen leadership at the Ministry of Health level to sustain and scale the National Health Reform through coordination and alignment

Conclusion

The National Health Reform demonstrated that relatively modest investments targeted at improving comprehensive primary health care service delivery are beneficial in the short term for addressing current priorities and in the long term for preparing the health system to address emerging priorities. It also showed that, heavily funded vertical programs, including those addressing HIV, benefited from the improvement of the primary health care platform to provide integrated health care. Promoting local decision-making by empowering districts to lead primary health care and supporting all of the building blocks of the health system address inequalities in access to health care and makes primary health care an effective tool for advancing Universal Health Coverage.

INTRODUCTION

The mission of Partners In Health (PIH) is to provide a preferential health care option for the poor by striving to achieve two overarching goals: 1) to bring the benefits of modern medical science to those most in need of them and 2) to serve as an antidote to despair. PIH has stayed true to its mission in the Kingdom of Lesotho by working in partnership with the Ministry of Health (MoH) to provide access to healthcare for vulnerable populations not adequately served by the country's health system.

Lesotho is a low-middle income country with a population of over 2 million, and is physically enclaved by the much larger Republic of South Africa. About 21% of Lesotho's population resides in the mountains, around a quarter of whom live in very inaccessible areas with limited or no access to roads.[1] Undeterred by the physical and logistical challenges of reaching such populations, in 2006 PIH and the MoH identified seven remote health centers in four districts and launched the Rural Health Initiative (RI). The initiative began as an effort to support HIV care in health facilities in the most remote areas of the country that were only reachable by small planes, on horseback, or on foot.[2]

PIH faced a very high burden of communicable and non-communicable diseases (NCDs) in Lesotho. Lesotho has the second highest HIV prevalence (25.6%) and TB incidence (665 per 100,000) and has among the highest rates of maternal mortality (1,024 deaths per 100,000 live births) and infant mortality (59 deaths per 1,000 live births) in the world.[3,4,5] The population served by the Rural Health Initiative is no exception to these burdens. Populations in these remote areas often have lower socioeconomic status than those living in more urban areas; the rural poverty rate in Lesotho is 61% as compared to urban poverty rates of 39%.[6]

Since its inception, the Rural Health Initiative has transitioned from disease-specific programming to a comprehensive primary health care approach that would strengthen multiple components of the primary health care system including engagement at the community-level, improving quality of services, and improvements to the health care infrastructure such as supply chain and information systems. This approach has led to substantial results in improving access and utilization of quality primary health care services including HIV treatment, demonstrating a practical approach for achieving Universal Health Coverage (UHC). Despite achievements in facilities supported by the Rural Health Initiative, the overall situation of national primary health care delivery through 175 health centers, was dismal.

In 2013, the Government of Lesotho (GoL) commissioned an assessment of the national health system, conducted by a team composed of PIH-Lesotho (PIH-L) staff, the MoH, and a group of external consultants. The report observed that there was collective failure of primary health care due to inadequately resourced health facilities and a lack of managerial capacity and resources at the district level. Health facilities were not providing consistent or quality services, resulting in very low patient attendance and low case-finding rates for HIV, TB, and other diseases. Additionally, very few health centers were able to adequately provide facility-based deliveries for pregnant women. The assessment concluded that large-scale reform of the existing health sector model was necessary. The GoL was impressed by PIH-L's results at RI facilities, including better outcomes at lower costs than other public facilities in the country, and asked PIH-L to serve as its technical advisor to reform the primary health care system.

From its experience with the RI, PIH recognized that a comprehensive approach which supports how a primary health care system is resourced, operated, and managed is crucial to meaningfully improve the performance of the health system across multiple service delivery areas. PIH supported the MoH to initiate

---

[1]Bureau of Statistics (BOS), 2014
[2]Planes are operated by Mission Aviation Fellowship (MAF is a Christian organization that provides aviation services to villagers in the world's most remote areas
[3]LePHIA Population-Based HIV Impact Assessment 2016-2017
[4]Global Tuberculosis Report (2018) World Health Organization
[5]Lesotho Demographic Health Survey (2014)
[6]World Bank, 2015

a national reform of the primary health care system, known as the National Health Reform. The first phase of the National Health Reform was initiated in 2014, with implementation in four districts: Berea, Leribe, Butha-Buthe and Mohale's Hoek. The National Health Reform employed a comprehensive approach to improving primary health care by initiating reform ideas across all health system building blocks.

In 2018, after three years of implementation, the MoH in collaboration with PIH commissioned an evaluation of the first phase of the National Health Reform, which showed statistically significant increases in service utilization indicators for maternal and child health.[7] The evaluation further demonstrated that heavily funded vertical programs, including those addressing HIV, benefited from the improvement of the primary health care platform to provide integrated health care. Building on the evaluation findings, this report will present an in-depth analysis of the results of the National Health Reform including; comparison of health service utilization results with UHC targets, analysis of interventions using health system building blocks framework, perspectives of health workers at all levels on the national health reform and key policy recommendations for the way forward.

METHODOLOGY

This report used analyzed outputs as well as qualitative and quantitative data collected by the National Health Reform Evaluation. Quantitative performance indicators were collected at 68 health facilities and analyzed through an interrupted time-series design to examine trends before and during the National Health Reform and to determine whether changes where attributable to the Reform. Data was collected in key health and health-related areas including: maternal health, child health, HIV, TB, human resources, supervision, medicines and medical supplies, and information systems for the five-year period between July 2012 and July 2017.

The evaluation also collected qualitative data through key informant interviews with 30 respondents from the community, health center, district, and national levels. In addition to qualitative data collected by the evaluation, data from site visit observations and one-on-one discussions with health center staff and district teams were utilized.

This report employed the following approaches for in-depth analysis.

- Measure progress toward UHC targets for Maternal and Child Health, and HIV, disaggregated by district.
- Analysis of pre-intervention and post-intervention trends over time for selected indicators.
- Correlation of multiple indicators to understand relationships between interventions and outcomes and impact of one intervention on another.
- Significant change stories narrated by interviewees working in facilities in the National Health Reform districts.
- World Health Organization's (WHO) health systems building blocks employed as a framework to analyze the effect of interventions on the health system.[8]
- Review of experiences of other countries to produce recommendations for the way forward.

---

[7] Ministry of Health Lesotho, Partners In Health , Lesotho National Health Reform Evaluation Report, (October 2018)
[8] World Health Organization. Monitoring the building blocks of health systems: A handbook of indicators and their measurement strategies. Geneva: WHO 2010

Figure 1: Methodology

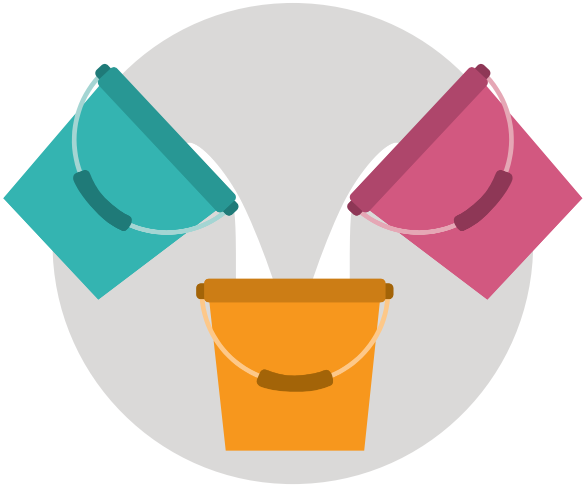

Quantitative Data

- Data collected from 70 health facilities between July 2012 and July 2017
- Data from 68 health facilities included in the evaluation

Qualitative Data

- Key informant interviews conducted with 30 respondents at the community, health center, hospital, district, and national levels
- One on one interviews with health center and hospital staff and district health management teams (DHMTs)

Analysis

- Measure progress toward Universal Health Coverage (UHC)
- Analysis of key indicators before, during, and after implementation of the National Health Reform
- Collection of change stories shared by health care workers
- Analysis within the WHO health systems framework

BACKGROUND OF THE NATIONAL HEALTH REFORM

The health system in Lesotho is divided into three levels: the national (tertiary care) level, which includes tertiary hospitals; the district (secondary care) level, which includes filter clinics and district hospitals; and the primary care level, which includes health centers and community health posts.[9] Primary health care is coordinated and led by District Health Management Teams (DHMTs). At the community level, defaulter tracing, adherence support, case detection, and health promotion are provided through Village Health Workers (VHWs). Health centers, which are typically staffed by nurses, provide health promotion, disease prevention, and treatment services with a strong focus on maternal and child health, TB, and HIV services. District hospitals are the immediate referral facility for health centers, provide a higher level of curative care and are staffed by nurses, doctors, and limited specialists.

Prior to the National Health Reform, there were several challenges identified by the MoH at the community, health center, and district levels, which were inhibiting progress towards UHC (listed below). Weak systems for governance and accountability and a lack of essential services at the health center level considerably limited the primary health care system's ability to respond to the needs of the population served

[9]Filter clinics are designed to be the bridge between health centers and referral hospitals, reducing the burden on district and referral hospitals by offering a higher complement of services than health centers.

Figure 2: Lesotho's Health Care Delivery System

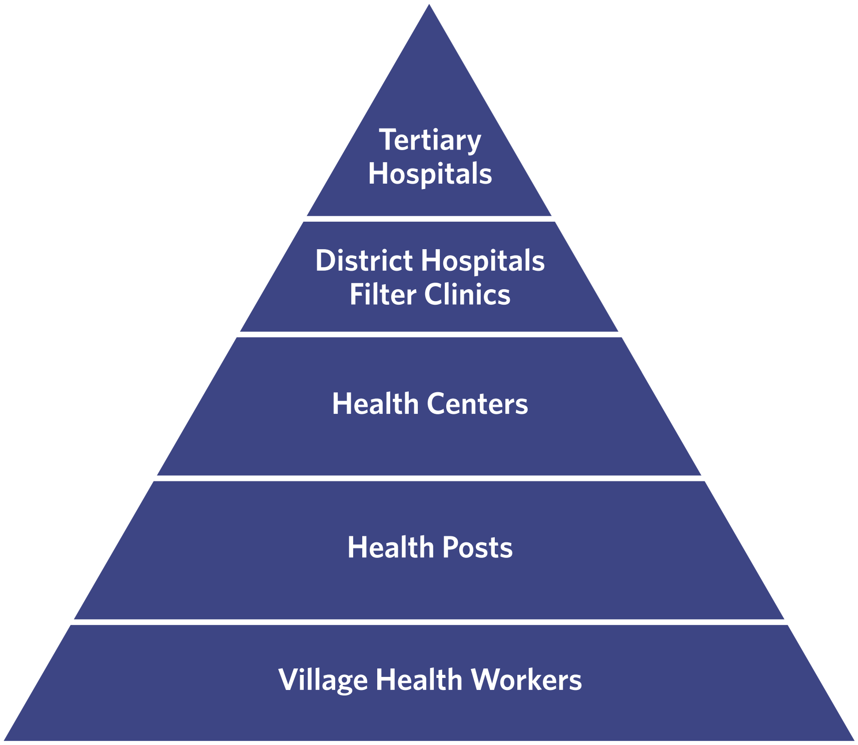

Community Level Challenges:

- Inconsistent number and distribution of VHWs at the village level.
- VHWs had a wide range of responsibilities, including adherence support, case detection, and health promotion, with no proper monitoring mechanism in place.
- Weak system for accountability and supervision of VHWs.
- Accompaniment of patients was not in the job description of VHWs. The lack of patient accompaniment made referrals less effective.
- VHWs had fixed salaries rather than performance-based compensation.
  Health Center Level Challenges
- Weak operability (centers were not fully functional) and a shortage of essential human resources and equipment.
- Low availability of essential medicines, leading to frequent stock-outs of essential medicines.
- Most health centers did not provide a comprehensive package of primary health care services. Availability of facility-based delivery and services for patients with non-communicable diseases (NCDs) were largely missing from most health centers.
- Weak referral system and linkage with hospitals.
- Weak oversight and support from DHMTs.

District Level Challenges:

- No decision-making ability at the DHMT level leading to a disempowered management team (All decisions were made centrally at the MoH level)
- Lack of coordination and organization (unclear roles, poor communication, non-responsiveness)
- Disintegration of governance with multiple reporting lines and lack of clarity on the responsibilities of DHMTs vs. the MoH

The MoH initiated the National Health Reform with close support from PIH serving as a technical advisor to respond to challenges identified and expand progress towards UHC. In April 2014, Phase 1 of the National Health Reform was implemented at all levels of the health system (community, health center, district, and national). Phase 1 targeted four districts, which included a total of 70 health facilities including 63 health centers and seven hospitals, covering an estimated catchment population of 815,520.

The majority of health facilities in the National Health Reform districts are operated by either the GoL (53% (N=37)) or the Christian Health Association of Lesotho (CHAL) 43% (N=30). The remaining three facilities are operated by Red Cross.

Figure 3: National Health Reform Strategies and Levels of Implementation

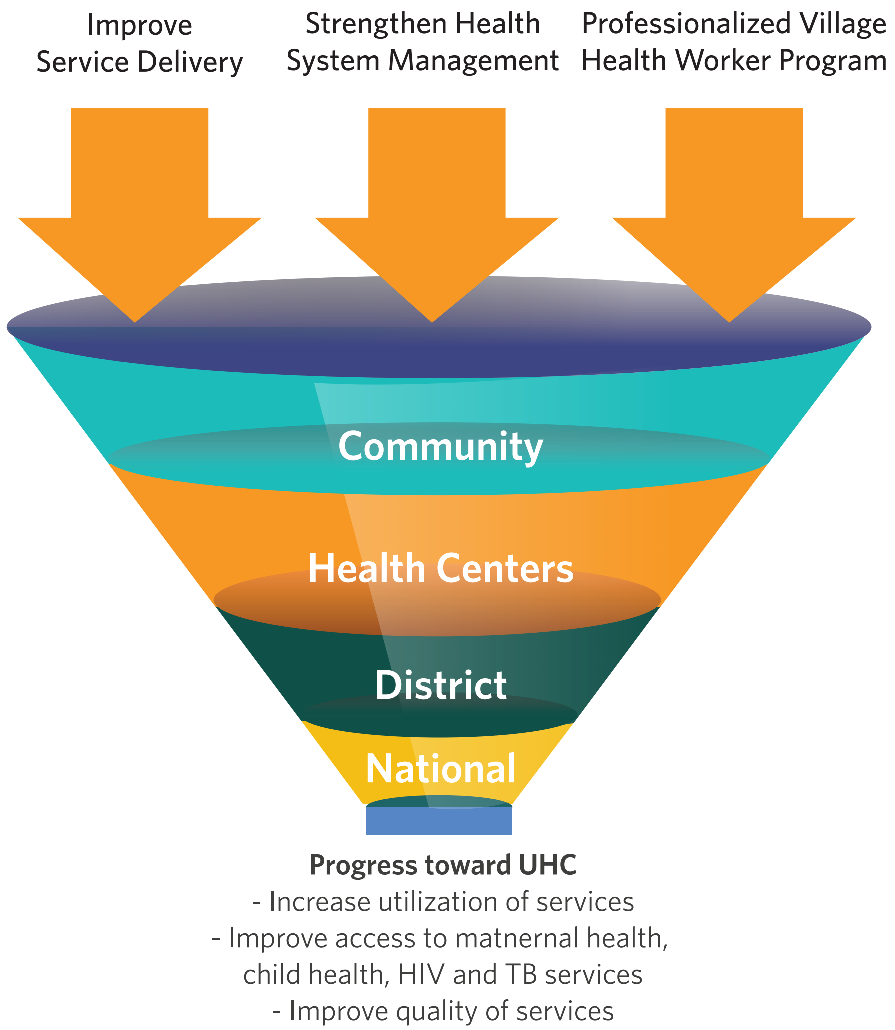

Figure 4: Number of Health Facilities in Reform Districts

District Supported by the Reform	Berea	Butha-Buthe	Leribe	Mohale's Hoek	Total
Health Center	16	10	25	11	62
Hospital	2	2	1	1	6
Total	18	12	26	12	68

The GoL launched the National Health Reform to decentralize and improve the delivery of primary health care services nationally and ultimately to put the country on the path to achieve UHC. The National Health Reform promoted a system-wide improvement of district-led primary health care employing three core strategies: improving service delivery, strengthening health system management, and institutionalizing a professionalized VHW program, see Figure 4.

The service delivery strategy included interventions for improving the mapping of the disease burden of catchment areas and aligning resources accordingly. Training and mentoring of health care workers, initiating essential services, and strengthening systems for referral and supply chain were among the key interventions. Service delivery interventions would not be effective without establishing strong management at the district level. This was achieved through a reorganization of DHMTs, including streamlining reporting and governances among primary health care facilities and empowering districts to make decisions and take full ownership of the running primary healthcare services. This was complimented by efforts to strengthen the VHW program in order to create demand for services and retain those already accessing services. Reporting and accountability structures were set up for VHWs and performance-based incentives were introduced to drive performance.

Figure 5 below describes a detailed list of the types of intervention strategies employed by the National Health Reform.

Figure 5: Core Strategies and Interventions of the National Health Reform

Improved Service Delivery	Strengthened Health System Management	Professional Village Health Workers
• Map disease burden, define and monitor UHC targets<br>• Align inputs with disease burden<br>• Train and mentor health care workers<br>• Initiate essential services at the health center level<br>• Strengthen referral system<br>• Improve availability of human resources<br>• Improve supply chain of essential drugs and supplies	• Decentralize funding and decision-making of DHMTs<br>• Management training for district leadership<br>• Use targets to hold health centers accountable<br>• District and national planning, coordination and review<br>• National technical assistance	• Adequate supervision of VHWs<br>• Clearly defined scope of work for VHWs<br>• Coordination through health centers<br>• Payment through electronic systems<br>• Engagement of civil society and traditional leaders

RESULTS: PROGRESS TOWARD UHC TARGETS

The National Health Reform districts have seen improvements in multiple service delivery areas as a result of interventions across the health system building blocks. Improvements cannot be attributed to one single intervention, rather they are attributable to the comprehensive and crosscutting reforms targeted at transforming the primary health care system. This section presents baseline data (July 2012 to March 2014) along with data from the National Health Reform implementation period (April 2014 to July 2017), compared against progress toward achieving UHC targets on selected indicators, specifically maternal health, child health, and HIV testing and treatment. Data on TB which has been covered in the National Health Reform evaluation has not be further analyzed due to data quality issues. It should be noted that the National Health Reform evaluation has showed that there was no improvement in TB services in reform districts.

Figure 6 shows a summary of key results achieved by the National Health Reform in the areas of maternal health, child health, and HIV testing and treatment comparing quarterly averages from the baseline to the National Health Reform implementation period.

Figure 6: Comparison of Quarterly Averages for Key Indicators Before and After Implementation of the National Health Reform

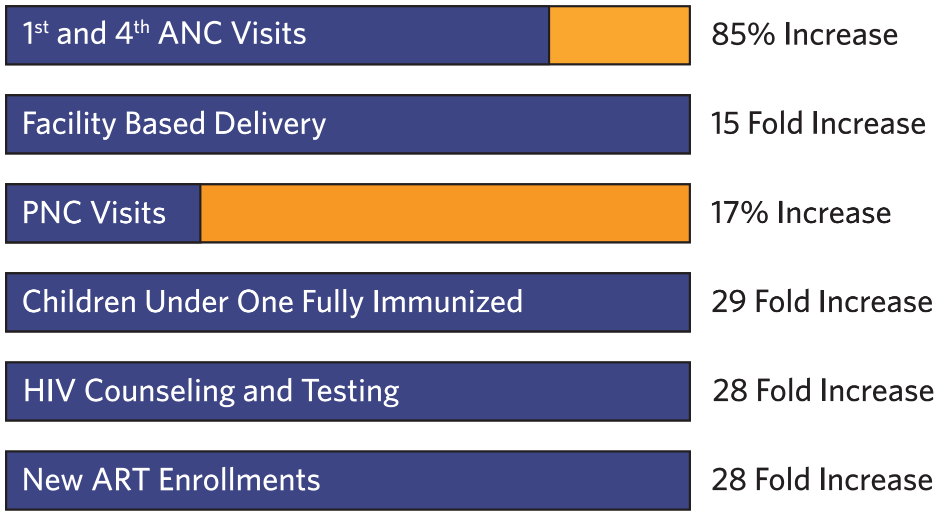

Maternal and Child Health

In this section, key indicators that show access to and retention of pregnant mothers in care, including the first antenatal care (ANC) visits, fourth ANC visits, facility-based delivery, and postnatal care (PNC) visits are presented. Universal access targets for these maternal health indicators were determined by estimating the number of live births per year in the catchment areas of the National Health Reform districts using the crude birth rate.[10]

This section also presents child health data on fully immunized children under the age of one compared to the universal access target for immunization calculated based on estimated surviving infants in the catchment area of the National Health Reform districts.

[10]The crude birth rate is the annual number of live births per 1,000 population. (WHO)

First ANC visits

The first ANC visit is an important indicator used to measure antenatal care coverage and showing access to and utilization of health care during pregnancy. Figures 7 and 8 show the number of pregnant women who utilized antenatal care at the health center level for reasons related to pregnancy at least once during pregnancy.

- The number of women attending a first ANC visit at the health center level increased over the period of the National Health Reform implementation.
- The quarterly average of women attending a first ANC visit increased by 85% from 1,569 at the baseline to 2,908 during implementation.
- Despite the impressive improvements in the National Health Reform period, more progress is required to achieve UHC targets for ANC coverage.
- All National Health Reform districts have seen improvements in the number of first ANC visits compared to the baseline. More women attended a first ANC visit in Leribe District than in other districts. This is expected given the size of the population of Leribe District. The district of Mohale's Hoek saw a lower rate of progress than the other districts in reaching expected number of pregnant mothers in its catchment area.

Figure 7: Number of Women Attending First ANC Visit

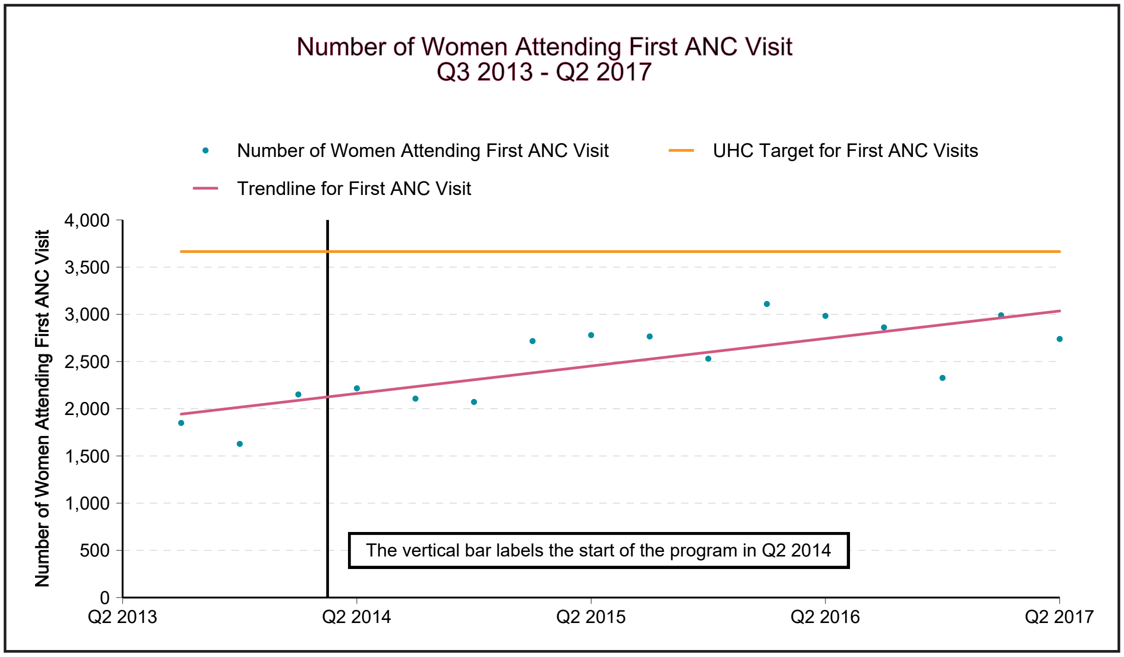

Figure 8: First ANC Visits by District

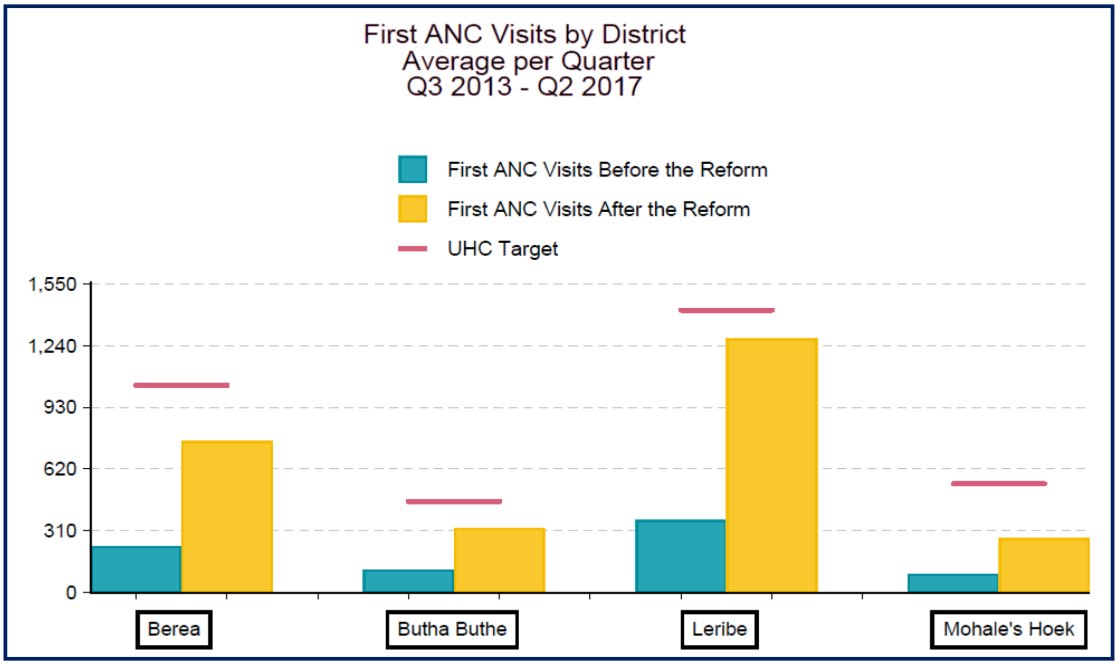

Note: (Figures 7 and 8)
[1] The pink line in Fig 1 represents the line of best fit.
[2] The slope of the pink line in Fig 7 is 72.831.
[3] UHC Target for first ANC Visits in both Fig 7 and 8 are calculated using the total catchment area population for health centers less estimated migration.

Completion of Four ANC Visits

WHO recommends a minimum of four ANC visits, ideally at 16 weeks, 24-28 weeks, 32 weeks and 36 weeks of pregnancy. This indicator measures completion of the four recommended ANC visits, which provide more opportunity for health facilities to monitor progress of pregnancy, assess risk factors, and screen for infections. Figures 9, 10, and 11 present analysis on the completion of four ANC visits at the health center level.

- The quarterly average of women attending a fourth ANC visit increased by 85%, from 613 at the baseline to 1,135 during implementation of the National Health Reform.
- Despite the impressive improvements in the National Health Reform period, performance is far from achieving UHC targets for completion of four ANC visits during pregnancy.
- All districts have seen improvements in completion of fourth ANC visits compared to the baseline, but all were far from reaching their UHC target.
- There is a sizable difference between number of women attending a first ANC visit and those attending a fourth ANC visit, indicating that health centers can improve approaches to retaining women in ANC care.

Figure 9: Number of Women Attending Fourth ANC Visit

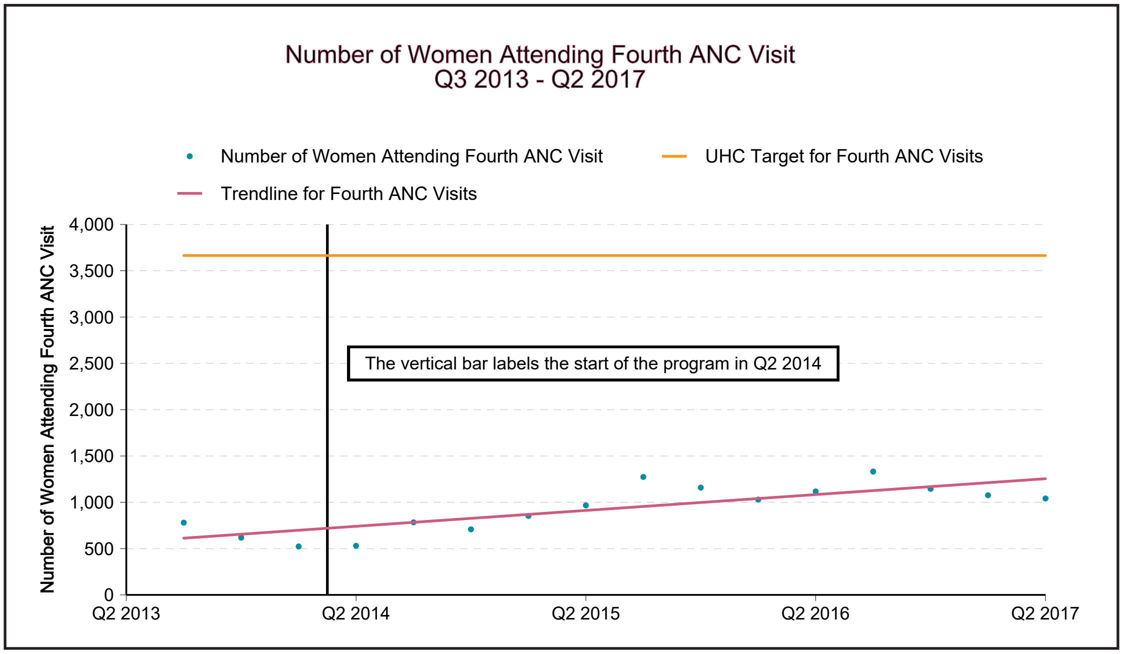

Figure 10: Completion of Fourth ANC Visit by District

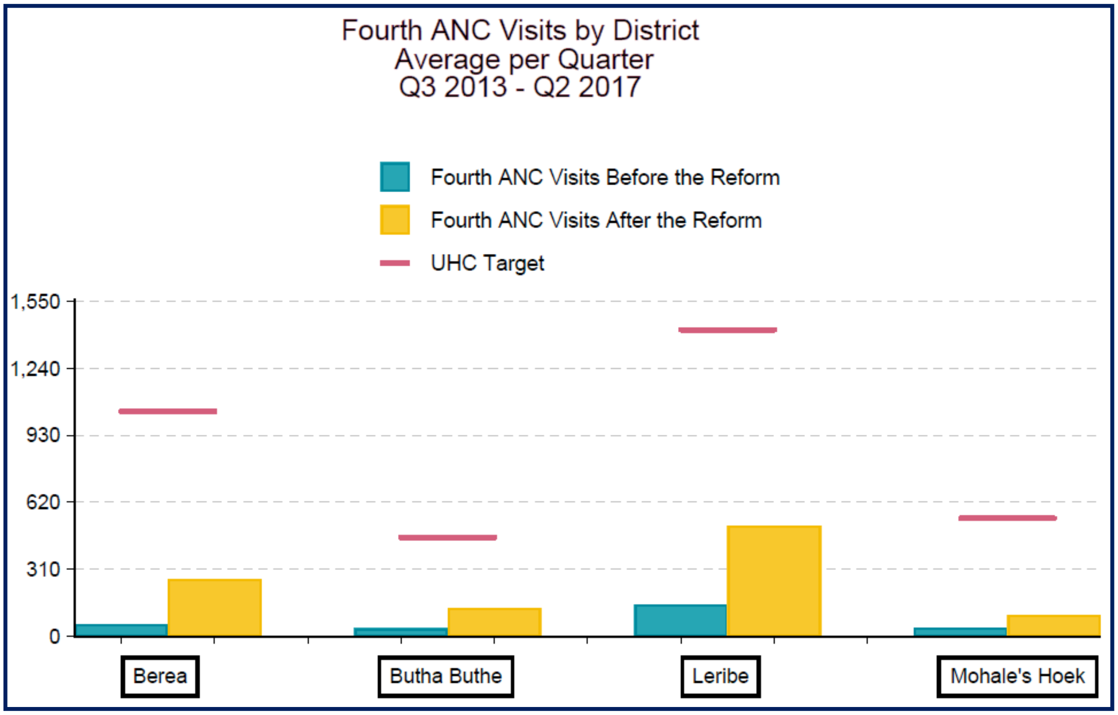

Figure 11: Number of Women Attending ANC Visits

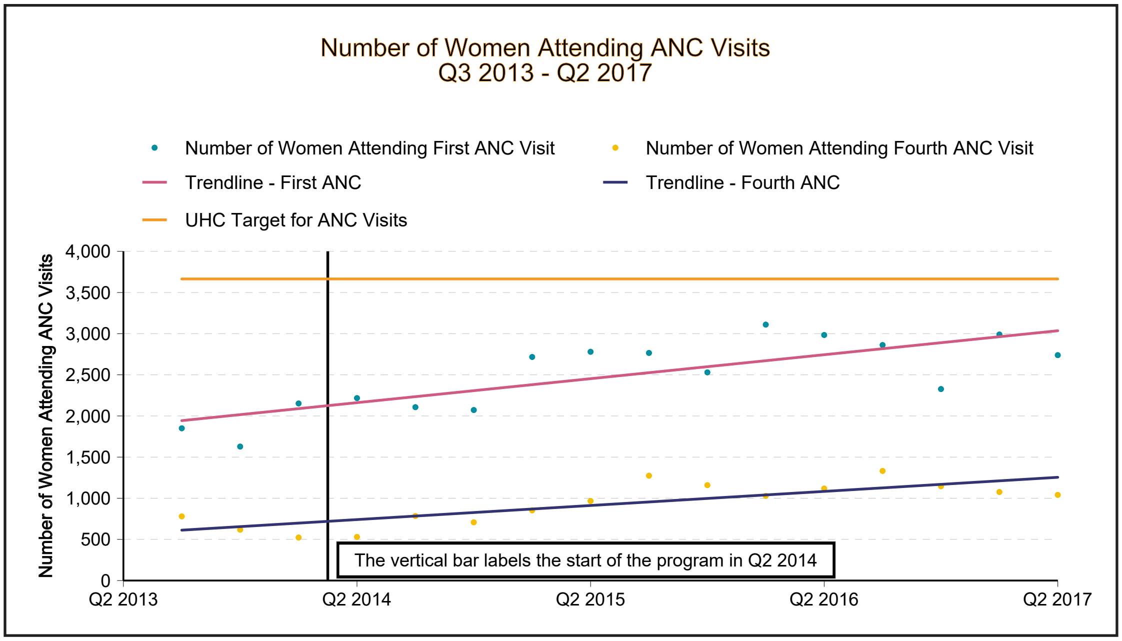

Note: (Figures 9, 10, and 11)

[1] The red line in Figure 9 represents the line of best fit.

[2] In Figure 11, the pink line represents the line of best fit for First ANC visits. The purple line represents the line of best fit for Fourth ANC visits. The slope of the pink line is 72.83. The slope of the purple line is 42.81.

[3] UHC Target for fourth ANC Visits is calculated using the total catchment area population for health centers minus estimated migration.

ANC Visits at Hospitals vs. Health Centers

One of the goals of the National Health Reform is to promote the decentralization of care by improving availability and quality of health services at the health center level. Figures 12 and 13 show a shift in patient flow from hospitals to health centers following the National Health Reform implementation.

- The percentage of first ANC visits at the health center level compared to the hospital level has increased from 75% at the baseline to 80% in the National Health Reform implementation period.
- The percentage of fourth ANC visits at the health center level compared to the hospital level saw an increase of 9%, shifting from 69% at the baseline to 78% in the National Health Reform implementation period.

Figure 12: First ANC Visits at Hospitals and Health Centers

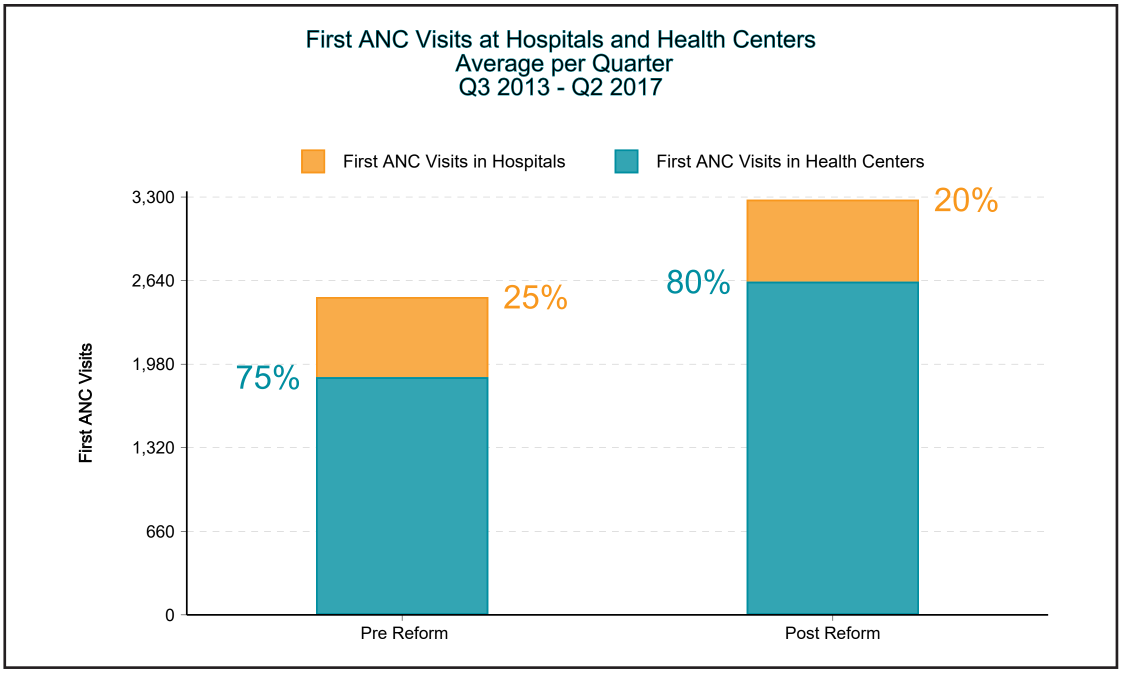

Figure 13: Fourth ANC Visits at Hospitals and Health Centers

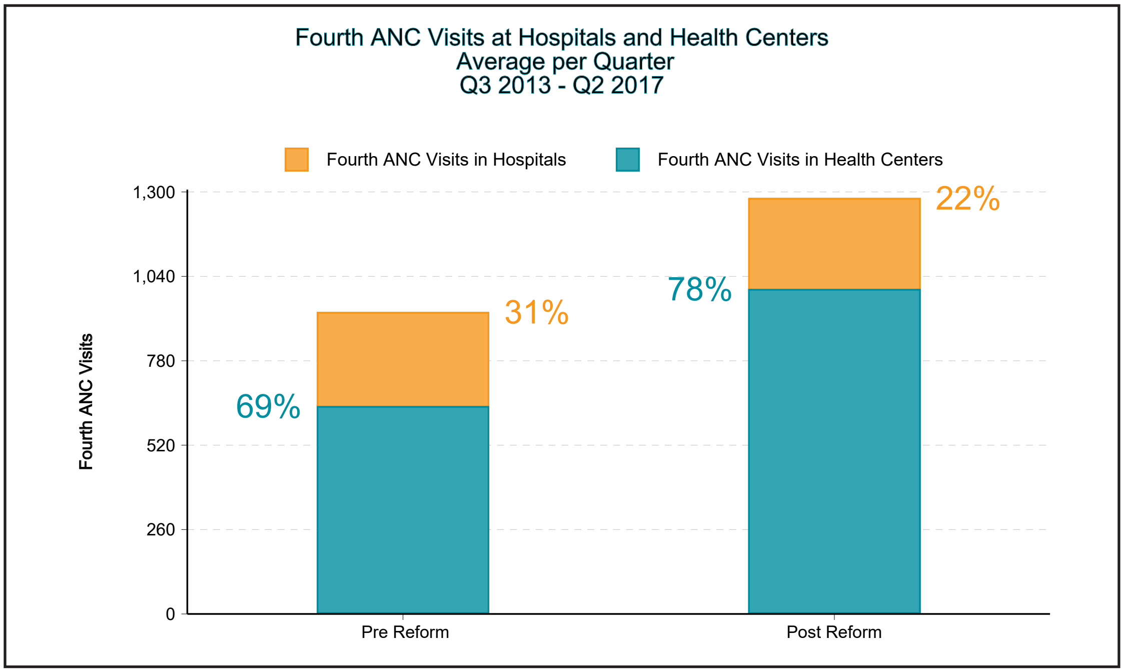

Facility-Based Delivery

Facility-based delivery is one of the high impact health services promoted by the National Health Reform to ensure safe birth, reduce both actual and potential complications, and increase the survival of mothers and newborns. This indicator measures number of deliveries which happen at the health center level attended by skilled nurse midwives. Figures 14, 15, and 16 present data on facility based delivery from National Health Reform districts.

- The average quarterly number of deliveries in health centers increased more than 15 fold from 60 at the baseline, to 952 during implementation of the National Health Reform.
- During the National Health Reform, there was a 30% increase in facility-based deliveries ($p>0.05$).[11]
- There was significant progress in increasing facility-based deliveries compared to the baseline, however, more effort and progress is needed to achieve UHC targets.

Figure 14: Number of Facility-Based Deliveries By District

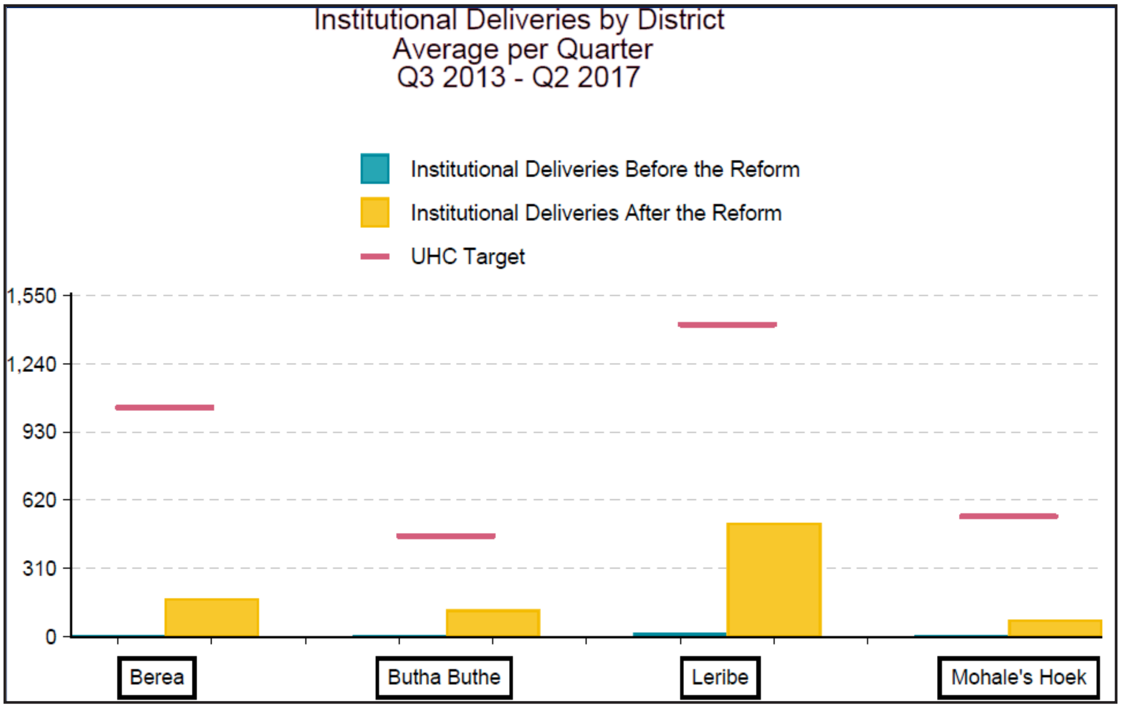

Figure 15: Number of Women Delivering in a Health Center

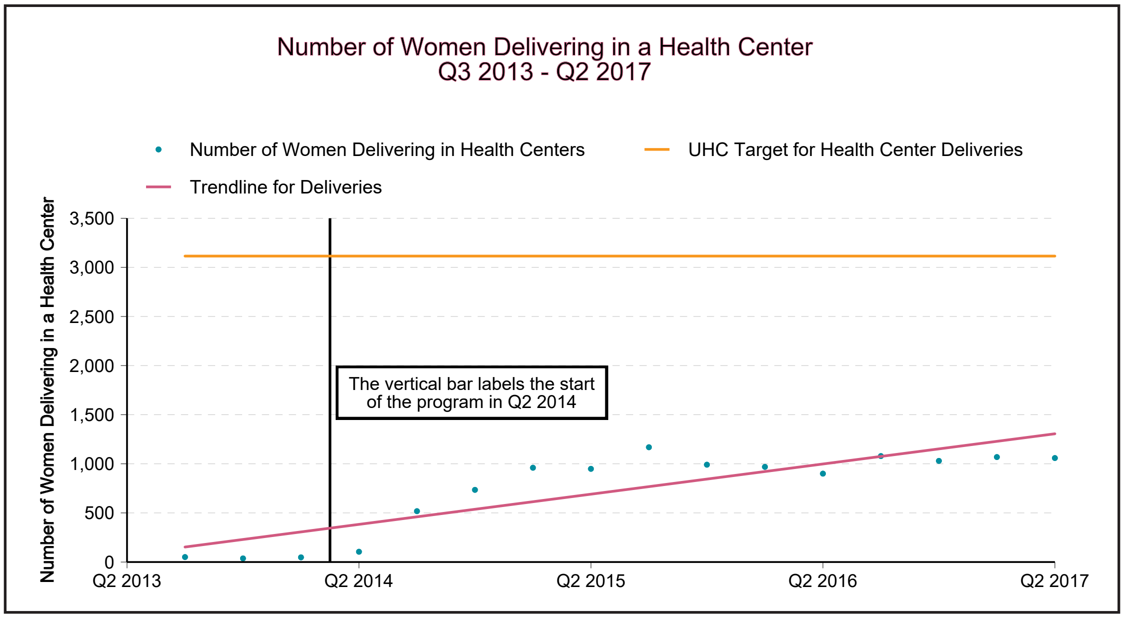

[11] Ministry of Health Lesotho, Partners In Health, Lesotho National Health Reform Evaluation Report, (October 2018)

- All districts have made progress towards increasing facility-based deliveries over the National Health Reform implementation period, with varying degrees of progress against targets.
- The number of deliveries at the health center increased steadily and the number of deliveries at the hospital level slightly declined over the National Health Reform implementation period.
- Institutional maternal mortality during delivery remained low at a rate of 0.03%. There were only three institutional deaths at the health center level out of out of 11,428 total facility-based deliveries.

Figure 16: Facility-Based Delivery and Maternity Mortality

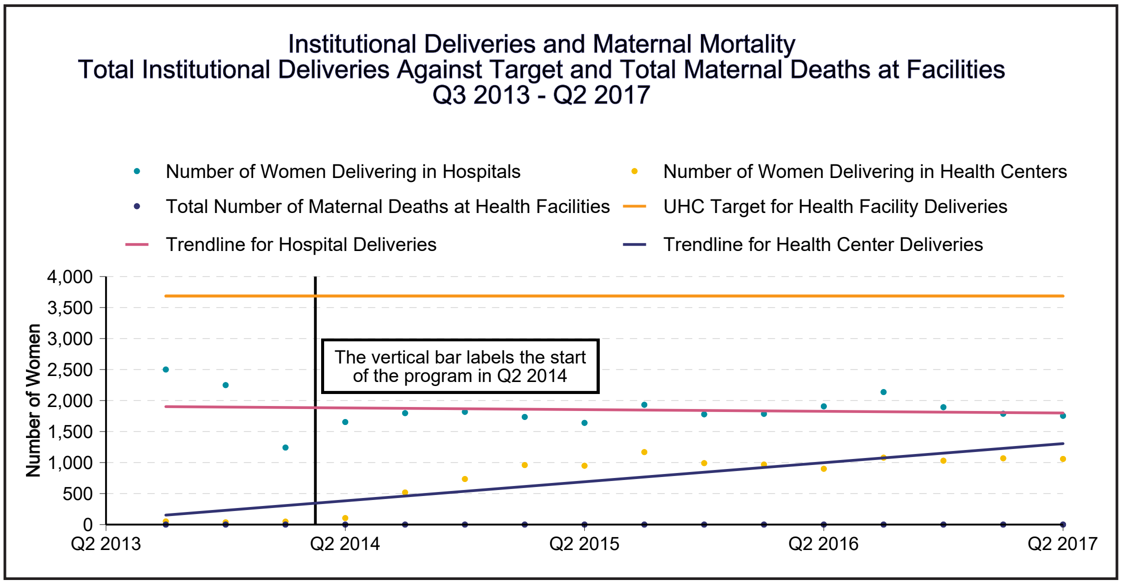

Note: (Figures 14, 15, and 16)
[1] The pink, and purple lines in Figures 14 and 16 represent the line of best fit.
[2] In Figure 16, the slope of the pink line is -6.85. The slope of the purple line is 76.85.
[3] Hospital deliveries are excluded from Figures 14 and 15.
[4] UHC target for health center deliveries is calculated as the total catchment area population for health center facilities minus migration. The total is further reduced by 15% due to estimated number of referrals to hospitals.

Postnatal Care (PNC)

- The quarterly average of PNC visits at the health center level increased by 17% compared to the baseline, showing steady progress towards achieving UHC targets for PNC over the National Health Reform period.
- There was an 8% increase from the baseline in the share of PNC visits at the health center level compared to PNC visits at the hospital level, confirming the trends seen in ANC and facility-based delivery indicators of more pregnant women accessing services at health centers versus hospitals.

Figure 17: Number of Women Attending First PNC Visit

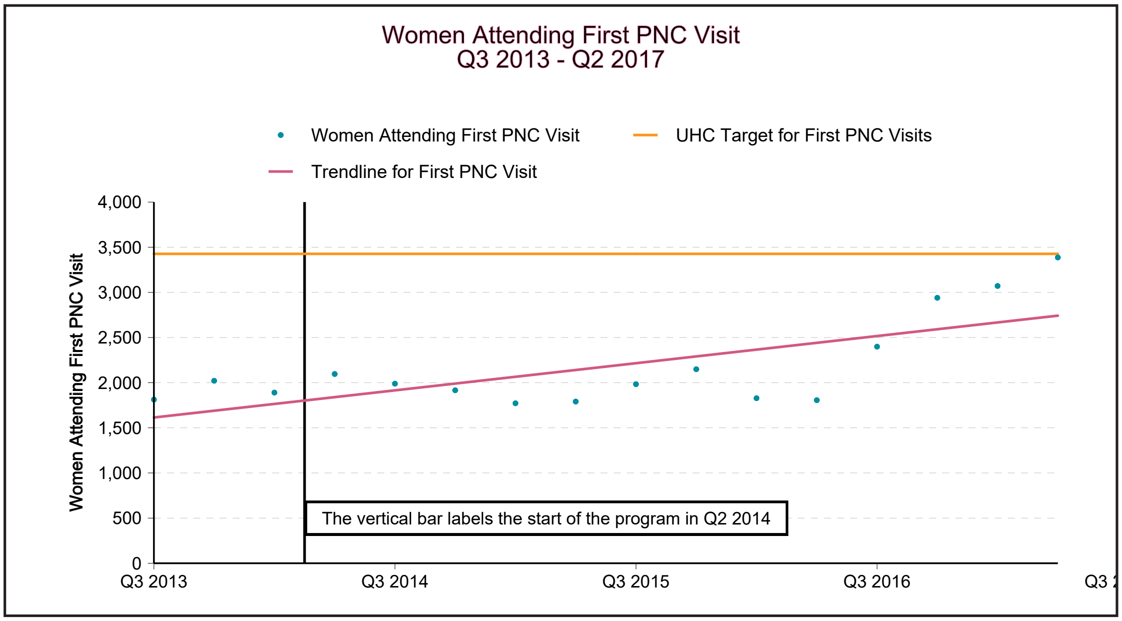

Figure 18: PNC Visits at Hospital and Health Centers

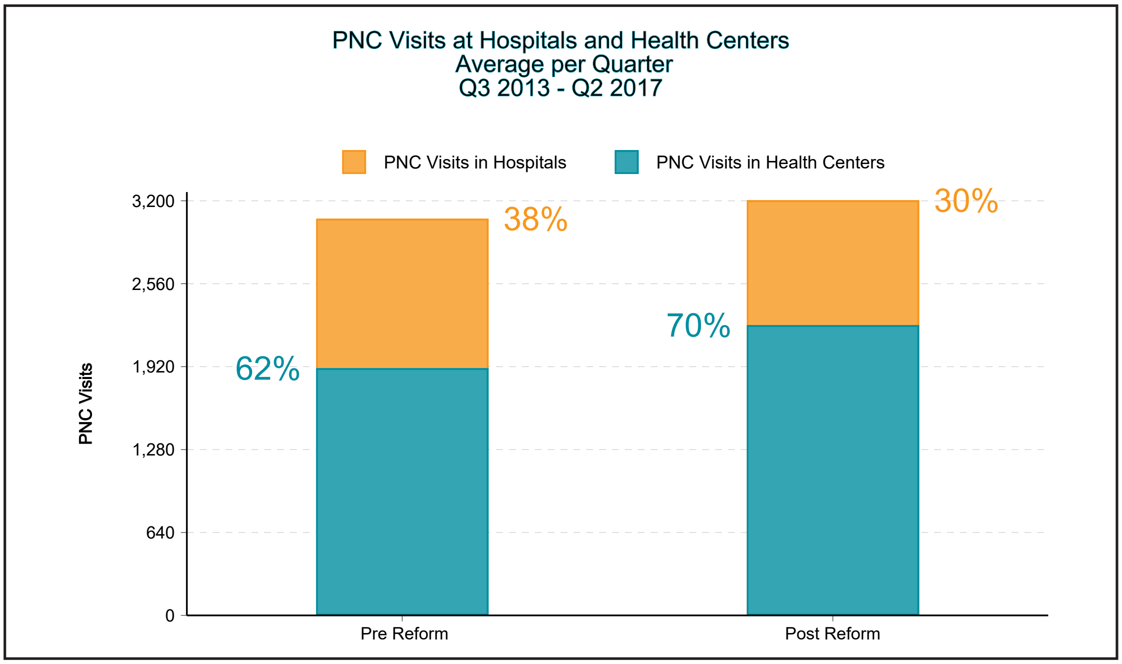

Note: (Figure 17)
[1] The pink line represents best fit, the slope of the pink line is 75.204.
[2] UHC Target for First PNC Visits is calculated as the total catchment area population for health center facilities less migration.

Immunizations

This section presents data on the number of children fully immunized before their firth birthday. The indicator measures the effectiveness of the immunization program to reach all surviving infants with a full course of immunizations. UHC targets for this indicator were calculated by estimating surviving infants in the catchment population of the National Health Reform districts. Figures 19 and 20 present data on fully immunized children under the age of one from the National Health Reform districts.

- There was nearly a threefold increase in the average number of children fully immunized at one year, from 419 at the baseline to 1,196 during the National Health Reform period. However, more progress is needed to achieve the UHC target for this indicator.
- All districts have seen an increase in the quarterly averages of children fully immunized during the National Health Reform period compared to the baseline. Leribe has immunized the greatest number of children due to its larger catchment population compared to other National Health Reform districts.

Figure 19: Children Fully Immunized at Year 1 at Health Centers

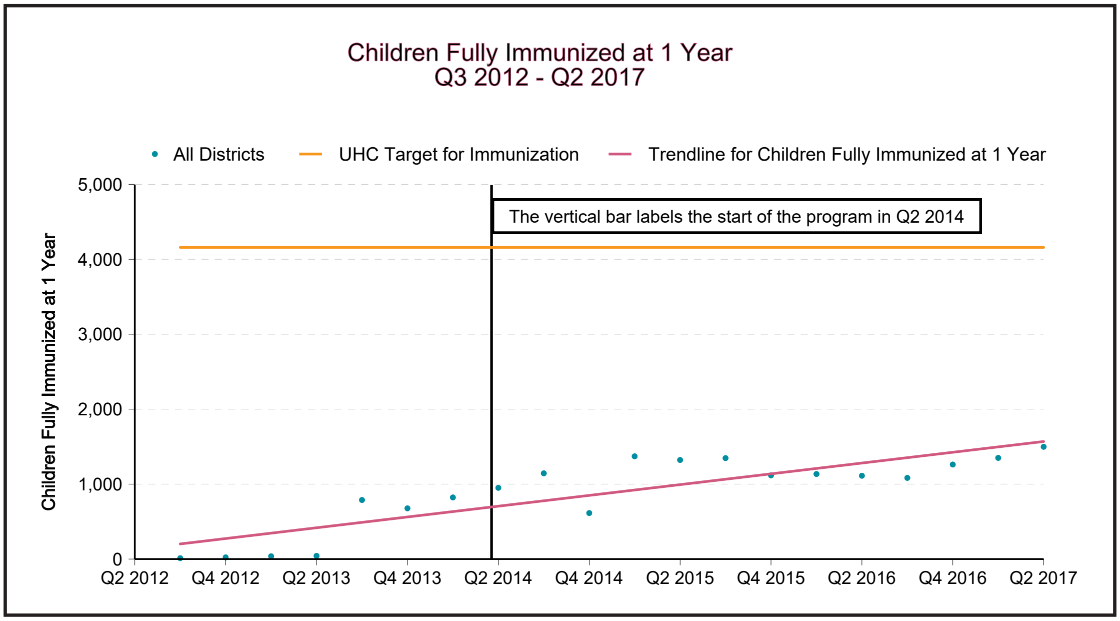

Figure 20: Children Fully Immunized at One Year by District

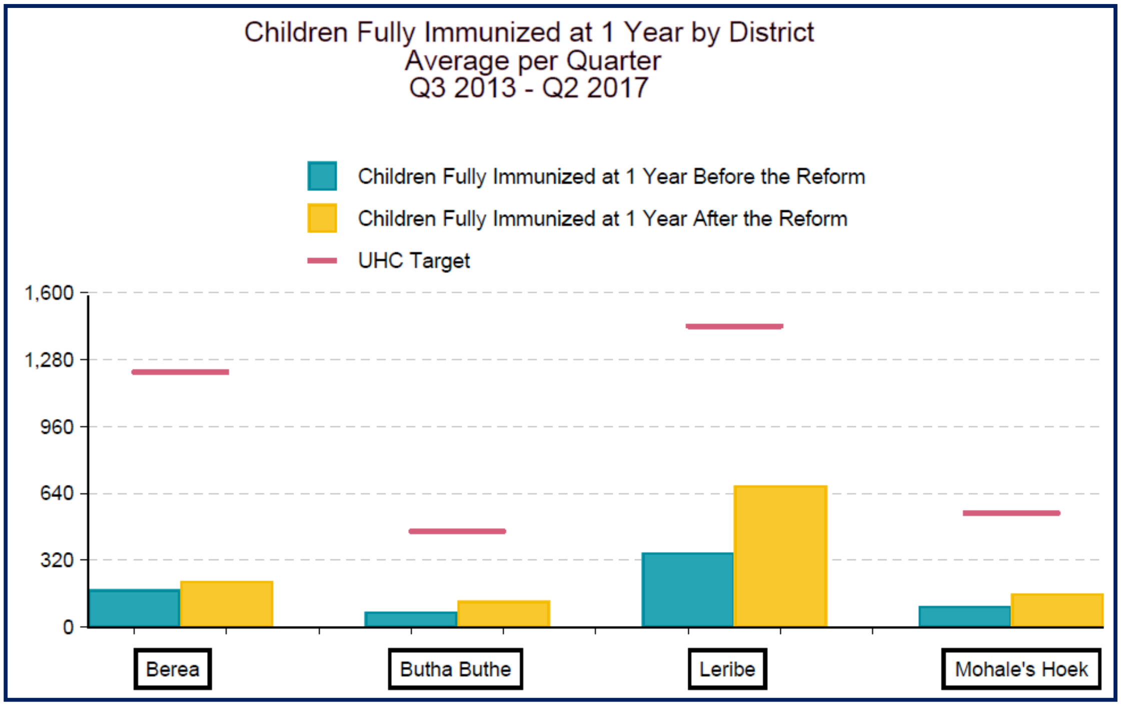

Notes: (Figure 19)
[1] The pink line represents the line of best fit. The slope is 71.940
[2] Excludes hospitals.

HIV

Indicators for HIV testing and new antiretroviral therapy (ART) enrollments at the baseline and National Health Reform period are presented below. UHC targets for HIV testing were calculated by the MoH in line with the 90-90-90 targets for HIV.[12] Figures 21 and 22 present HIV testing and new ART enrollment data from the National Health Reform districts respectively.

HIV Testing and ART Enrollment

- The quarterly average of people tested for HIV at the health center level has nearly tripled, increasing from 14,610 at the baseline to 41,042 in the National Health Reform period, showing considerable progress towards meeting UHC targets. Increase in HIV testing was steady in the National Health Reform period including before and after the start of Test and Treat in Q2 of 2016.
- The quarterly average of new ART enrollments doubled from 1797 at the baseline to 3,684 in the National Health Reform period. New ART enrollments increased markedly after the introduction of Test and Treat.
- New ART enrollments have consistently been higher than the number of positive HIV tests, which can be explained by new enrollees to treatment who tested positive for HIV in previous periods.

[12]The goal of 90-90-90 was introduced by UNAIDS and endorsed by countries in 2013. By 2020, 90% of people who are HIV infected will be diagnosed, 90% of people who are diagnosed will be on antiretroviral treatment and 90% of those who receive antiretroviral will be virally suppressed.

Figure 21: Number of HIV Tests Performed at the Health Center Level

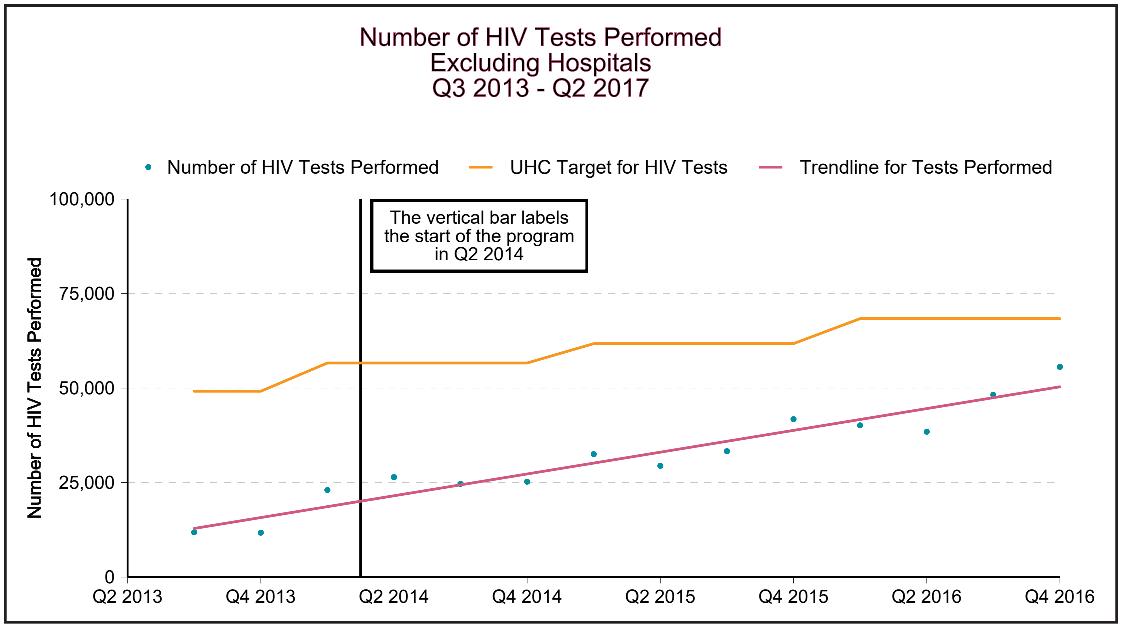

Figure 22: Number of Positive HIV Tests and New ART Enrollments

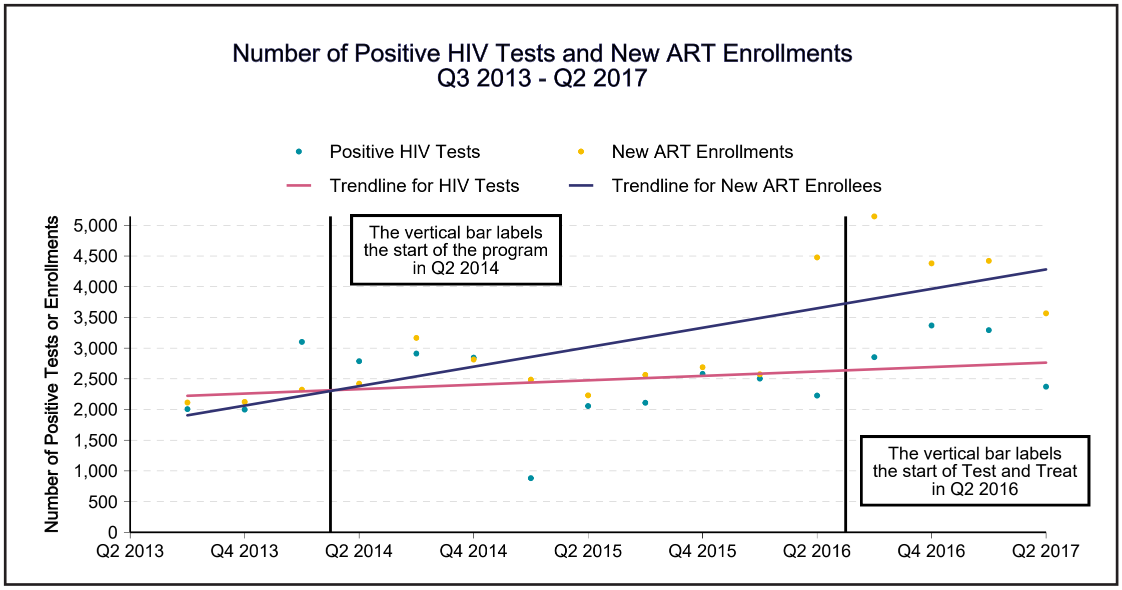

Note: (Figures 21 and 22)
[1] The pink line represents the line of best fit for HIV testing in Figure 21 and positive HIV tests in Figure 22.
[2] The purple line in Figure 22 represents the line of best fit for new ART enrollments.
[3] In Fig 22, the slope of the pink line is 7.13. The slope of the purple line is 39.61.

Viral Load Suppression[13]

- As depicted in Figure 23, all the National Health Reform districts achieved higher viral suppression comparing 2013-2014 levels to 2016-2017 levels.
- National Health Reform districts represent four out of the top six districts for viral load suppression nationally.

Figure 23: HIV Viral Load Suppression by District

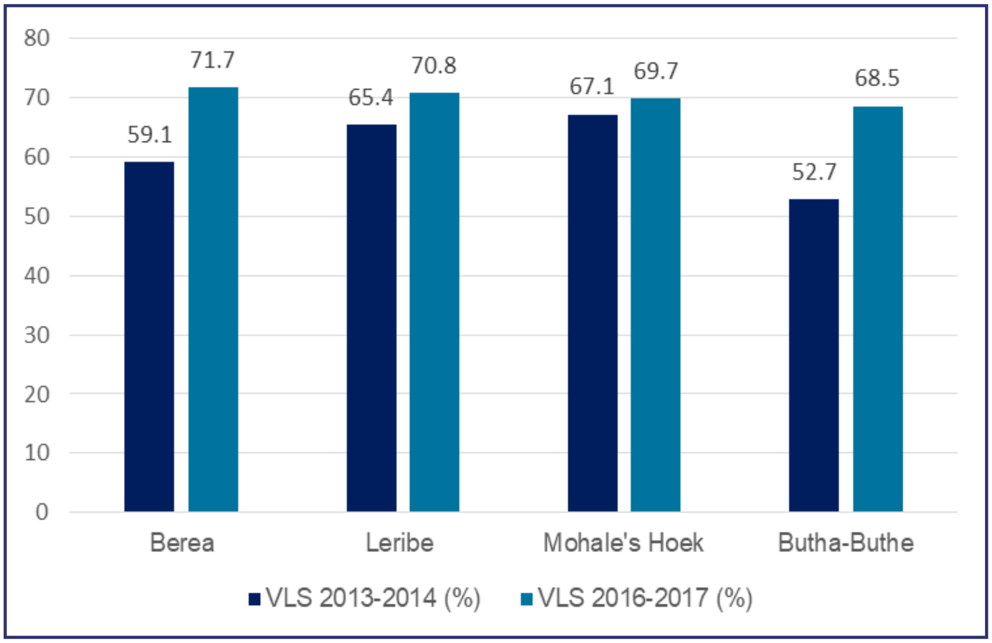

Key Takeaways

- The results consistently show an upward trend in selected maternal health, child health, and HIV testing and treatment indicators after the implementation of the National Health Reform (April 2014 to July 2017) compared to the baseline (July 2012 to March 2014). Notable and significant changes such as a 30% increase in facility-based delivery is expected to have a high impact in reducing the high maternal mortality rate in Lesotho. Despite these improvements, the results also show that there is a need for more progress in order to achieve UHC targets.
- Increasing trends across various program areas, including ANC, facility-based delivery, PNC, child immunizations, HIV testing, and ART enrollment, confirm that interventions targeted at overall health system strengthening can improve multiple service delivery areas at the same time.
- Another consistent trend is the shifts in patient flow from hospitals to health centers, indicating better service delivery at the health center level after the National Health Reform. It also shows the value of providing services closer to the community, reliving the burden of routine care from hospitals and allowing them to focus on more advanced care services.
- Variability of results among National Health Reform districts is evident in most of the indicators presented. This warrants a deeper investigation of what drives such variances.

---

[13]Data on viral load supersession was taken from Lesotho Populations HIV Impact Assessment (2017) and Lesotho Demographic Health Survey-Supplement on HIV Viral Load (2014)

NATIONAL HEALTH REFORM INTERVENTIONS

This section will explore how the results presented above were achieved, presenting key interventions of the National Health Reform and associated data on implementation, using the WHO health systems framework to guide the discussion. Illustrative quotations from interviews with health care workers reporting change stories of each health system building block are included in each section, demonstrating the value of the National Health Reform interventions from the perspective of health workers at the community, health facility and district levels.

The WHO has defined a framework for health systems, comprised of six building blocks, which is designed to address the underlying factors and processes required to run an effective health system. One key purpose of the framework is to create common understanding of what a health system is and what constitutes health systems strengthening.[14] We have included a seventh block to highlight the substantial contributions of the National Health Reform to community engagement. Figure 24 outlines and defines the seven building blocks and framework. WHO's health systems framework is not designed to be selectively used for a particular disease or public health issue, rather it is designed to achieve UHC. Therefore, the framework is appropriate for analyzing the interventions of the National Health Reform and their influence on strengthening the primary health care system.

Figure 24: Health Systems Building Blocks, Adapted from the WHO Health Systems Framework

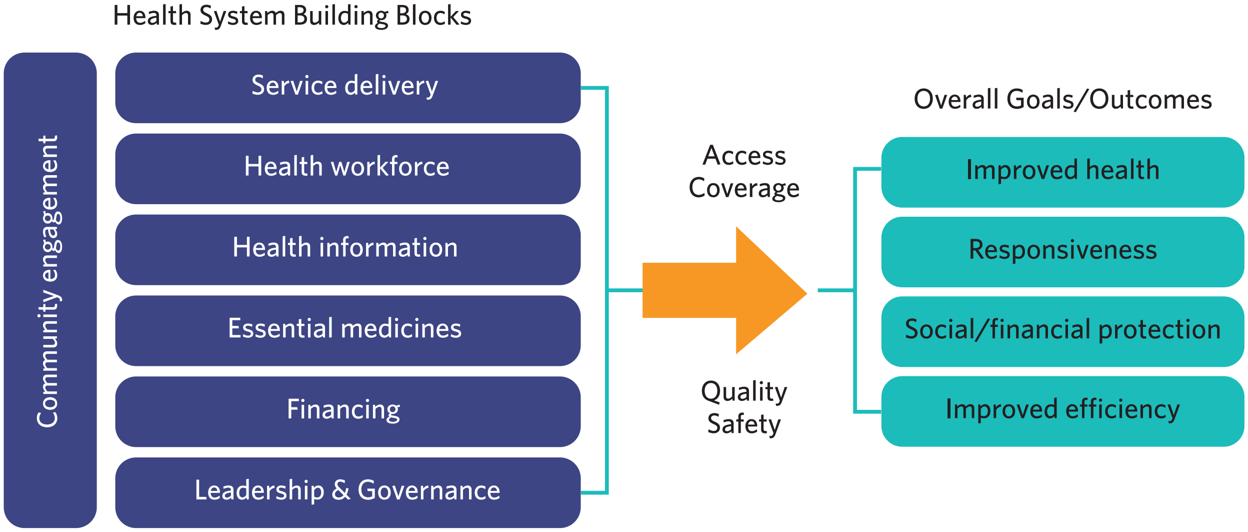

[14]Everybody business: strengthening health systems to improve health outcomes: WHO's framework for action. (WHO 2007)

- **Service delivery** – Effective, safe, quality health services to those who need them.
- **Health workforce** – Sufficient number, mix of staff, and a competent, responsive, productive, and fairly distributed health workforce.
- **Medical products and technologies** – Equitable access to safe, quality assured medicines and technologies.
- **Financing** – Social health protection, increased financing, equitable and efficient allocation of funds.
- **Information** – Reliable and timely information.
- **Leadership and governance** – Evidence-based policies and strategies, oversight, and accountability.
- **Community engagement** – Responsive to community needs, community and individual involvement.

Figure 25 summarizes the key outputs of the interventions employed by the National Health Reform compared to the baseline.

Figure 25: Comparison of Key Indicators Before and After Implementation of the National Health Reform

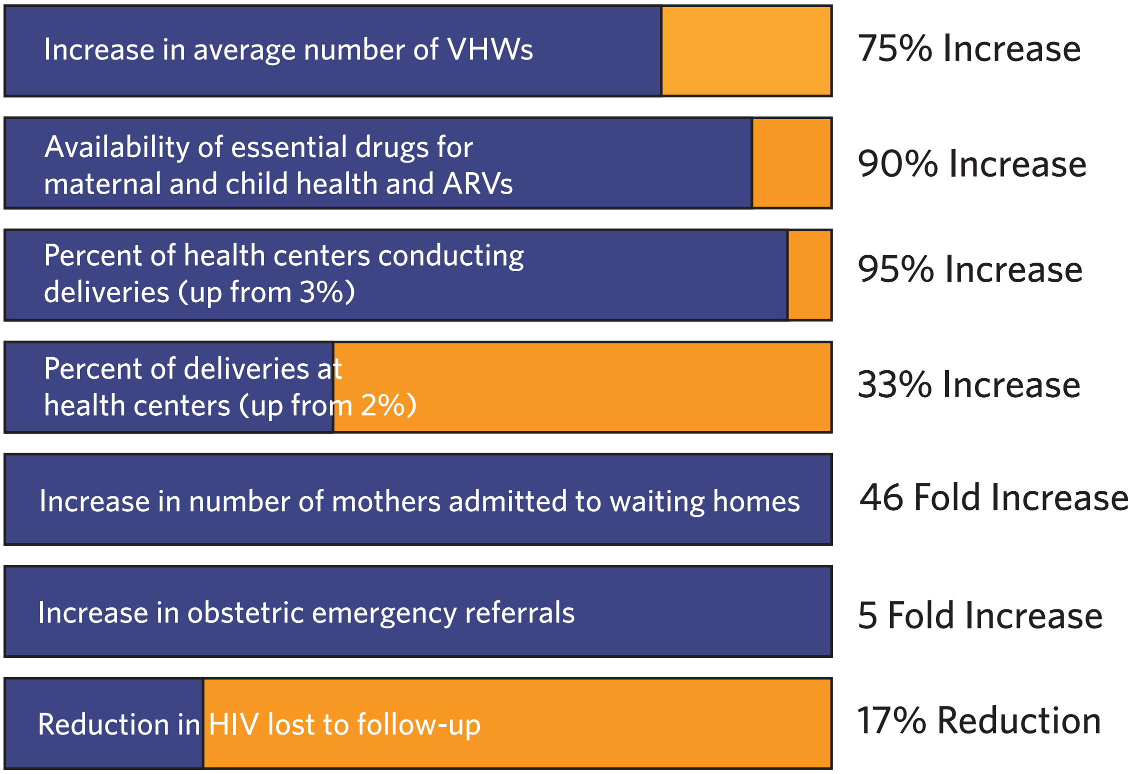

SERVICE DELIVERY

The National Health Reform transformed service delivery in primary health care facilities by providing support in three key areas:

- Equip facilities to initiate new services
- Establish a system for ongoing mentorship and supervision
- Strengthen referral mechanisms

Equip Facilities to Initiate New Services

Key Results

— Increased the percentage of health centers equipped to provide facility-based deliveries from 3% to 95%.
— Increased proportion of facility-based deliveries that happen at health centers compared to hospitals from 2% to 33%.
— Initiated services for non-communicable diseases (NCDs).

Figure 26: Facility-Based Deliveries at Hospitals and Health Centers

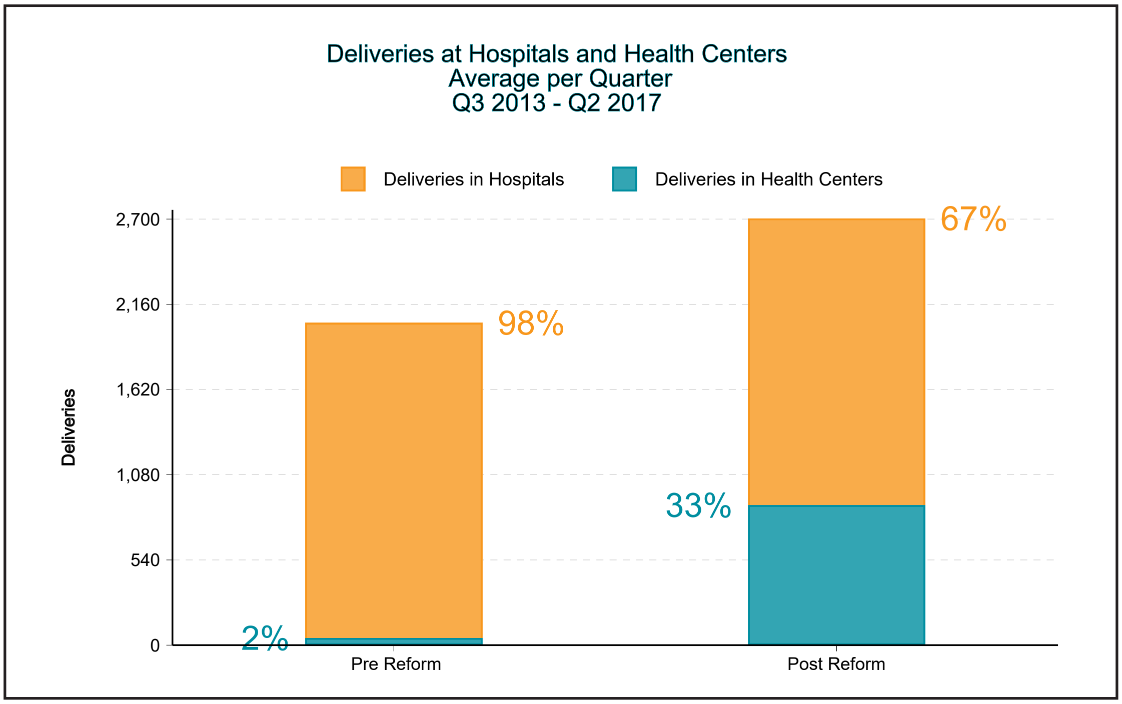

Facility-Based Delivery

The National Health Reform initiated essential primary health care services that were not previously provided at the health center level, mainly the initiation of life saving labor and delivery services targeted at reducing maternal mortality.

During the National Health Reform, the percent of health centers equipped to provide facility-based deliveries increased from 3% to 95% and the share of deliveries at the health center level increased from 2% to 33% (see Figure 26). There has been a significant increase in the overall number of facility-based deliveries after the National Health Reform in addition to the shift in facility-based deliveries from the hospital to health center level. There was a 30% increase overall in institutional deliveries ($p>0.05$) and the quarterly average of health center facility-based deliveries increased 15-fold.

> *"When I arrived in 2014, it was only four facilities out of twenty-six that were conducting deliveries… the purpose of Reform in terms of service delivery was to introduce facility deliveries in all the health centers in Leribe and we achieved that."*
>
> *Leribe District*

The provision of required equipment and capacity building training of staff enabled health centers to start providing delivery services. Disposable delivery packs were provided to health centers, without which deliveries would not have been possible. Delivery packs contain; drapes, disposable gloves, umbilical clamps, disposable scalpel, gauze sponges, and more items which required sterilization. Disposable delivery packs addressed the lack of autoclave machines to sterilize delivery equipment and prevent infections, helping to ensure high quality of care during delivery.

> *"We manage to maintain sterility or to prevent cross infection by all means possible because availability of sterile disposable packs means we use the packs for a particular patient then you discard thereafter. If such equipment was not available, there is no way we can do deliveries"*
>
> *Leribe District*

Maternal Waiting Homes

Key Results

— The number of pregnant women residing in health center maternal waiting homes before their due date increased 46-fold ($p<0.05$) after the National Health Reform in the four National Health Reform districts.

In addition to equipping facilities with the skilled human resources and medical equipment necessary to provide safe facility-based deliveries, the approach used during the National Health Reform also worked to improve utilization by addressing barriers to accessing the service.

One of the main factors preventing mothers from delivering in health facilities is the long distance they often must travel to reach the health facility. A sample of mothers in maternal waiting homes (n=35) taken from high volume sites in the four National Health Reform districts showed that most mothers in maternity homes reside a 2 to 4 hour walk from the health center.

Figure 27: Average Distance from Home to Health Center for Expectant Mothers in Maternal Waiting Homes

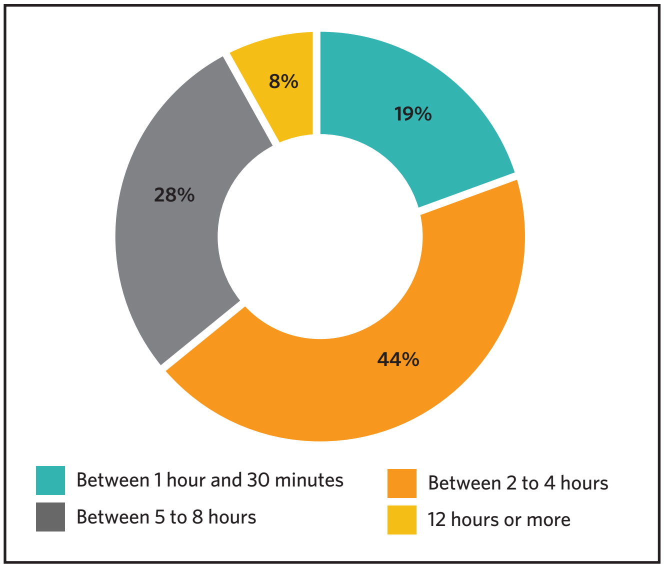

Maternal waiting homes were made functional in health centers to address this challenge. All pregnant women who are 38 weeks and above of gestation and reside far from the health center as well as those with identified risk factors were admitted to maternal waiting homes. With support from the National Health Reform, these mothers were able to reside in the maternal waiting home until delivery, and provided with food for the duration of their stay. Provision of such items provided a safe and comfortable environment for pregnant mothers and encouraged more mothers to utilize maternal waiting homes.

> *"Our terrain is very difficult, and for somebody who will walk for more than three hours just to go and deliver at the health center, even me, I will deliver at home if I have to walk for such a long time. But with the Reform, [...] and we could make it, because the women could stay in the health center until their delivery. And if they have complications, they are being referred on time to the hospital"*

The number of mothers residing in maternity waiting homes as well as the percentage of total deliveries from mothers residing in maternity waiting homes has risen consistently over the National Health Reform period, demonstrating the critical role maternal waiting homes play in increasing institutional deliveries as shown in Figures 28 and 29. The share of maternal waiting home admittances to number of deliveries at the health center has increased from 21% at the baseline to 50% in the National Health Reform period.

Figure 28: Percent of Pregnant Women Admitted to Maternal Waiting Homes

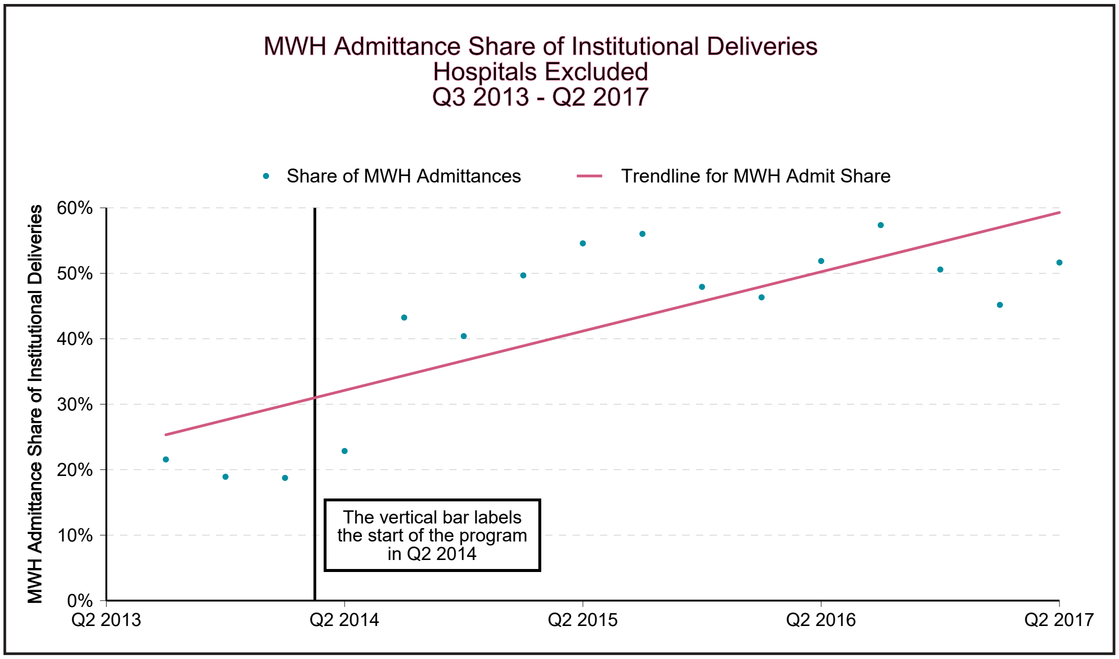

Figure 29: Number of Pregnant Women Admitted to Maternal Waiting Homes

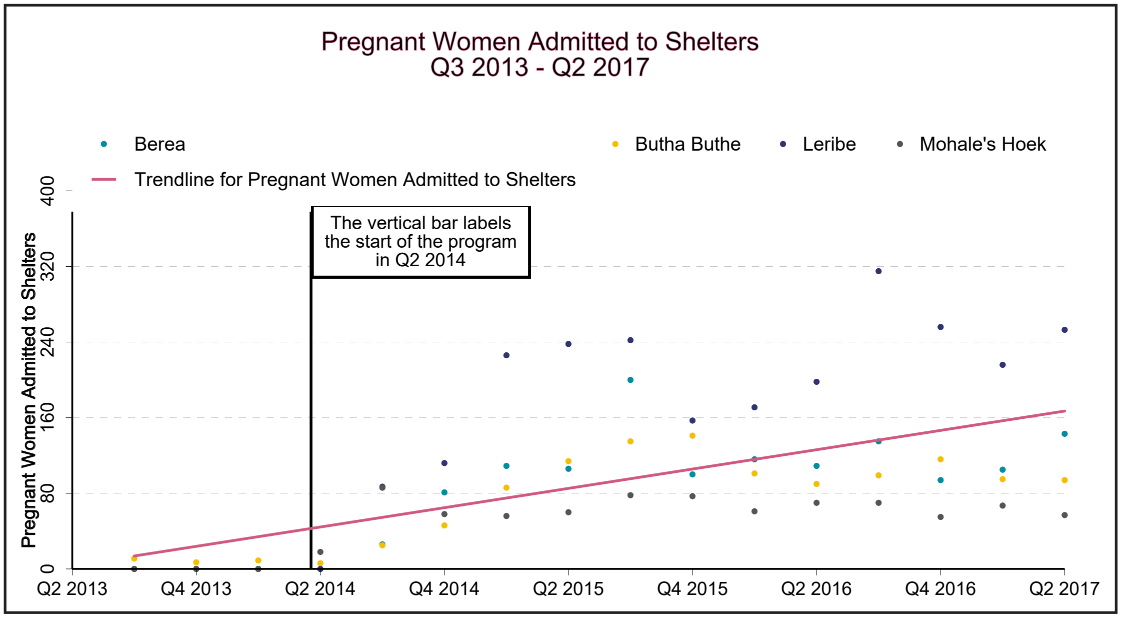

Note: (Figures 28 and 29)
[1] The blue line in Figure 28 and the pink line in Figure 29 represent the line of best fit.

Non-Communicable Diseases (NCDs)

Chronic care for patients with NCDs was initiated in 2015 in primary health care facilities in Berea District. The double disease burden in Lesotho of communicable and non-communicable diseases demands that primary healthcare facilities provide NCD services. As shown in Figure 30, data from 12 health centers in Berea District shows a wide range of NCD services being provided to 4,698 active NCD patients from January 2016 to June 2019.

Figure 30: Number of Clients Active in the NCD Program in Berea District (January 2016-June 2019)

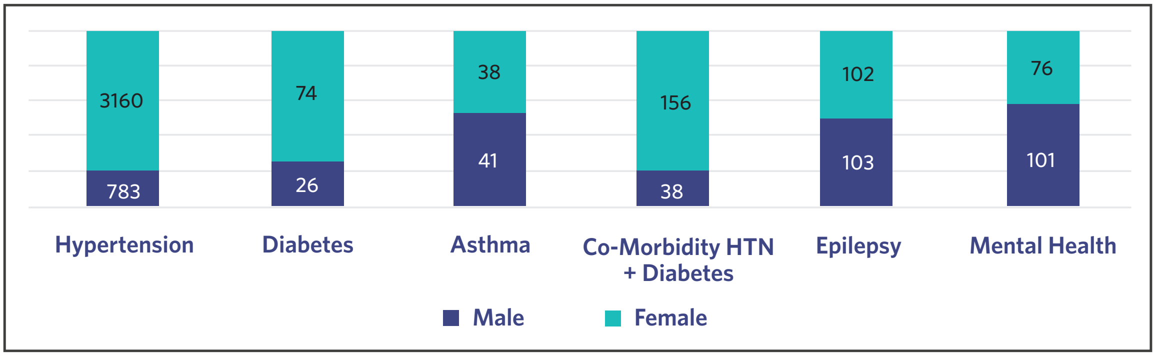

Mentorship and Supervision

Key Results

— When comparing data from (2016-2017) to the period leading up to the National Health Reform, supervisions showed a 40% increase. There are now one to two supervision visits from DHMTs per month per health center.

Systems for mentorship and supervision were set up to improve existing and new services and build the skills and knowledge of health care workers at the health center level. Mentorship and supervision standards were developed and DHMTs were trained on new approaches. The new approaches set standards for periodic visits by DHMTs to health centers and also developed checklists for focused mentorship and supervision visits. The PIH-L team provided consistent support to DHMTs including: logistics and provision of updated guidelines, standard operating procedures, registers, and reporting tools. Mentorship covered a wide range of areas including, maternal and child health, HIV, TB, and NCDs.

"Reform improved our standards on supervision. We were trained on supervision as managers, and we were encouraged to go out almost every week to conduct super-vision to [...] facilities on the community program and maternal and child health and other areas like ART and HIV testing. [....] [this was] improving the indicators that are priority for the Ministry of Health"

Leribe District

Strengthening Referral Mechanisms

Key Results

— Obstetric emergency referrals increased over 500%, and continue to increase (P<0.05).

Health centers are not equipped to manage all complications during pregnancy and childbirth. Specifically, they are not equipped to perform caesarian sections or blood transfusions. Complications during pregnancy and labor that require advanced care would require the patient to be transferred to a hospital using an emergency referral mechanism. Nurse midwives are trained to identify such complications and timely refer pregnant mothers to the hospital. The National Health Reform established an obstetric emergency referrals mechanism that uses local business cars to transport mothers with complications from health centers to hospitals. Prior to the reform, there was no ambulance service or alternative emergency transportation mechanism to transfer patients in case of an emergency. An emergency transportation mechanism provides transportation for pregnant mothers who require an urgent medical response at a hospital for definitive care (see Figure 31).

In addition to providing a lifesaving alternative for mothers experiencing emergencies, the transport mechanism increased community participation and encouraged local businesses to partner with health centers.

Figure 31: Emergency Referrals During Pregnancy and Labor from Health Center to Hospital

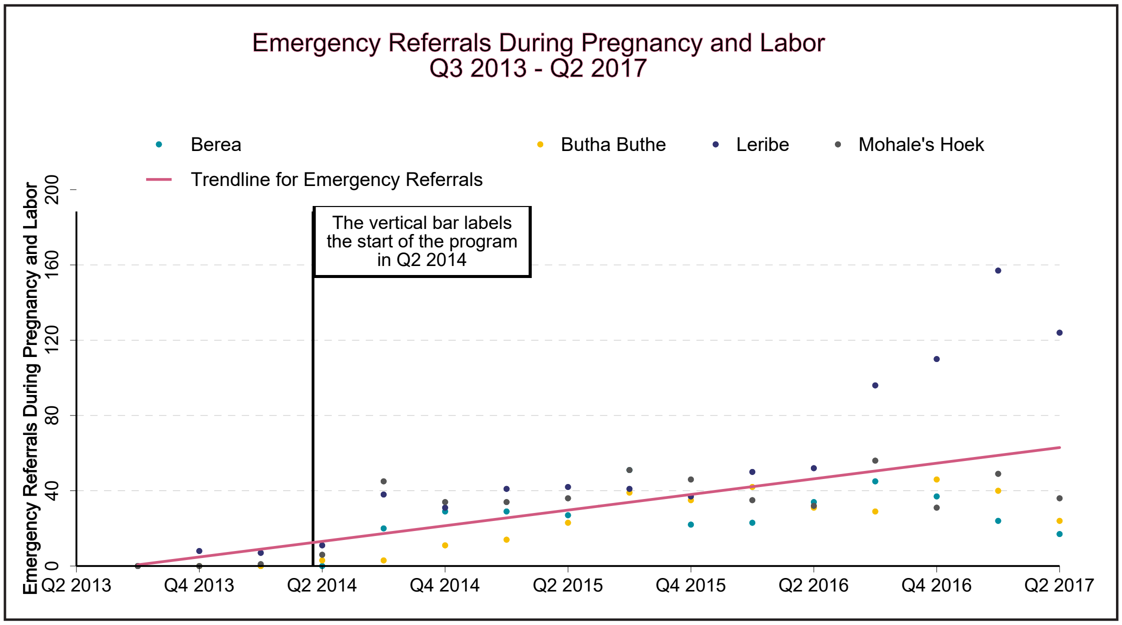

Note: (Figure 31)
[1]The pink line represents the line of best fit for emergency referrals. The slope of the red line is 4.153.
[2] Excludes hospitals.

> *"Use of local transport makes the community to be part of what is happening, as a local business man from the community use their cars for transport that will make them support the services of the clinic, [which] was good"*
>
> *Butha-Buthe District*

COMMUNITY ENGAGEMENT

The National Health Reform greatly enhanced community engagement by strengthening the Village Health Worker Program through the following interventions:

- Increased the number of VHWs in communities to expand their reach
- Established accountability and governance by introducing VHW supervisors and coordinators
- Developed protocols and guidelines for the VHW program and formalized training programs
- Introduced performance-based incentives to improve performance of VHWs

Increased Number of VHWs

At the start of the National Health Reform, information on the number of VHWs working in a given district was not up-to-date; some VHWs had died, while others had moved from the area or were no longer active. During the National Health Reform, the database for VHWs was updated to identify the correct number of VHWs in each village. In addition, the number of VHWs was increased to expand their reach, meeting the standard of one VHW per 20 households. This standard was estimated taking into account duties and responsibilities of VHWs and the specific context of Lesotho.

> *"We have places that are far from the clinic but since Reform and the increase in the number of VHWs, we saw women from these places coming because VHWs would encourage them to come deliver at the clinic"*
>
> *VWH, Butha-Buthe District*

Accountability and Governance Structures

The National Health Reform introduced a VHW Supervisor role to improve program monitoring and oversight of the VHWs at the community-level. VHW Supervisors were selected from the cadre of active VHWs who had higher levels of education and were responsible for managing VHWs, supervising their work, and collecting reports. The National Health Reform also introduced a VHW Coordinator role based at the health center to serve as a bridge between the VHW program and the health center. VHW Coordinators were responsible for providing overall coordination for the VHW program, collecting and verifying reports from VHW Supervisors, and providing feedback, guidance and mentorship to VHWs.

"Reform put supervisors for village health workers which they report to, who in turn will report to the village health worker coordinator at the facility level. So that structure was put in place for the village health worker program to ensure that there is also smooth… ladder, for reporting."

"It's true there are village health workers throughout the country. But they were not monitored well. With the Reform, we noticed that the monitoring was very good, because they had supervisors, they had a coordinator who is based at the health center"

Mohale's Hoek District

Improved VHW Guidelines

Guidelines detailing the objectives, governance structures, approaches, and key activities of the VHW program were developed to define strategies for continuous improvement, reporting and monitoring, and incentive structures for VHWs. VHWs were categorized in two specialized cadres, one group focusing on TB/HIV and the other focusing on maternal health issues called the Maternal Mortality Reduction Program Assistants (MMRPAs) (see Figure 32). Key activities identified in the new guidelines for VHWs included active case finding, accompaniment of patients, defaulter tracking, and planning and leading community-based education sessions. VHWs in all National Health Reform districts were trained based on the new guidelines. The number of VHW accompaniments and instances of effective defaulter tracing were among the most impactful interventions achieved by the VHW program during the period of the National Health Reform.

Figure 32: Summary of Guidelines for TB/HIV and MMRPA VHWs

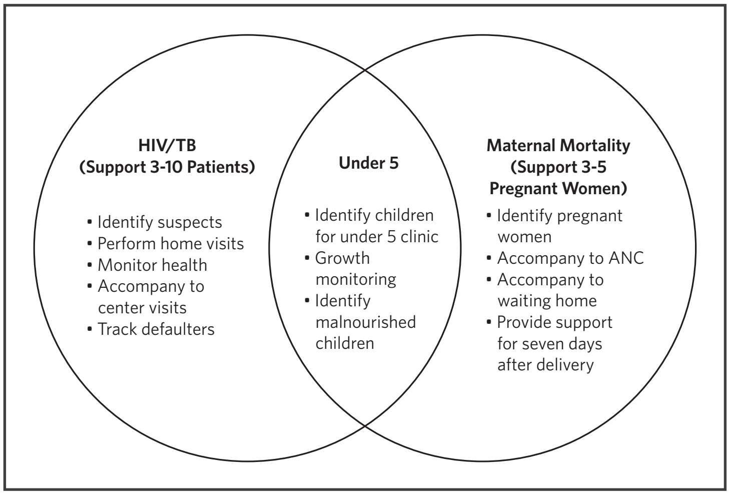

"We received training, this for me was a great success because we knew nothing. They also introduced supervisors, having supervisors is a great thing and a great success because we are many VHWs and if every VHW had to bring their own work to the health centre from their own village, it would be chaotic so having a supervisor is a great success."

VHW, Berea District

"VHWs have tools to report now with the help of the Reform, VHW report to supervisor, supervisors report to the coordinator and coordinators submits reports to the DHMT"

Mohale's Hoek District

Accompaniment

Prior to the reform, VHWs were not responsible for accompanying patients to facilities. Under the new guidelines launched by the National Health Reform, VHWs were now responsible to accompany their patients through communicating with, convincing, and escorting community members to access care at the health center. Patient accompaniment is associated with an increase in utilization of maternal health and HIV services. As shown in Figure 33, accompaniments by MMRPAs remained high throughout the National Health Reform period and are correlated with an increasing trend in the number of first ANC visit, fourth ANC visit, and PNC visits in the same period.

Figure 33: Maternal Accompaniments Compared to ANC and PNC Visits

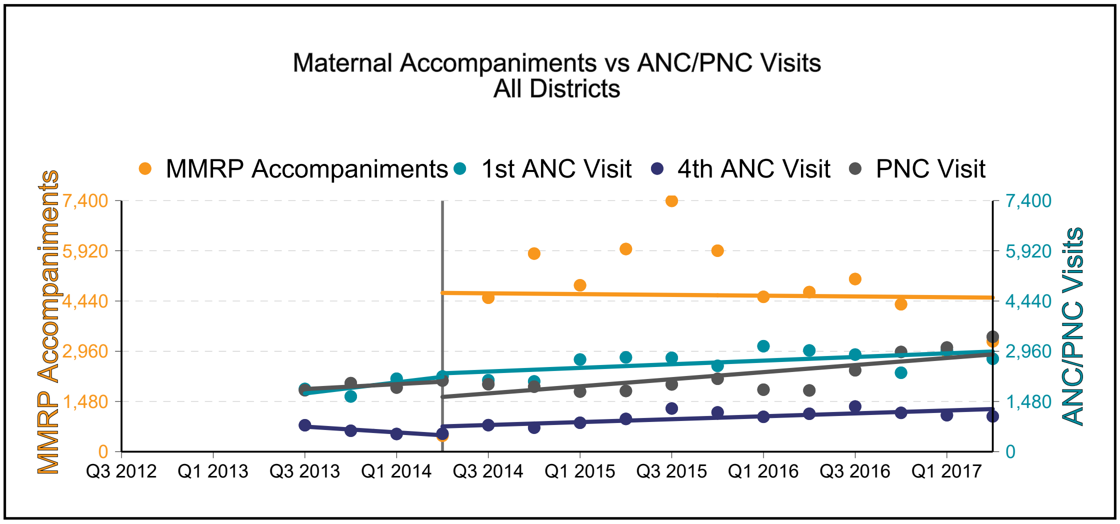

"Sometimes you may find that the person needs a little bit of a push. If he now feels that "at least I have someone to hold my hand, [and escort me to the health center]," it makes them feel valued somehow, and they [would be] more willing than when they were expected to come on their own. They [appreciate] the support and they were encouraged to do the right thing"

Butha-Buthe District

Defaulter Tracing

Key Results

— Prior to the National Health Reform, the proportion of patients enrolled on ART who were lost to follow-up (LTFU) was 27% across all health facilities. Following National Health Reform implementation, the proportion of ART patients LTFU declined to 22%, a 17% reduction ($p<0.05$).

Figure 34 depicts a reversal in the trend of HIV and TB patients who were lost to follow-up which were increasing at the baseline but showed a declining trend in the National Health Reform implementation period.[15] HIV/TB accompaniments remain high throughout the National Health Reform period with a slight decline in the later quarters.

Figure 34: Maternal Accompaniments and ANC/PNC Visits

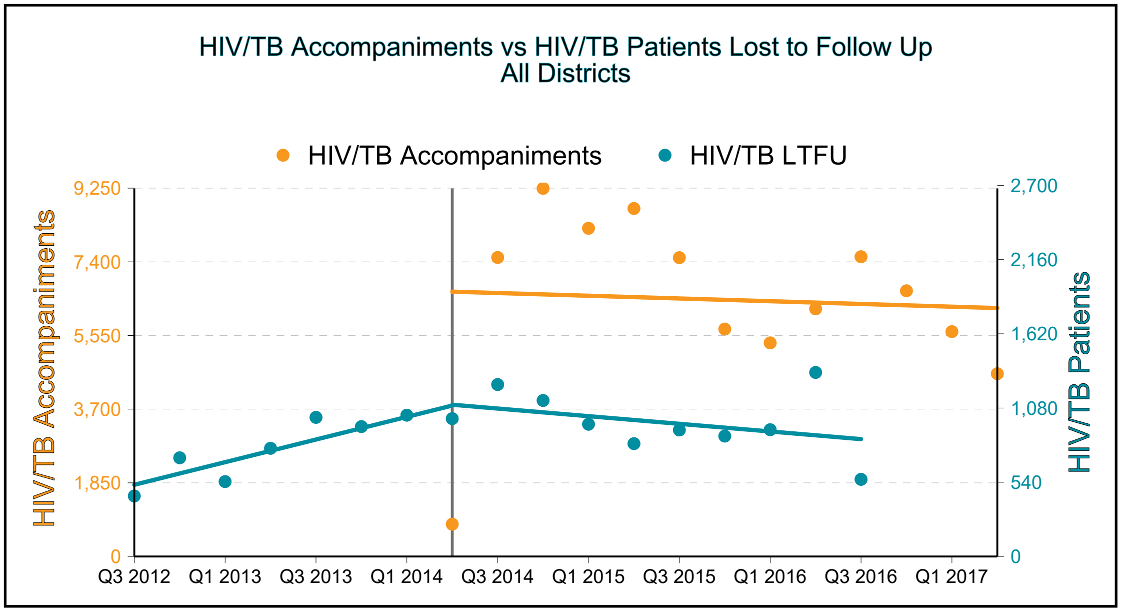

> *"Reform has done good because before it started, patients would be lost and no one would follow them up, after Reform VHWs [were] able to reach the community and to track patients"*
>
> *Berea District*

[15]LTFU is defined as patients who have not showed up for their appointments for more than 90 days for HIV patients and more than 60 days for TB patients.

Performance-Based Incentives

Performance-based incentive payments were designed and implemented to motivate and drive the performance of VHWs. Performance was evaluated on the VHWs adherence to assigned tasks including performing defaulter tracing, accompaniment of patients to the health facility, participation in meetings, and submission of periodic reports, with each task having equal weight. If a VHW performed all four tasks during a pay period, they would be paid the maximum rate of 400 Maloti. VHW payments were made using mobile banking as opposed to cash payments which made the payment process more efficient and timely.

> *"Before the Reform, they were paid [by] the Ministry, and people just got their money into their bank accounts without producing any reports, without proof of really working. But with the Reform, [payment was] performance-based"*
>
> *Berea District*

As shown in Figure 35, VHW meetings, one of the required tasks for incentives, increased over the National Health Reform period, showing more consistent meetings and engagement of the VHWs compared to interrupted and sporadic meetings observed at the baseline. VHW meetings were used for monitoring performance in the reporting period and providing CHW coordinator guidance to VHWs.

Figure 35: VHW Meetings Per Quarter

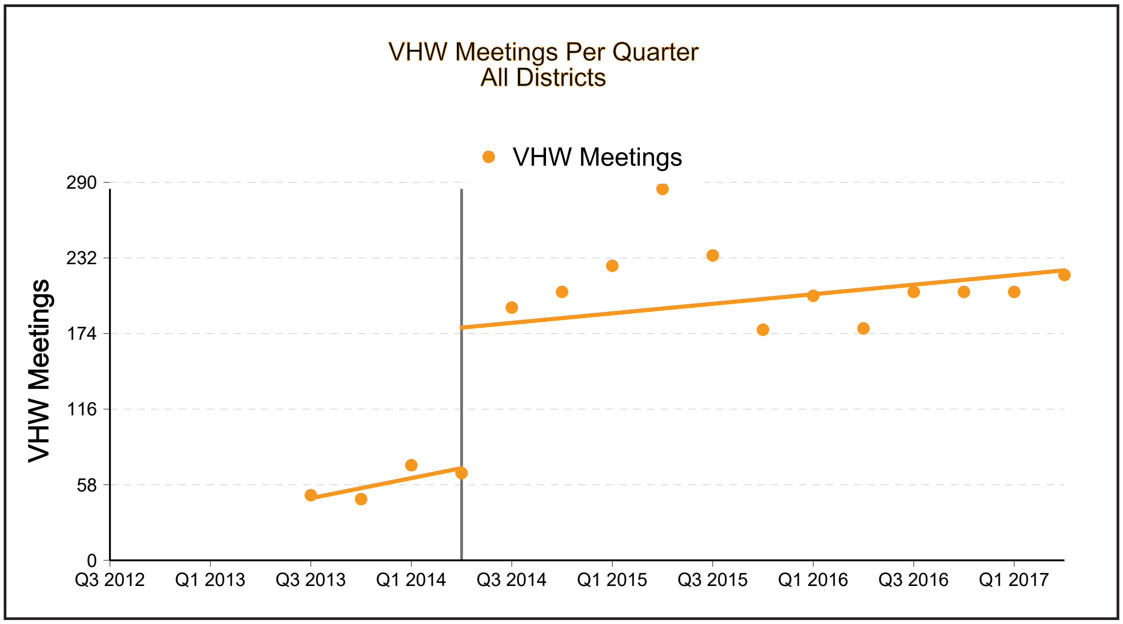

> *"Before Reform, we hardly see VHWs in the health facilities but nowadays, they make sure that every month they come to the facility. [....] during meetings with VHWs [we talk to them] more to keep on reviving them"*
>
> *Mohale's Hoek District*

HEALTH WORKFORCE

Key Results

— Staffing levels at DHMTs increased from 64% filled vacancies at the baseline to 89% following the National Health Reform.

— Throughout the National Health Reform, staffing at health centers increased in the following new staffing categories; data clerks, cooks, VHW coordinators, and pharmacy technicians.

— Following the National Health Reform, the average number of VHWs in the National Health Reform districts increased by 75%.

The National Health Reform introduced new positions critical for effective and efficient service delivery including: Pharmacy Technicians at all reform districts and high volume health centers, Data Clerks, VHW Coordinators, and Cooks based at maternal waiting homes. In addition, capacity building and skills trainings were conducted for a variety of staff and recommendations were made to the MoH and other partners to increase the number of Nurse Midwives and Counselors.

Figure 36: Staffing Levels at Health Centers Before and After Implementation of the National Health Reform

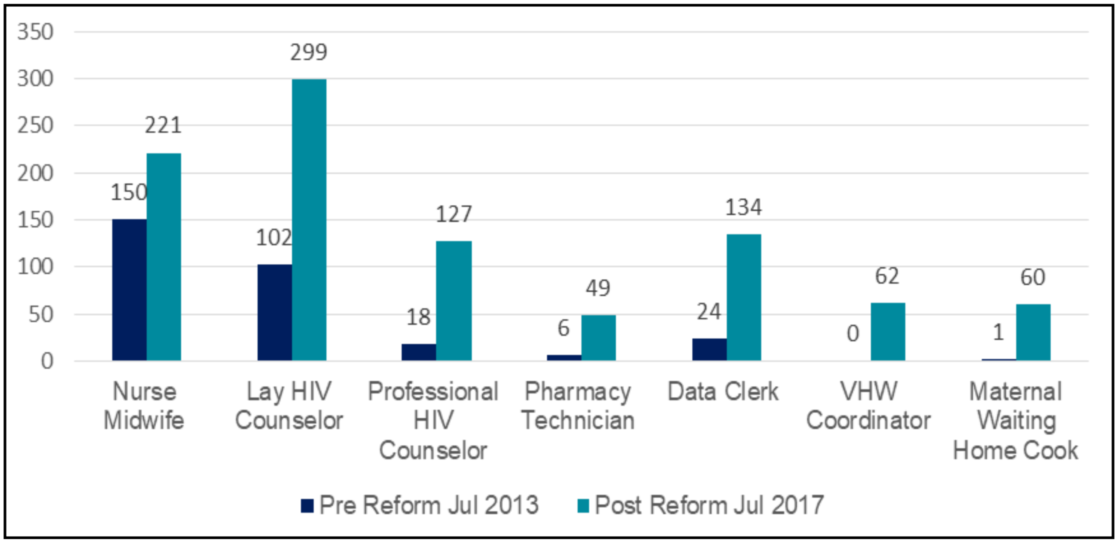

Figure 37: Number of VHWs Pre and Post National Health Reform

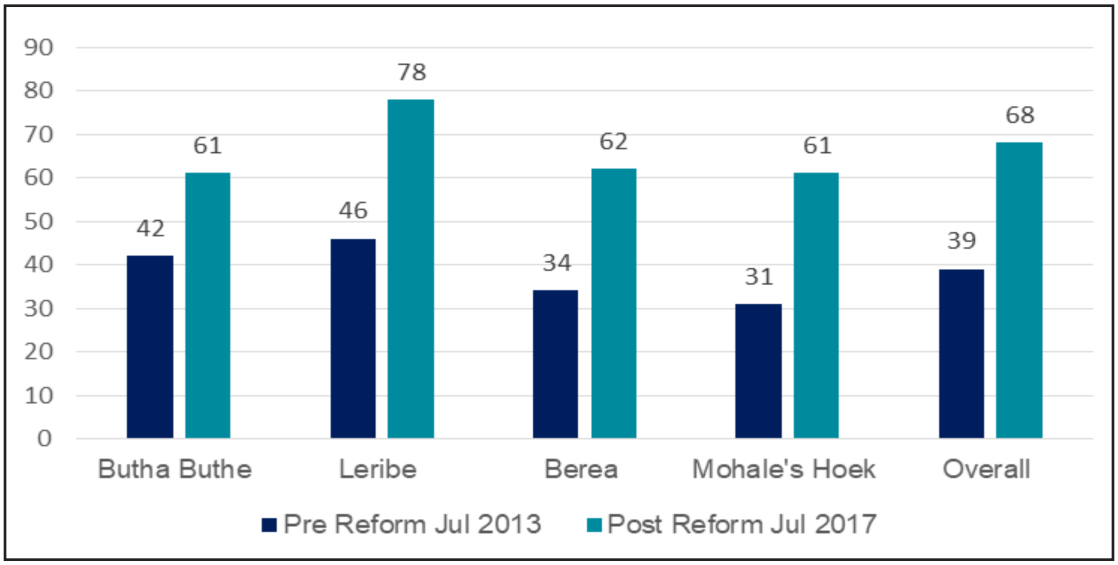

"Having a VHW coordinator to whom VHWs report to, [...] has really reduced the nurse in charge's work load because previously VHWs would report straight to the health center's nurse in charge. Now they report to[...] to VHWs supervisors, those supervisors report to the VHW coordinator, that flow of data did reduce the workload on the health center manager because previously they would report straight to the nurse in charge"

Berea District

MEDICAL PRODUCTS AND TECHNOLOGIES

Key Results

— During the National Health Reform, the availability of essential drugs, maternal and child health products, and ARVs were maintained at a level of 90% in Reform-supported districts.

The National Health Reform successfully introduced pharmacy personnel at the health center and district levels, established a system for supervision and oversight, and promoted optimal allocation of medical supplies within districts to improve availability of essential drugs and other medical products.

> *"Reform gave us the pharmacy personnel which was a relief. Everything was relying on the nurse; supply, ordering they were not even familiar with those steps on how to follow on ordering of the drugs and some drugs would [....] expire while nobody is checking them. So having pharmacists has been a relief"*
>
> *Berea District*

To fill critical knowledge and skills gaps in supply chain and drug administration, the National Health Reform introduced pharmacists at the district level and in high volume health centers. A total of 43 additional pharmacists were deployed to district hospitals and health centers, greatly enhancing the capacity and process for quantification, ordering, storage, and administration of essential drugs and medical supplies. In addition, a system for supervision and oversight of health centers by district pharmacy personnel was established, to provide accountability and guidance to health centers. Under this new structure, district pharmacists continuously communicate with health centers and conduct quarterly supervision visits to ensure availability of essential medicines and supplies.

> *"I think the Reform really helped through empowering the district pharmacists through their quarterly supervisions, they usually check the stock levels in the health centers. So really, we are not supposed to run short of the medications unless the medication is out of stock at NDSO. I think Reform [helped us] to know minimum stock levels, the required stock levels, and know when [we]have exceeded that"*
>
> *Mohale's Hoek District*

Through the National Health Reform, mechanisms were established to facilitate the optimal allocation and reallocation of essential medicines and supplies within health centers in the district. Pharmacists monitored the distribution and utilization of medicines and supplies and redistributed them according to need among health centers in the district. This optimal redistribution of resources maximized efficiency and avoided stock-outs.

> *"Redistribution of supplies among the different health centers, even the hospitals was brought by Reform. Pharmacist goes for supervision and he can see the shelf of one of the health center has got high number of drugs, [....] he then might pull those other drugs and give to the other health center which [needs them]"*
>
> *Leribe District*

FINANCING

The National Health Reform prioritized three key interventions to improve financing in the primary health care system:

- Removing financial barriers associated with emergency transportation
- Improving financial management capacity of DHMTs
- Prioritizing targeted investments

Removing Financial Barriers

One of the major barriers to achieving UHC is financial expenditures associated with health services. In Lesotho, primary health care services at the health center level are free with no user fees. However, patients are charged user fees when referred to hospitals. The National Health Reform established an emergency transport mechanism with local businesses to strengthen referral linkages between health centers and district hospitals. Furthermore, the system provided protection from the financial burden of accessing care by covering the cost of emergency transportation.

The cost of transportation for a patient from health centers to district hospitals can be significant; in many cases, the cost of transportation to the district hospital is likely to be higher than the cost of care. For example, a patient referred from Motete Health Center to Mamohau Hospital in Berea District for an emergency caesarean section would pay 900 Maloti ($62 USD) for transport and up to 300 Maloti ($20 USD) for the procedure and inpatient care. The estimated total cost of care might run up to 1,200 Maloti ($82 USD), transportation cost constituting 75% of the total cost of care. The total cost of care can therefore quickly add up to more than 1,000 Maloti or an equivalent to approximately $68 USD, with transportation constituting a sizable portion. In a country where more than half of the population live on less than $2 USD per day, this can create a catastrophic health expenditure for rural households.[16] The emergency referral system established as a component of the National Health Reform removed a significant portion of the total cost of care.

> *"I remember when I was in Mohale's Hoek, in one of the villages, Mapotseng, patients had to walk close to four hours to the health center. Through the village health worker, we got to know that there was a lady who was pregnant, around 21 of age, and [she was] somebody who was born disabled, who had never walked. That person wouldn't be able to deliver at home or in the health center. But through the referral transportation, we were able to assist that patient up to the hospital to deliver in the hospital, through Cesarean section, and then back. And also, the village health worker made sure that the child was still going for immunizations at the health center"*
>
> *Mohale's Hoek District*

Improving Financial Management Capacity of Districts

The National Health Reform built the capacity of DHMTs to manage their budget. There is an improvement in planning, appropriately allocating budget for the prioritized activities, and better utilization of allocated budget in the National Health Reform districts.

[16] World Bank (2018)

"We plan to use left over money from the DHMT top-ups effectively, sometimes we say, 'okay, let's look at the district, what can we buy or improve?' For example, we have an ambulance that it is not working, let's go and service it."

Leribe District

Prioritized and Targeted Investments

The National Health Reform made targeted and prioritized investments to maximize value for money and tackle key inefficiencies. The National Health Reform started allocating resources to training, supervision, and mentorship, funding critical human resources, paying incentives to drive performance, enabling supplies such as sterile delivery packs, and referring emergencies.

The total investment by the National Health Reform in the four target districts was relatively modest considering the results achieved. The total cost over the four-year period was about $7 million USD amounting to an average of $1.5 USD per capita (see Figure 38). It is important to note that this analysis is limited to spending at the district level; the investment does not capture human resources and administrative investments from PIH.

Figure 38: National Health Reform Spending Per Person

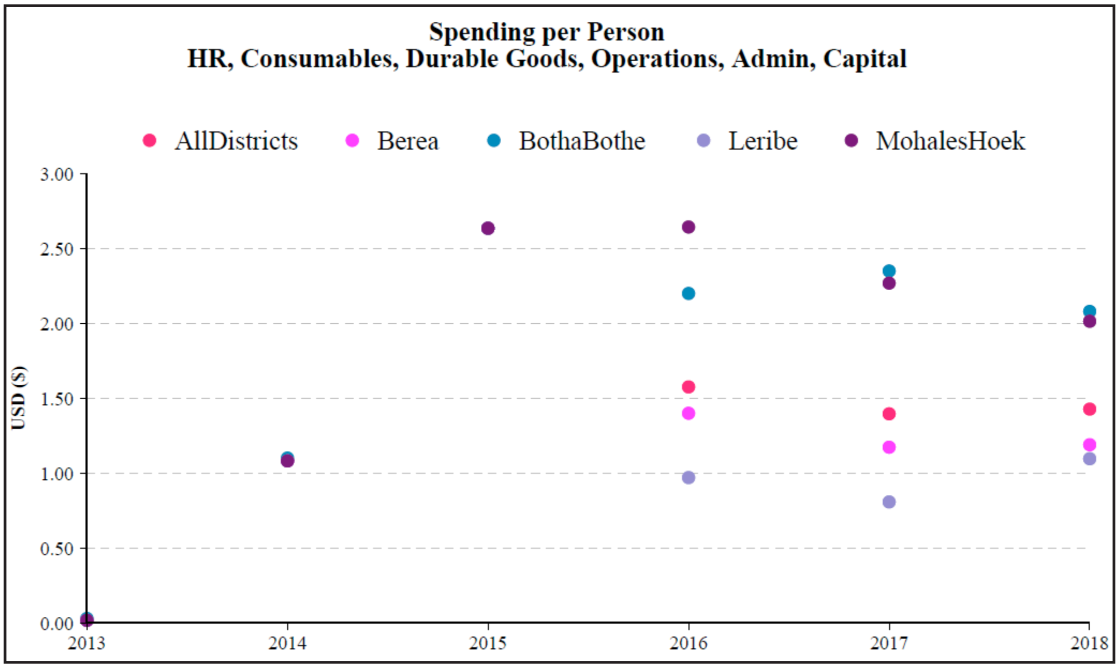

SYSTEMS FOR DATA COLLECTION AND MANAGEMENT

Key Results

— Information systems improved dramatically, with data availability increasing from 85% to 99% between 2013 and 2017.
— Data availability specific to the VHW program saw the most impressive improvement, from 1% before the National Health Reform to 74% during the National Health Reform.

The National Health Reform employed the following strategies to improve systems for data collection and management at the health facility and district levels.

- Implemented a set of tools, including registers and reporting formats, to ensure that data capturing is uninterrupted.
- Created standards for report flow, review and feedback.
- Established systems for accountability for the management, supervision, and oversight of information systems.

To strengthen data collection and management systems at the community level, a system for accountability was established with VHW coordinators and supervisors to review and provide feedback to reports. Simple, user-friendly reporting formats were developed to ensure reports were easy to use and VHWs were trained on how to complete reports, with ongoing mentorship built in to the VHW supervisor structure. Lastly, accurate and timely reporting was one of the VHW incentive performance criteria. The combination of these interventions has dramatically improved data availability and use at the community level.

> *"After reform, we worked to make sure that all monthly reports from the health centers are on time and health centers submit the monthly report to the DHMT directly, by the first week of the month. [..]over two weeks following submission, M&E, HMIS officer look into the data and verify and address those gaps, and then HMIS officer, presents the data to DHMTs"*
>
> *Butha-Buthe District*

In addition, a system for quarterly reviews at the health center and district levels was established, promoting consistent data review and use. It also created a sense of collaboration among health centers and provided an opportunity for sharing experiences, lessons learned, and best practices between facilities and practitioners.

"In terms of reporting and also accountability at the district level, we have now quarterly review meetings, whereby the program managers present to the whole team what they have been doing for the quarter. So it gives us an opportunity to learn and share from each other and also advise different programs"

Mohale's Hoek District

Support was also provided in data analysis and interpretation of results to districts and health centers to promote evidence-based decision-making in program implementation, management and planning.

"The Reform helped us analyze our own data properly. Data should be meaningful to its users, before it was just the numbers, but with the Reform, it has now started making sense, before it was just numbers"

Butha-Buthe District

LEADERSHIP AND GOVERNANCE

The National Health Reform introduced transformational ideas to strengthen leadership and governance in the National Health Reform districts. The key interventions included:

- Reorganized DHMT structure to promote accountability and consolidate governance to better manage primary health care.
- Established a process for planning, budgeting, and target setting at the district and health facility levels.
- Created a mechanism for periodic performance reviews.
- Implemented a system for performance-based incentives for DHMTs.

Reorganization of the DHMT Structure and Governance

To create an accountable leadership structure for the district, DHMTs were reorganized to have a District Health Manager oversee all health programs in the district. The National Health Reform also clearly defined roles and responsibilities of the DHMT. Reorganization aimed to empower the team to make decisions on resources and effectively manage health care delivery in their district. Similar reorganizations were implemented at the health center level.

“Roles were not defined, there was no proper leadership in the district or not well-defined leadership structures in the district and people did not know their roles [...]. So after the Reform started [this]was cleared, people knew what to do in the district”

Butha-Buthe District

“With the Reform, the DHMT and the district health manager were overseeing the entire district. Hence it was our responsibility to share the resources within the district. If one facility – or if anything comes to the DHMT and you find that the need is more in a CHAL facility, you don’t say the DHMT gets funding from government and CHAL gets its funding through the subvention. You just have to see how you will close the gaps”

Berea District

The National Health Reform promoted decentralized decision-making and consolidated management structures at the district level so that DHMTs can manage health services effectively. With health centers and hospitals under the direct management of DHMTs, efficiencies were realized in sharing of resources, accountability, and integration between heath center and hospital care.

“Before Reform, the hospital and the DHMT were working in isolation, so that means there were two Ministry of Health in the district. Because the DHMT will only focus on the health centers, they will say “this is our transport.” And the hospital will also say “this is our transport.” [.....]. But the Reform, it joined the whole team to be under one umbrella we joined everything, even the transport, we gave it to one person to monitor the transport for the district”

Mohale’s Hoek District

Planning and Target Setting

Capacitated DHMTs were able to map the burden of disease and set UHC targets for HIV, TB, maternal and child health, and other priority disease areas based on the size of the catchment population of each health center. Operational plans and budgets were then developed to meet UHC targets. Planning and target setting processes helped DHMTs and health facilities appropriately quantify their needs and monitor progress against targets.

"We define the targets for the health centers. Your catchment population is this much, and based on your catchment population and the district burden – disease burdens, you are supposed to see this much of HIV patients, this much of TB patients, this much of the ANC care"

Butha-Buthe District

Periodic Performance Reviews

The National Health Reform initiated monthly intra-district review meetings including district hospitals and quarterly review meetings between districts and central stakeholders. These periodic reviews are used to monitor progress toward targets and make programmatic adaptations in order to continue to meet the needs of the population. `

The period reviews were also used in some districts to motivate performance and create an atmosphere of healthy competition among health centers in a district. Most improved health centers were recognized, which motivated other health centers to follow suit.

"We started identify the best performing health center and I think the implementing partners supported those prizes, people are getting recognized for different trophies that were revolving, says this time you were the best performing health center on the maternal health issues. The next time, the other health center would like to fight for that trophy, to bring that trophy to their health center"

Butha-Buthe District

Performance-Based Incentives for DHMTs

The National Health Reform introduced performance-based incentive payments for the management team of DHMTs. Broad performance criteria were established, but decisions on incentive payments were up to the discretion of the districts. Districts used incentive payments for salary top-ups of the management teams based on performance and made decisions on how to use the left over funds for priority activities. Most often, districts used leftover funds to maintain vehicles for supervision or other immediate needs.

RECOMMENDATIONS

Several lessons can be taken from Phase I of the National Health Reform implementation. This section presents policy recommendations based on lessons learned to further strengthen the National Health Reform and advance progress towards UHC.

1. Empower DHMTs to manage financial and human resources

Strengthening DHMTs and shifting decision-making for budget and management to the DHMT level has immensely improved performance of DHMTs, and is well aligned with Lesotho's policy of decentralization. With the National Health Reform, DHMTs were empowered to plan and coordinate primary health care including decision-making on optimal distribution of supplies across the district. Many examples have been documented demonstrating how DHMTs redistributed medical supplies within their district, resolving supply deficiencies and promoting efficient use of resources. However, DHMTs are not empowered to make decisions about financial resources or human resources. For example, DHMTs are not able to recruit for job openings, which is done centrally by the MoH. This reportedly delays recruitment and increases the chance of recruiting unsuitable candidates for the district.

Financial and human resources are very important components of managing a district health system and hence empowering DHMTs to make decisions about optimal allocation and use of financial and human resources is crucial. This approach will promote efficiency and problem solving at the facility and district levels. It is therefore recommended that the GoL and the MoH explore the legal and operational requirements for more influence of DHMTs on financial and human resource matters, simultaneously delineating the roles and responsibilities of the DHMTs and MoH. This should be done under the leadership of the MoH and aligned with efforts for decentralization in close collaboration with other sectors and ministries.

It is also crucial that as DHMTs are empowered to take more responsibility of district resources, they have the capacity and systems in place to effectively manage them. Establishing guidelines for financial and human resource management coupled with training, mentoring, and supervision from the MoH and the local government are key to improving management and performance at the district level.

2. Continue to build management and leadership capacity

The National Health Reform promoted decentralized decision-making and empowered DHMTs to control the management of primary health care in their districts. Efforts to give more management responsibilities need to go hand in hand with the building of management capacity. To address this, the National Health Reform has provided management and team building trainings. Such efforts need to be sustained and institutionalized so that DHMT and health center managers are provided with the management tools they need to effectively carry out their responsibilities. The following approaches for building management and leadership capacity should be considered.

1) **Management and Leadership Courses:** Didactic management and leadership courses which include the reading and understanding of leadership literature, self-reflection, diagnosis of external environment, and analysis of real-life application of management and leadership concepts.

2) **Practical Problem Solving:** Teaching managers problem solving approaches and techniques and creating the opportunity for them to apply what they have learned in real-life settings through practical projects in their district and health facilities.

3) **Coaching and Mentorship:** Identifying management mentors and coaches within and outside of the health sector and pairing them with DHMT and health facility managers for professional and personal development to help managers overcome a specific issue or performance challenge.

4) **Shadowing:** Shadowing is a technique in which a person in a leadership development program accompanies, observes, and collaborates with a senior leader while they exercise leadership functions. DHMT leaders could be paired with leaders at the MoH, creating an opportunity for participants to observe in-action leadership and management practices.

3. Enhance accountability using management standards and community scorecard

The National Health Reform has demonstrated the value of accountability at all levels of the health system. The introduction of supervisors and coordinators for VHWs, reorganization, and clearly defined job descriptions at the DHMT level has created the opportunity for task performance to be subject to oversight, direction, and information systems strengthening for accountability. This in turn has contributed to improvements in services presented previously in this report. These improvements can be further enhanced by introducing management standards and a system for community accountability.

Management Standards

Evidence shows that better managed districts and health facilities are more likely to provide quality health care and coverage of health services to the population.[17] It is therefore important to measure, monitor, and continuously improve management performance. The data captured during the National Health Reform did not effectively measure management performance; as a proxy, management performance was measured by the number of meetings and supervisions, providing limited insight to the impact of management performance. Alternatively, management performance could be measured by objectively benchmarked standards against organizational performance. Management standards, which measure management tasks and processes, could be effectively employed to hold health managers at the health centers and DHMTs accountable. Furthermore, management standards will contribute to the standardization of service provision across facilities managed by different entities, including the GoL and CHAL.

Management standards at the health facility level should focus on how health facilities are managed, including the management of assets, human resources, finances, and the day to day management of the facility, including decisions about patient flow, medical equipment and stock management. At the district level, management practices should be concerned with community engagement, service delivery, performance management, and engagement with other sectors. District management standards could be identified and structured using the WHO health systems building blocks. Such management standards at the health facility and district levels break down management functions into quantifiable and measureable tasks enabling management accountability in the health system. If used to set targets for management performance with periodical assessment, management standards could be an effective tool to ensure that changes brought about by the Reform are sustained and further enhanced.

The most effective way to implement management standards is to create accountability at the local government level where District Administrators are empowered to monitor, support, and make DHMTs and health facilities accountable. Management standards can also be a powerful accountability tool for the MoH to hold DHMTs and health facilities accountable and can be linked with incentives to drive performance.

Community Accountability

In addition to standards and systems within the health system structure, community level ratings can further develop accountability by enabling the users of these public services to voice their needs and create opportunities to hold service providers accountable for the performance of the health system. Such systems can be integrated with the VHW program and Health Center Committees to make it more responsive to the needs of the community. One such tool is a community score card, which can be used by community

[17] Fetene N, Canavan ME, Megentta A, Linnander E, Tan AX, Nadew K, et al. (2019) District-level health management and health system performance. PLoS ONE 14(2): e0210624. https://doi.org/10.1371/journal.pone.0210624

members to evaluate access and quality of health services they receive using community developed indicators. Introducing a community score card can add value to already existing community engagement mechanisms such as the Health Center Committee, by providing a quantifiable and actionable data on community perceptions essential to create transparency and accountability between primary health care facilities and the community they serve.

4. Use of Key Performance Indicators (KPIs) for performance management

Performance management has greatly improved in the National Health Reform districts with the introduction of tools to measure progress toward UHC targets and a mechanism for reviewing performance using periodic district review meetings. In addition, a decentralized planning process has been established at the district level. These encouraging developments could be further strengthened by introducing key performance indicators (KPIs) to measure primary health care performance across districts.

An optimal combination of management and service delivery indicators which reflect key priorities for primary health care in Lesotho (agreed upon by relevant stakeholders) could help focus performance management efforts. This could be integrated with DHIS2 systems where KPI dashboards could be introduced at all levels to help managers monitor performance of programs toward UHC targets. KPI measures could also be used to rank high and low performing health facilities and districts and link performance with incentive and accountability mechanisms. This could provide an understanding of the drivers of good performance and a root cause analysis for lower performance. Use of KPIs can also promote improved data quality and use. KPIs are used in the private and public sectors to drive performance and its use in the health sector in Lesotho could greatly enhance performance management at all levels.

5. Develop guidance and standards for mentoring and supervision

The mentoring and supervision support DHMTs provide to health centers has been very helpful to health centers. After the National Health Reform, mentoring and supervision efforts have become more focused and targeted. This could be further improved by delineating the focus areas for supervision and mentoring, as they serve different purposes and have different approaches. This can be done by providing detailed guidance for the mentoring and supervision relationships between the MoH and DHMTs, DHMTs and Hospitals, DHMTs and health centers, and hospitals and health centers.

There could also be more structured approaches for mentorship of health care workers at the health center level by including standards for one-on-one case discussions, chart reviews, clinical audits, and feedback from referrals intended for building knowledge and skills and improving quality of service.

It is also crucial to measure the effectiveness of mentorship by conducting periodic knowledge and skill assessments of health care workers, particularly at the health center level. This mentoring process can help identify capacity gaps that could be the basis for training of health care workers and also attachment in high patient volume health facilities for building skills. In order to achieve this, coordination with hospitals and other facilities, setting minimum criteria for managing specific clinical procedures by health center nurses, administering robust knowledge, and skills assessments periodically is vital.

6. Harmonize incentive mechanisms

The National Health Reform has demonstrated the value of using incentives to drive performance in both the VHW program and the DHMTs. Despite the proven value of linking performance with incentives, they can also be a source of frustration if applied inconsistently. It is therefore crucial to ensure that incentive mechanisms are consistent across districts and are effectively supervised and verified over time. Incentives must be linked with clear and transparent performance criteria at all levels of the system, including the

community, health facility, and district levels. Applying performance-based incentives in an inconsistent manner across different levels of the health system and in various districts will be a source of frustration and dissatisfaction. It is therefore important to develop a national policy on performance-based incentives that can harmonize incentive mechanisms, including those implemented by various partners, and clearly define expected performance detailing how, when, and in what frequency incentives should be applied to ensure consistency and equity.

7. Strengthen community program though formalizing VHWs and establishing Community Health Information Systems

Through the National Health Reform, accountability, supervision, oversight, and performance-based incentives has transformed community engagement and elevated the effectiveness of VHWs and the community health program. This could be further enhanced by sustaining current reforms and establishing a Community Health Information System (CHIS) for collecting and analyzing household level data that captures the health profile of households and vital events at the community level.

Sustaining VHW Program Reforms

The evaluation of the National Health Reform and various reports from the field confirm that payment of VHWs has been interrupted following the transition of payment responsibilities from PIH to the MoH, which negatively impacted the VHW program and became a source of frustration for VHWs and health workers at the district and health facility levels.

Political commitment and leadership of the MoH and the GoL is required to address this by establishing policy that formalizes VHWs and their performance-based compensation. Currently, VHWs have volunteer status and are not formally recognized as employees, which creates a challenge for appropriately compensating them. Trained and preferably salaried community health workers integrated with the health system are the backbones of effective primary health care service delivery. Lesotho needs to seize the opportunity of its existing VHW program for advancing UHC.

Community Health Information Systems

The key goal of the VHW program is to reach community members in need of services and ensure access to care. Such efforts could be greatly aided by a system for tracking household level data in a village. Establishing a CHIS could further transform the VHW program and promote the use of data at the community level for more effective, efficient, and targeted community level interventions. CHIS could be designed to capture key information on the health status and health service provision in the community. This information could be vital to VHW supervisors, VHW coordinators, health center mangers, and DHMTs. CHIS could further strengthen the VHW program reform by promoting community engagement, better identification of people in need of services, support referral and retention in care, and accountability of VHWs.

8. Update establishment list and optimize human resources allocations based on demand

One of the approaches promoted by the National Health Reforms is mapping diseases against resources to ensure efficiency and optimal use of resources. One of the most critical and scarce resources in Lesotho for health care is human resources. To maximize the effective use of available human resources, the MoH should optimize allocation of existing and future deployment of health care workers to align with demand for services, determined by patient volume and work load. Therefore, the MoH should consider conducting a demand-based human resources optimization analysis to inform policy on human resources allocation. The objective of the optimization analysis would be to determine the optimal number and allocation of health care workers to adequately meet demand for services at national, district, and facility levels.

In addition, the reforms implemented should be fully recognized and integrated with national policies and procedures to ensure their sustainability. In this regard, it is important for the revised organizational structures, newly created positions, reformulated job descriptions, and reformulated management structures at the DHMT and health center levels to be fully recognized and integrated in the establishments list, human resource systems, and processes.

9. Update Essential Package of Health Services (EPHS) to be aligned with UHC

The Essential Package of Health Services (EPHS) is a package of services that governments provide, or aspire to provide, to their citizens in an equitable manner. Lesotho's EPHS was developed in 2005 and has not been updated since. As service utilization increases, the epidemic profile of countries changes and the aspirations and standards of health services evolve, countries need to update their EPHS.

The EPHS should be updated in order to make it relevant for a dynamic epidemiological landscape aligned with UHC and the Sustainable Development Goals (SDGs).

The National Health Reform Evaluation has recommended the decentralization of basic diagnostic laboratory services, including TB diagnosis and other lab tests for pregnant women, from hospitals to health centers. This shift could be considered in the context of reviewing the EPHS and establishing the type of services to be provided at the primary health care (PHC) level including associated lab capacities to effectively respond to the needs of populations served.

The inclusion of priority services in EPHS does not necessarily mean that they will actually be provided to the people who need them. In addition to including essential PHC services in the EPHS, there is a need for service delivery to be adequately resourced with people, materials, finance, and infrastructure to ensure initiation and continuity of services.

10. Strengthen leadership at the MOH level to sustain and scale the National Health Reform through coordination and alignment

One of the key issues identified through the evaluation of the National Health Reform is the MoH's central roles for the success and sustainability of the National Health Reform efforts. The MoH should continue to play an important role by coordinating and aligning all stakeholders including funders, implementers, district administrations and other sectors to one national strategy around building a strong and decentralized primary health care system lead by empowered DHMTs to accelerate progress towards achieving UHC. The leadership of the MoH is also vital to consider and implement the policy recommendations set forth in this report, so that the gains made by the National Health Reform will be scale up to all districts, sustained and expanded upon.

CONCLUSION

Due to Lesotho's high burden of communicable diseases, including HIV and TB, the health sector's response, particularly in the primary health care system, was dominated by vertical programs funded by various Global Health Initiatives. However, this selective primary health care approach did not result in an overall improvement of the health system nor did it reverse the burden of communicable diseases. A health systems assessment commission by the GoL in 2013 determined that the primary health care system had weak managerial capacity at the district level and failed to provide quality services to the population, resulting in very low utilization of services for HIV, TB, maternal and child health, and other disease areas.

The National Health Reform demonstrated that relatively modest investments targeted at health systems to improve comprehensive primary health care service delivery are beneficial in the short term for addressing current priorities and in the long term for preparing the health system to address emerging priorities. Promoting local decision-making by empowering the districts to lead primary health care and supporting all of the building blocks of the health system address inequalities in access to health care and makes PHC an effective tool for advancing UHC.

There have been significant improvements in health service utilization for maternal and child health and HIV services as a result of the National Health Reform. Even with these improvements there is still a long way to go until Lesotho is able to achieve UHC targets. The PHC movement has been revived by the Global Conference on Primary Health Care held in Astana, Kazakhstan in October 2018. The conference resulted in a new declaration, emphasizing the critical role of primary health care around the world. The Government of Lesotho demonstrated its support of this movement by becoming a signatory of the declaration. The conference declared that PHC is the foundation to achieving UHC and the health-related Sustainable Development Goals (SDGs). There is a need to capitalize on this renewed global movement for comprehensive primary health care, district health systems strengthening, and UHC to mobilize resources and expand Reform efforts across all districts in Lesotho. Taking this declaration into action, the GoL and MoH can lead the way by advancing a PHC policy that promotes the National Health Reform agenda and coordinating all relevant stakeholders to coalesce and streamline existing and new funding towards effective primary health care investments.

ACKNOWLEDGEMENTS

Partners In Health Lesotho would like to express our deep gratitude to the Ministry of Health and the District Health Management Teams of Leribe, Berea, Mohale's Hoek and Butha-Buthe Districts, for their enthusiastic participation in the implementation and evaluation of the National Health Reform.

We would like to thank the Analysis Group who analysis support for this report. Our grateful thanks are also extended to Abraham Zerihun Megentta who served as a consultant leading the drafting of this report.

We would also like to extend our thanks to the Partners In Health team for coordinating this report and providing invaluable comments and insights.

Finally, we wish to thank Ms. Mathabang Mokoena who led the production of this report and all team members of the Partners In Health Lesotho team who were involved in the production of this report.

Partners In Health

800 Boylston Street, Suite 300
Boston, MA 02199
857-880-5100
www.pih.org

Partners In Health is a 501(c)(3) nonprofit corporation and a Massachusetts public charity.
© 2020 Partners In Health. All rights reserved.
