# Supplementary material for: Improving access to health services through health reform in Lesotho: Progress made towards achieving Universal Health Coverage
Source: PLOS Glob Public Health. 2022 Nov 16;2(11):e0000985. doi: 10.1371/journal.pgph.0000985 (PMC10021396; doi:10.1371/journal.pgph.0000985)
Supplement: S2 Text — It further includes a table demonstrating the three core areas identified as needs during the National Health Reform: Service Delivery, Health System Managerial Capacity, and the Professional Village Health Worker Program, and lists specific areas for targeted for improvement within each area. Those areas became the focus of the interventions within the National Health Reform. (DOCX) [file pgph.0000985.s003.docx]

**Appendix 1: Detailed Report of Interventions Included in the National Health Reform, Lesotho 2014-2017**

Through the baseline auditing and consultation, PIH and the MOHSW identified three inter-related deficits, which, if improved might translate to improved access to and quality of care delivered. First, the provision of services did not match the disease burden. Rather, massive gaps in staff, essential medicines and equipment, and inadequate space (particularly of labor and delivery beds) in facilities were pervasive. Most clinics in the face of those supply-side challenges saw very few patients per day. Second, district managerial capacity had been highly fragmented and disproportionately focused on vertical interventions (primarily HIV) and on a World Bank funded public-private tertiary hospital (1). Thus, little money from the national budget was sent to or controlled by the districts. Finally, the Village Health Program – a legacy of the Alma Ata conference (2) – was untethered to the health system and depended on the poorly paid, unsupervised labor of village health workers; used for a variety of one-off interventions (3).

The National Health Reform was developed in response to those factors and implemented in 2014 integrating service delivery at facilities, district health system management, and community health work (see Supplementary Table 1 below). First, supply-side reforms were made aligning inputs of the staff, supplies and space to match the burden of disease for the catchment area of each clinic. Secondly, district health teams were strengthened through decentralization of funds and the mandate to oversee health service delivery in the districts, coordinate supervisions and non-governmental organizations’ work to ensure such work aligned with their priorities and impact the district health targets. Third, the Village Health Worker program was strengthened with specific focus on HIV, Tuberculosis, and maternal and child health. Village health workers were trained and managed regularly by the health center against the target population of their village. The programs were then implemented the four pilot districts. The Harvard Human Research Protection Program granted ethical approval for this study (IRB17-19888), as did the Lesotho National Health Research Ethics Committee (id 117-2017).

*Service Delivery*

At the facility level, the DHS data for each district was compared with the catchment area to set targets for service delivery using the PIH Universal Health Coverage planning tool (4, 5). Based on that assessment inputs to the system were aligned with the expected need. For example, maternal mortality in Lesotho was among the highest in the world with local health facilities often unable to refer and transfer patients who required higher levels of care given the absence of emergency transportation services. Transportation costs in general contribute to a significant financial burden for individuals seeking medical care in Lesotho (6). And 44% of expectant mothers reported living more than a two-hour walk (many reporting one to two days walk) from the nearest Health Center—which often did not have full-time midwifery coverage.

To set up targets for antenatal care and facility-based deliveries, the number of pregnant women in a catchment area was calculated by multiplying the crude birth rate by the catchment area. Inputs were then aligned to assure that universal coverage (i.e., 100% of women delivering in the facility) could be achieved. Based on the expected number of pregnant women per year (7), the daily bed utilization was estimated and delivery tables were set up accordingly. In addition, the number of midwives needed to provide 24-hour coverage were hired and deployed proportionate to the population. Facilities were also supplied with single-use disposable delivery packs proportionate to the estimated number of deliveries to reduce infectious complications of reusing supplies given the absence of autoclave machines. The packs included disposable latex gloves, drapes, umbilical cord clamps, disposable scalpels, gauze sponges, and items required for sterilization.

Maternal waiting homes were established in 72 facilities based on the estimated number of women who lived more than two hours away by foot and the number of women who needed pre-labor evaluation. The facilities, which had sufficient beds to accommodate nearly 50% of women for one week prior to delivery, were staffed with cooks and supplied with food, heating systems, linen to ensure well-being of the mothers at the waiting homes. A new obstetric emergency referral platform was established by utilizing local business’ cars to transport mothers with complications from health centers to hospitals.

Morbidity-based mapping was used to assure improved supply chain management and reduce stock outs of essential medicines. The National Health Reform introduced 13 pharmacists— four pharmacists were deployed among district health management teams, while the rest were deployed proportionately to high-volume health centers. Pharmacists and managers were trained in stock management and systems were developed to improve communication between health centers and the district managers. Three pharmacy supply chain offices provided technical assistance to 70 health facilities to identify gaps and plan interventions. The team used 23 selected tracer commodities, 14 of which were global core listed medicines included in World Health Organization and Health Action International surveys (8), to continuously monitor availability and rate of stock outs of individual products. The team also measured capacity to calculate resupply quantity, ensured orders were submitted on time, and storage practices were adherent to national standards. In addition to human resources, cold chain capacity of five health centers was augmented to appropriately store several life-saving medicines (9).

*Health Systems Management*

Decentralization of resources and personnel is a viral part of health systems strengthening, and is dependent upon the development of managerial capacity (10). Improved health center management (11-13), and specifically improved district-level managerial capacity in resource-limited settings is associated with improved health outcomes (14, 15). Concomitant assurance of accountability is essential (16, 17). One tool to foster accountability is known as the community score card, which is a community lead governance tool developed to promote a collaborative assessment and prioritization of health issues between healthcare facilities, local government structures and the community. The community score card has been shown to improve citizen empowerment, service provider effectiveness, service provision and accountability as well as responsiveness (18, 19).

Thus, the National Health Reform aimed to decentralize both funding and decision making for district health systems to improve district-level managerial capacity with simultaneous implementation of the community score card and performance-based incentivization to ensure accountability. A District Health Management Team was established within each district, consisting of 16 members: a district public health nurse, a district inspector, a district information officer, a district. Health administrator, a district lab manager, a district accountant, a district hospital manager, a district hospital superintendent, a district human resource officer, a district TB coordinator, a district counselor, a district immunization officer, a district sexual and reproductive health mentor, a district nutritionist, and a district inspector, all lead by a district medical officer. The District Health Management Teams were responsible for overseeing health services delivery.

Management and leadership courses, training sessions in practical problem solving, mentorship and coaching, shadowing experiences, and psychometric assessments were all implemented across the districts included. Checklists for focused mentorship and supervision visits were developed and implemented, covering a range of topics inclusive of: maternal and child health, HIV, Tuberculosis, and non-communicable diseases. Clinical mentorship, in particular, was emphasized with one-on-one case discussions, chart reviews, clinical audits, and feedback from referral centers. Nurses were trained on labor monitoring and regularly mentored on integrated primary health care services by PIH and MOHSW joint teams. Similarly, efforts were made to decentralize basic diagnostic infrastructure such as laboratory services to improve local diagnostic capacity.

*Professional Village Health Workers*

The various roles and benefits of the community health worker model in resource-limited settings are well characterized (20-23). The Lesotho MOHSW, supported by PIH recruited, trained and deployed professional village health workers as a fundamental component of the National Health Reform. At the community level, 5,359 village health workers were recruited to increase coverage in all villages within the catchment areas. Training consisted of the standard PIH community health worker curriculum (24), which included topics such as disease prevention, accompaniment, and health seeking, as well as an additional seven days focused on topics of maternal health as has been previously reported (25). Furthermore, new roles were established consisting of village health worker supervisors and village health worker coordinators to ensure accountability of the village health worker program. The responsibilities of the village health workers included 1) community education on preventable diseases and health seeking through community gatherings, 2) door-to-door diseases surveillance, 3) accompaniment of patients to health visits, and 4) encouragement of adherence to prescribed treatments and retention in care. At each health center, committees were established to facilitate communication and engagement with the community and traditional leaders.

A performance-based payment structure was used to promote longitudinal accompaniment of women through antenatal care visits, facility-based deliveries and post-partum follow-up visits. Such incentives included patient accompaniment to healthcare visit, tracking of defaulters or individuals who were lost to follow-up, attendance at monthly village health worker meetings, as well as accurate and timely reporting of community data. The amount paid varied from 0-300 Rand for village health workers and from 0-500 Maluti for village health worker supervisors.

**Supplementary Table 1: National Health Reform Strategies**

| **Service Delivery** | **Health System Managerial Capacity** | **Professional Village Health Worker Program** |
| --- | --- | --- |
| - Map disease burden, define and monitor Universal Health Coverage targets - Align inputs with disease burden - Train and mentor health care workers - Initiate essential services at the health center level - Strengthen referral system - Improve availability of human resources - Improve supply chain of essential drugs and supplies | - Decentralized funding and decision making of DHMTs - Management training for district leadership - Using targets to hold health centers accountable - District and national planning, coordination and review - National technical assistance | - Adequate supervision of Village Health Workers - Clearly defined scope of work for Village Health Workers - Coordination through health centers - Payment through electronic systems - Engagement of civil society and traditional leaders |

**Legend:** The table above shows the three core areas identified as needs during the National Health Reform: Service Delivery, Health System Managerial Capacity, and the Professional Village Health Worker Program, as well as the specific areas for targeted for improvement within each area. Those areas became the focus of the interventions within the National Health Reform.

**References**

1. Half of Lesotho health budget goes to private consortium for one hospital. The Guardian. 2014 Available at: <https://www.theguardian.com/world/2014/apr/07/lesotho-health-budget-private-consortium-hospital> Accessed June 24, 2021.

2. World Health Organization. Declaration of Alma-Ata: International Conference on Primary Health Care, Alma-Ata, USSR, 6-12 1997. Available at: <https://www.who.int/publications/almaata_declaration_en.pdf> Accessed July 13, 2021.

3. Andriessen PP, van der Endt RP, Gotink MH. The village health worker project in Lesotho: an evaluation. Trop Doct. 1990;20(3):111-3.

4. Mukherjee JS, Mugunga JC, Shah A, Leta A, Birru E, Oswald C, et al. A practical approach to universal health coverage. Lancet Glob Health. 2019;7(4):e410-e1.

5. Partners In Health: Universal Health Coverage Monitoring & Planning Tool. Available at: <https://www.pih.org/practitioner-resource/universal-health-coverage-monitoring-planning-tool> Accessed June 18, 2021.

6. The World Bank. Lesotho’s Health Sector: Findings & Lessons from the 2017 Public Health Sector Expenditure Review. Available at: <https://www.worldbank.org/en/topic/health/brief/lesothos-health-sector> Accessed May 31, 2021.

7. Lesotho Population-Based HIV Impact Assessment. November 2017. Available at: <https://phia.icap.columbia.edu/wp-content/uploads/2018/02/Lesotho-Summary-Sheet_A4.2.7.18.HR_.pdf> Accessed June 23, 2021.

8. World Health Organization and Health Action International: Access to Essential Medications. Available at: <https://www.who.int/healthinfo/systems/WHO_MBHSS_2010_section4_web.pdf?ua=1> Accessed June 18, 2021.

9. Partners In Health. Annual Report on Technical Support: Pharmacy and Medical Supply Chain Management (Fiscal Year 2018). Lesotho. Unpublished Data.

10. Lega F, Prenestini A, Spurgeon P. Is management essential to improving the performance and sustainability of health care systems and organizations? A systematic review and a roadmap for future studies. Value Health. 2013;16(1 Suppl):S46-51.

11. Wong R, Hathi S, Linnander EL, El Banna A, El Maraghi M, El Din RZ, et al. Building hospital management capacity to improve patient flow for cardiac catheterization at a cardiovascular hospital in Egypt. Jt Comm J Qual Patient Saf. 2012;38(4):147-53.

12. Kebede S, Mantopoulos J, Ramanadhan S, Cherlin E, Gebeyehu M, Lawson R, et al. Educating leaders in hospital management: a pre-post study in Ethiopian hospitals. Glob Public Health. 2012;7(2):164-74.

13. Mutale W, Stringer J, Chintu N, Chilengi R, Mwanamwenge MT, Kasese N, et al. Application of balanced scorecard in the evaluation of a complex health system intervention: 12 months post intervention findings from the BHOMA intervention: a cluster randomised trial in Zambia. PLoS One. 2014;9(4):e93977.

14. Heerdegen ACS, Aikins M, Amon S, Agyemang SA, Wyss K. Managerial capacity among district health managers and its association with district performance: A comparative descriptive study of six districts in the Eastern Region of Ghana. PLoS One. 2020;15(1):e0227974.

15. Fetene N, Canavan ME, Megentta A, Linnander E, Tan AX, Nadew K, et al. District-level health management and health system performance. PLoS One. 2019;14(2):e0210624.

16. Nambiar B, Hargreaves DS, Morroni C, Heys M, Crowe S, Pagel C, et al. Improving health-care quality in resource-poor settings. Bull World Health Organ. 2017;95(1):76-8.

17. Brinkerhoff DW. Accountability and health systems: toward conceptual clarity and policy relevance. Health Policy Plan. 2004;19(6):371-9.

18. Gullo S, Galavotti C, Sebert Kuhlmann A, Msiska T, Hastings P, Marti CN. Effects of the Community Score Card approach on reproductive health service-related outcomes in Malawi. PLoS One. 2020;15(5):e0232868.

19. Gullo S, Galavotti C, Altman L. A review of CARE's Community Score Card experience and evidence. Health Policy Plan. 2016;31(10):1467-78.

20. Palazuelos D, Farmer PE, Mukherjee J. Community health and equity of outcomes: the Partners In Health experience. Lancet Glob Health. 2018;6(5):e491-e3.

21. Gunderson JM, Wieland ML, Quirindongo-Cedeno O, Asiedu GB, Ridgeway JL, O'Brien MW, et al. Community Health Workers as an Extension of Care Coordination in Primary Care: A Community-Based Cosupervisory Model. J Ambul Care Manage. 2018;41(4):333-40.

22. Scott K, Beckham SW, Gross M, Pariyo G, Rao KD, Cometto G, et al. What do we know about community-based health worker programs? A systematic review of existing reviews on community health workers. Hum Resour Health. 2018;16(1):39.

23. Witmer A, Seifer SD, Finocchio L, Leslie J, O'Neil EH. Community health workers: integral members of the health care work force. Am J Public Health. 1995;85(8 Pt 1):1055-8.

24. Partners In Health: Accompagnateur Training Guide. Available: <https://www.pih.org/practitioner-resource/accompagnateur-training-guide/accompagnateur-training-guide> Accessed June 1, 2021.

25. Satti H, Motsamai S, Chetane P, Marumo L, Barry DJ, Riley J, et al. Comprehensive approach to improving maternal health and achieving MDG 5: report from the mountains of Lesotho. PLoS One. 2012;7(8):e42700.
