# Supplementary material for: Improving access to health services through health reform in Lesotho: Progress made towards achieving Universal Health Coverage
Source: PLOS Glob Public Health. 2022 Nov 16;2(11):e0000985. doi: 10.1371/journal.pgph.0000985 (PMC10021396; doi:10.1371/journal.pgph.0000985)
Supplement: S3 Text — Deviations from the protocol are highlighted in the methods section of the text. (PDF) [file pgph.0000985.s004.pdf]

A Comprehensive Evaluation of Lesotho's National Primary Health Care Reform

Partners in Health-Lesotho/

Bo-Mphato Litsebeletsong tsa Bophelo

Protocol #1

September 2017

Table of Contents

1. Study team ... 3
2. Introduction/Background ... 3
   a) Historical and Scientific Background ... 3
   b) Statement of the Problem ... 4
   c) Rationale and Justification ... 4
   d) Key Policy Strategies ... 5

2. Literature Review ... 5

3. Research Objectives ... 7
4. Expected Outputs/Outcomes of Study ... 8
5. Research Design and Methods ... 9
6. Limitations of Study ... 10
7. Ethical consideration ... 10
8. Timetable for Study ... 12
9. Dissemination plan ... 12
10. Budget ... 13
11. List of acronyms ... 13
12. Annexes ... 14
13. Summary of proposal ... 14
14. References ... 14

1. Study team

Status in the study	Name	Institutional/Organizational Affiliations
Principal Investigator	Dr. Abera Leta, MD MPH	Partners in Health
Co-Principal Investigator	Dr. Joia Mukherjee, MD MPH MS	Partners in Health / Brigham and Women's
Co-Investigator	Dr. Ryan McBain, PhD MPH	RAND Corporation
Co-Investigator	Dr. Melino Ndayizigiye, MD MMSc	Partners in Health
Co-Investigator	Dr. Letsie Nyane MD	Lesotho Ministry of Health
Co-Investigator	Ermyas Birru, MSc	Partners in Health
Key Personnel	Dr. Afom Andom, MD	Partners in Health
Key Personnel	Likhapha Ntlamelle, MA	Partners in Health
Key Personnel	Seyfu Abebe, MPH	Partners in Health
Key Personnel	Joalane 'Mabathoana	Partners in Health
Key Personnel	Sophie Motsamai	Partners in Health
Key Personnel	Palesa Chetane	Partners in Health
Key Personnel	Relebohile Lebatla	Partners in Health
Key Personnel	Qenehelo Matjama	Partners in Health
Key Personnel	Patrick Smith	Partners in Health
Key Personnel	Collin Whelley, MPP	Partners in Health
Key Personnel	Joel Curtain, MPH	Partners in Health

Are the CV's attached with the protocol? Yes/No

2. Introduction/Background

a) Historical and Scientific Background

Lesotho has poor population health outcomes, in particular for high-burden disease areas including HIV, tuberculosis, and maternal, newborn, and child health. Recognizing these population needs, the country has demonstrated a strong commitment to improving health outcomes: national health expenditure in 2014 was 8.1% of gross domestic product (GDP), equating to a per capita expenditure of $105, the 14th highest in Africa (WHO, 2014). However, the MoH budget has historically not been focused on priority needs, with largest allocations going to tertiary care, district hospitals, and planning.

Lesotho is one of the world's poorest countries, ranking 160th out of 188 countries in the United Nations Development Programme (UNDP) Human Development Index (UNDP, 2016). The wealthiest households are concentrated in urban areas, with 85% of the urban population belonging to the two highest wealth quintiles. According to the Ministry of Health's Global AIDS Response Progress Report, the burden of HIV is one of the highest in the world with an adult HIV prevalence rate of 25.0% (Ministry of Health, 2015). This high prevalence increases the vulnerability of Basotho to opportunistic infections that could otherwise be effectively managed by primary care providers (PCP); viewed more broadly, this epidemic serves as both a cause and effect of economic underdevelopment and poor health outcomes. Antiretroviral therapy coverage for both adults and children remains alarmingly low at an estimated 35% and 30% respectively, and consequently, mother-to-child transmission of HIV remains significant

(Ministry of Health, 2015). As HIV rates remain high, life expectancy has declined significantly in recent years, dropping from 61.2 in 1990 to 48.3 in 2014 (Ministry of Health, 2015).

One notable complication of Lesotho's high HIV prevalence is an alarmingly high incidence of TB. Despite recent decreases in reported TB incidence, the 2016 Global Tuberculosis Report indicates that Lesotho's TB incidence (788 per 100,000 population) remains one of the highest in the world; and an estimated 71.8% of TB cases are co-infected with HIV (World Health Organization, 2016). Further, current treatment seeking behavior is low: per the 2014 LDHS, 50.5% of men and 40.9% of women aged 15-49 who exhibited symptoms of TB did not seek further evaluation or treatment (Ministry of Health and ICF International, 2016). These factors have resulted in a low TB treatment success rate and a declining rate of TB case notification in Lesotho (Ministry of Health, 2015).

Lesotho has high maternal and child mortality rates at 1,024 deaths/100,000 live births and 85 deaths/1,000 live births respectively, and 33% of children under age 5 are stunted (Ministry of Health and ICF International, 2016). Further, according to LDHS (2014), only 41% of women had their first antenatal care (ANC) visit during the first trimester of pregnancy, and only 74% had the recommended four or more visits in the course of their pregnancy. Estimates from the 2014 LDHS indicate that the lifetime risk of maternal mortality is 3.2% and not significantly decreased from previous reports (Ministry of Health and ICF International, 2016).

b) **Statement of the Problem**

A distinct lack of managerial capacity and resources at the district level, complex and confused operational lines within the MoH and a lack of accountability presented significant barriers to the delivery of quality primary health care (PHC) services. Despite relatively high health expenditure, only 5% of spending was controlled by District Health Management Teams (DHMTs), where the bulk of PHC delivery occurs.

Impacts of geographic and financial factors on accessibility and quality of primary care are twofold, inhibiting care accessibility for patients and restricting the provision of staff, medical products, and strategic information to care delivery points. Such challenges have historically been compounded by poor transportation and health-related infrastructure, and by fragmentation of care between service providers and levels of the health system. While recent interventions – such as investments in infrastructure – have addressed some of these challenges, substantial barriers to comprehensive and patient-centered care remain. Lesotho also faces challenges in delivering quality primary health care services due to major verticalization of resources, especially for HIV and TB programming, resulting in system inefficiencies and missing opportunities to integrate and deliver comprehensive services.

c) **Rationale and Justification**

Given slow progress towards addressing priority health challenges, and following an assessment of the health system, the Ministry of Health launched a 5-year National Primary Health Care (PHC) Reform in 2014. The key aspect of the reform are: empowerment and reorganization District Health Management Teams (DHMTs), improved service delivery at the health center level, and a strengthened and expanded village health worker (VHW) program. With Partners In

Health (PIH) serving as a technical advisor, a pilot of the Reform was introduced in April 2014 across four districts: Berea, Butha-Buthe, Leribe, and Mohale's Hoek.

The aim of the current project is to comprehensively evaluate the impacts of the National PHC Reform on the public health system. Using the World Health Organization's Six Building Blocks of Health Systems as a conceptual framework, we will evaluate the Reform's quantifiable impact on service delivery, the health workforce, health information systems, health financing, availability of essential medical products, and leadership and governance (World Health Organization, 2007). Employing both quantitative and qualitative methods, this project will evaluate the Reform from numerous perspectives, describing its model, implementation process, and impacts to-date, as well as next steps for the primary healthcare system in Lesotho. A comprehensive evaluation of this Ministry of Health-led intervention will facilitate a more nuanced understanding of how the Reform has worked to date. Moreover, it will aim to inform the authorship of several policy documents and high-impact journal articles that describe the model, implementation process, and impact of the national PHC Reform.

d) **Key Policy Strategies**

- Articulate guiding principles for the technical model and leadership approach to PHC in Lesotho
- Reinforce Ministry of Health (MoH) targets for PHC goals for a set of key disease areas so that all stakeholders can work towards these targets and leaders can be held accountable for their performance.
- Based on the priorities of and guidelines from the Ministry of Health, define the technical model for PHC delivery in the districts so that all stakeholders understand and use the same health systems approach to delivering PHC
- In line with guidelines from the Ministry of Health and Lesotho Decentralization policies, define clear roles, responsibilities, and reporting lines for all stakeholders in order to promote accountability and a clear process for escalating issues
- Formalize processes using Health Management Information System (HMIS) and other monitoring and evaluation (M&E) tools to help managers improve performance and promote accountability and a clear process for escalating issues
- Estimate the total cost of implementing the PHC Reform, and provide gap analysis to identify areas where the risk of critical resource shortfalls is high and where additional allocations need to be made and/or resource mobilization needs to be undertaken

2. Literature Review

Health Systems Strengthening in Lower-Middle Income Countries

Internationally, growing investments in global health have led numerous experts to comment on the need for strengthened health systems which can effectively translate this investment into improved outcomes; this, coupled with shifts in the strategies embraced by major funders, has resulted in increased funding for interventions that strengthen the overall health system (as

opposed to verticalized interventions targeting a single condition) (Evans, Nishtar, Atun, & Etienne, 2008) (Shakarishvili, et al., 2011). This funding has created a pathway for new partnerships between governmental and non-governmental bodies in strengthening global health systems, as international recommendations and experiences in diverse contexts have shown (Drobac, et al., 2013) (Jerome & Ivers, 2010) (World Health Organization, 2007). To promote greater understanding of models and processes that can effectively strengthen systems – and of the contexts in which existing models have been successful – there is a need for comprehensive and holistic evaluations that assess the impact of such interventions on numerous sectors of the health system (Swanson, Atun, Nishtar, & Chunharas, 2014) (Frenk, 2010).

However, a recent systematic review by Adam et al. (2012) has revealed that few health system evaluations conducted to date have used a comprehensive and holistic approach to assess the impact of interventions on multiple health systems "building blocks." Due to the potentially diffuse nature of system-focused interventions, it is particularly important that evaluations utilize a structured and comprehensive design that considers multiple dimensions of the health system and the complex interactions that may occur between them. To date, few evaluations have been conducted that comprehensively address these needs, due to a range of reasons which may include limited capacity, inadequate funding or time, or a lack of demand from researchers and funders (Adam, et al., 2012).

Recognizing this knowledge gap, it is important that future research use is known best practices and transparent models to evaluate the impacts of interventions using a structured framework (Frenk, 2010). Such research projects should also include evaluations of the process and context associated with system strengthening interventions, as these components will strengthen analysis and facilitate a greater understanding of the global relevance of the intervention (Adam, et al., 2012). To date, no publically available research has methodically described Lesotho's primary healthcare system and its areas for growth. Address knowledge gaps on health systems strengthening and system evaluation – in Lesotho and the broader international community – requires a detailed and methodologically rigorous approach.

Justification of Current Methods

Acknowledging substantial knowledge of gaps in Lesotho and the broader global community, the aims of the present project are two-fold: firstly, the evaluation will assess and document the model of the national PHC Reform and the process of its implementation to date, including consideration of the various factors (within and outside the health sector) which may have supported or inhibited its implementation. Secondly, the evaluation will rigorously assess the impact of the intervention on health systems outputs and outcomes, including its direct and indirect effects on each of the World Health Organization's Six Building Blocks of Health Systems as a framework, we will evaluate the Reform's quantifiable impact on service delivery, the health workforce, health information systems, health financing, availability of essential medical products, and leadership and governance. Use of qualitative and quantitative methods will enable us to conduct an evaluation that is holistic in nature, supportive to Lesotho's further efforts in system strengthening, and relevant to the global community.

The usefulness of key informant interviews in model and process evaluation is well-established, and the use of a broad, open-ended questionnaire is supported by current research (Adam, et

al., 2012). The selection of broad-ranging indicators which cover each health system building block is grounded in an understanding of the many inter-related factors which comprise an effective health system (WHO, 2010). Likewise, the use of interrupted time series regression analysis is a known best practice for evaluating public health interventions including national public health legislation (Bernal, Cummins, & Gasparrini, 2016) (Kontopantelis, Doran, Springate, Buchan, & Reeves, 2015). Recent examinations have illustrated that this methodological approach may be effectively used to evaluate the impact of public health interventions on service readiness in low-resource contexts, provided that data is available with an adequate number of time points and a clearly defined intervention point (Iyer, et al., 2017). Facility based data extraction will enable the collection of high-quality data, and a mixed-methods approach will allow for the construction of a robust statistical model. In short, the methods proposed by this evaluation will leverage known best practices as well as the specific skills of the research team.

A comprehensive evaluation of this Ministry of Health-led intervention will facilitate a more nuanced understanding of how the Reform has worked to date and will support further strengthening of the primary health system in Lesotho – in pilot Reform districts and the nation as a whole. Moreover, this evaluation will inform the authorship of policy documents and high-impact journal articles that describe the model, implementation process, and impact of the national PHC Reform. Publications discussing the key lessons learned from the Reform and the evaluation process will address key research gaps and expand the knowledge base on health systems strengthening. Moreover, this project will provide insight for future evaluations of public health interventions, thereby helping the global community strive towards our shared goal of health for all.

3. Research Objectives

An in-depth articulation of the reform model and its various components will be helpful for documentation, advocacy, and replication throughout Lesotho; measuring impact will allow for assessment as to whether the model has been successful in meeting improved health targets; and qualitative interviews with stakeholders should provide framing of the context in which the reform has unfolded to-date, including lessons learned as the reform process continues. There are three main components of this evaluation:

- COMPONENT 1 | MODEL & PROCESS
    - The first of these components will represent a qualitative synthesis of the constituent parts that, in totality, make up the reform. From an operational standpoint, systematically documenting these components is necessary as a field guide for the Ministry and partner organizations—particularly as activities expand and stabilize in the coming years.
- COMPONENT 2 | IMPACT
    - The second evaluation component will comprise an impact evaluation of the reform to-date. The impact of the reform shall be concluded in two parts:

1. Whether the framework articulated in Component 1 above has been successfully instituted, in terms of metrics on each WHO Health systems building blocks
2. Whether the reform has met its stated objectives of improving population health, focusing specifically on service delivery component of the WHO health system building blocks.

- COMPONENT 3 | INTERPRETATION
    - The final component of the evaluation will be an engagement of key stakeholders who have been involved in the reform to-date. Qualitative interviews will complement quantitative data analysis to answer the how and why questions pertaining to successes and shortcomings in the roll-out and implementation of the reform. This will generate feedback for quality improvement moving forward.

4. Expected Outputs/Outcomes of Study

- A detailed, 20-page policy report that documents the model of the Reform, the implementation process, and its impact to date. This document is intended to support the Ministry of Health and to formally define, for external stakeholders, the component parts of the Reform. Quantitative and qualitative assessment methods will be complemented by a complete review of existing literature.
- A "user guide" that clearly describes the component parts of the "Reform" through the lens of the WHO Building Blocks of Health Systems and concretely defines the steps that must be taken at the national, district, and facility levels to achieve Reform.
- A thorough quantitative evaluation of the Reform's key impacts on service delivery and additional building blocks, for publication in a high-impact, internationally-focused biomedical journal
- One or more detailed quantitative evaluations of the Reform's impact on specific content areas – such as HIV/AIDS, maternal-child health, or scale-up of national community health worker programs.
- Commentary on the key lessons learned through the process of implementing the Reform, according to key staff at PIH who have played key roles in supporting the Reform to date.

  An explanation of key lessons learned from the process of implementing the National PHC Reform, focusing on the exploration of key successes and challenges identified by key informants who were engaged throughout the design and implementation phases of the Reform.

5. Research Design and Methods

- ***Study area and population***: The population under consideration includes all 72 public facilities in the four Reform Pilot districts of Lesotho: Berea, Butha-Buthe, Leribe and Mohale's Hoek. In addition to quantitative data collection at these facilities, we will interview key stakeholders at these locations to better understand progress and barriers to the implementation of health reform content.
- ***Sample Size***: We will conduct data collection at n=72 health centers throughout the four districts. Additionally, we will interview senior PIH Officials, Ministry Officials, Facility Directors in Reform Districts, as well as in the capital city of Maseru (n=20).
- ***Determination of sample size***: The sample size for quantitative data collection is fixed at n=72 because this is the number of facilities in operation. For qualitative data collection, we are implementing a stratified design to interview members for specific roles (n=5) in each of the 4 districts, for a total of 20 interviews.
- ***Sampling method***: For quantitative data collection, we are conducting population-based data collection. For qualitative data collection, we will be implementing purposive (snowball) sampling to identify relevant key informants
- ***Type of data***: Quantitative data are de-identified aggregate health metrics at the facility-level on a monthly basis, dating back over the previous 3 years. This will include, for example, the total number of admissions, the number of HIV patients enrolled in ART, and the total number of pregnant women delivering at the facility
- ***Study design***: This is a mixed-methods study design. For the quantitative portion, we will be conducting a retrospective interrupted time series analysis (ITSA). For the qualitative portion, we will be conducting key informant interviews
- ***Independent and dependent variables (if applicable)***: The primary dependent variable in our analyses will be time elapsed since introduction of the primary healthcare reform. Independent variables include service delivery indicators for priority areas (HIV/AIDS, TB, maternal-child health, and outpatient department services) and variables which relate to the other building blocks. Please see appendices for further information.
- ***Data collection methods, tools for the study, translate if necessary- attached as annex***: Please see appendices. Quantitative data will be gathered on electronic tablets that are encrypted and password protected, and are locked at night with a key carried by the research team. The team will move from facility to facility, collecting these metrics over a 6-month period. Qualitative information will be stored using an audio recorder, also stored by the research team in a locked file cabinet.
- ***Plan for analysis***: Interrupted time series analysis, using a regression approach in STATA 14.0, will be implemented for the quantitative part. Thematic content analysis, using Dedoose software, will be used for the qualitative piece.
- ***Plan for use of results and distribution of report, expected benefit of study:*** We intend to synthesize results in a policy report to present to the Ministry of Health, as well as in

a number of academic publications. This information will be used to inform further scale up of the national health reform in Lesotho.

- ***Data management***: As noted above, tablets will be encrypted and password protected and stored in a locked file cabinet. Audio recorders will also be locked in a file cabinet, and once data collection is complete the audio files will be removed from the recorder and stored on a password-protected computer
- ***Proposed procedures and interventions (if applicable:***) N/A. There is no intervention.
- ***Are the instrument for the study attached with the protocol*?**
  Yes/No/NA
  i. ***Study attached with the protocol*?** Yes/No/NA

6. Limitations of Study

- While the reform model seeks to improve the overall health system in Lesotho, the intervention may have been targeted to certain components of the health systems building blocks. Due to the Reform's focus on improving leadership and governance, service delivery, human resources, and supply chain, this study may not uncover system-wide impacts for all building blocks. In order to ensure the neutrality and comprehensiveness of this evaluation, we are using a mixed methods approach that will afford us open insight into the varied and intersectional impacts of the Reform.
- This evaluation is seeking to identify the impact of a complex, multi-pronged intervention on the overall strength of the primary care system. It is possible that the evaluation may not capture the full impacts of the intervention, given the long duration of time that it may take for large-scale public health interventions to take hold.
- Baseline data collected pre-reform implementation may not have captured all the necessary data elements need for this evaluation. Retrospective data collection is necessary to address some of the expected gaps from the initial baseline.
- Given the Reform's focus on improving numerous aspects of the primary healthcare system, including health information systems, it is anticipated that the implementation of the Reform will be associated with notable increases in the saturation and accuracy of data. In order to account for potential variations in the quality of publically available data, this evaluation will extract data from facility-level registers.

7. Ethical consideration (Recruitment, Informed consent form, Privacy, Confidentiality of data, Role of the sponsor (if applicable)

- Recruitment will take place by email and phone according to a pre-established script. Potential participants will receive information on the aims, methods, and expected time burden associated with participation, and all participation will occur voluntarily.

- All persons involved in the project planning, data analysis, and communication stages of this project have completed training on research ethics, including the foundational principles of beneficence, respect for persons, and justice. Persons responsible for primary quantitative and qualitative data collection will also be trained on key ethical issues in research, including foundational principles and their relation to the project at hand.
- The Director General of the Ministry of Health has authorized the collection of data from all facilities in the four Reform pilot districts. Prior to the data collection phase, all districts and facilities will be consulted and provided with a schedule for facility visits to ensure that the data collection process is minimally disruptive to clinical service delivery.
- Prior to enrollment in either of the qualitative components of this study, potential participants will be provided with a written and verbal overview of the purpose, procedure, eligibility criteria, and risks and benefits associated with the current project. Written consent will be obtained for all participants; in the event that a participant is unable to provide written consent, verbal consent will be obtained.
- This study will not collect any protected medical information from participants. However, because interviews may discuss information that is considered politically sensitive, interview recordings and related transcripts will be encrypted at rest following their transfer to password-protected devices. All participant data will be kept confidential, and potentially identifying information will be excluded from data analysis and presentation in order to minimize potential harm to subjects.
- Are the informed consent form/s attached to the proposal? Yes/No

8. Timetable for Study: please see attached project timetable

	Month	Jul	Aug	Sep	Oct	Nov	Dec	Jan	Feb	Mar	Apr	May	Jun
	**Reform Evaluation Timeline**	Prep				Data collection				Writing			
				Prep				Data analysis			Editing		
**Preparation**	Finalize qualitative instruments	x	x										
	Finalize quantitative tools	x	x										
	Obtain ethical (IRB) approval	x	x	x									
	Buy project materials	x											
**Qualitative**	Conduct KI Interviews			X	X	x							
	Transcribe & translate KI interviews			X	X	X	x						
	Code KI interviews						x						
	Summarize key qualitative findings							x					
**Quantitative**	Recruit and train data collectors		X	X									
	Oversee quantitative data collection			X	X	X	X	X	X				
	Conduct quantitative (ITSA) analysis									X			
	Summarize key quantitative findings										x		
**Reporting**	Conduct thorough literature review								X	x			
	Write first drafts of manuscripts								x	x	X		
	Review manuscript drafts										X	x	
	Finalize report												x
	Host workshop, distribute report												x

9. Dissemination plan:

The goals of this evaluation are twofold: firstly, the Reform evaluation will inform the development of policy documents that offer a framework for the primary healthcare system in Lesotho; secondly, it will aim to communicate (to academic audiences, policymakers, and external stakeholders) the design, implementation, and impact of the National Health Reform, offering insight to key lessons learned from the experience of introducing the Reform. We will host workshops and meetings in Lesotho where findings and results will be shared with key policymakers and local stakeholders. Outside of Lesotho, we plan to share these outputs will be shared in global conference settings, and similar venues involving discussion and sharing of health system strengthening practices. The findings, recommendations, and lessons from this evaluation will also be of great value to the global practices and operations of Partners In Health, they will serve to inform our work around the world. We plan to author several journal articles and policy documents (described above). Academic articles will be submitted to

journals in May and June 2018, following data analysis and authorship; policy reports and user guides will be communicated to the Ministry of Health and other stakeholders through a joint workshop, and PIH leadership plan to communicate with Ministry officials regarding Reform modification and scale-up based on the key lessons gleaned from this evaluation.

10. **Budget:** This project is primarily funded through grants from the Skoll Foundation and the Crown Family Foundation. Additional funding has been contributed by Partners In Health's unrestricted funding sources. The total funding received for this project is USD $400,000, including $200,000 from the Skoll Foundation, $100,000 from the Crown Family Foundation, and $100,000 from PIH unrestricted funding sources. Please see the attached project budget for further information.

11. **List of acronyms:**

AIDS – Acquired Immunodeficiency Syndrome

ANC – Antenatal Care

DHMT – District Health Management Team

GDP – Gross Domestic Product

HIV – Human Immunodeficiency Virus

HMIS – Health Management Information Systems

IRB – Institutional Review Board

ITSA – Interrupted Time Series Analysis

KI – Key Informant

LDHS – Lesotho Demographic and Health Survey

M&E – Monitoring and Evaluation

MCH – Maternal-Child Health

MoH – Ministry of Health

PCP – Primary Care Provider

PHC – Primary Healthcare

PIH – Partners In Health

TB – Tuberculosis

UNDP – United Nations Development Programme

WHO – World Health Organization

12. Annexes:

a. Recruitment Script
b. Informed Consent Form (English and Sesotho)
c. Structured Key Informant Interview Guide
d. Open-Ended Interview Guide (English and Sesotho)
e. Budget and detailed budget narrative
f. List of quantitative indicators to be gathered
g. Curriculum vitae of Co-investigators

13. Summary of proposal

This project will address substantial domestic and international knowledge gaps by rigorously and comprehensively assessing Lesotho's primary healthcare system through the lens of the World Health Organization's Six Building Blocks of Health Systems. Using internationally recognized best practices for data analysis, this evaluation will serve as an example for future projects that aim to evaluate the impacts of system-building interventions on population-level outcomes. Moreover, the findings of this study will directly support Lesotho by enhancing our understanding of success and challenges associated with the Reform. As the Reform is improved in pilot districts and brought to scale nationwide, we expect that the information gathered from this project will support the creation of more efficient and effective systems.

14. References

Adam, T., Hsu, J., de Savigny, D., Lavis, J. N., Rottingen, J.-A., & Bennett, S. (2012). Evaluating health systems interventions in low-income and middle-income countries: are we asking the right questions? *Health Policy and Planning*, iv9-19.

Bernal, J. L., Cummins, S., & Gasparrini, A. (2016). Interrupted time series regression for the evaluation of public health interventions: a tutorial. *International Journal of Epidemiology*, 348-355.

Drobac, P. C., Basinga, P., Condo, J., Farmer, P. E., Finnegan, K. E., Hamon, J. K., . . . Binagwaho, A. (2013). Comprehensive and integrated district health systems strengthening: the Rwanda Population Health Implementation and Training (PHIT) Partnership. *BMC Health Services Research*.

Evans, T., Nishtar, S., Atun, R., & Etienne, C. (2008). Scaling up research and learning for health systems: time to act. *The Lancet*, 1529-31.

Frenk, J. (2010). The Global Health System: Strengthening National Health Systems as the Next Step for Global Progress. *PLOS Medicine*.

Iyer, H. S., Kamanzi, E., Mugunga, J., Finnegan, K., Uwingabiye, A., Shyaka, E., . . . Drobac, P. C. (2017). Improving district facility readiness: a 12-month evaluation of a data-driven health systems strengthening intervention in rural Rwanda. *Global Health Action*.

Jerome, J., & Ivers, L. (2010). Community Health Workers in Health Systems Strengthening: A qualitative evaluation from rural Haiti. *AIDS*, S67-S72.

Kontopantelis, E., Doran, T., Springate, D., Buchan, I., & Reeves, D. (2015). Regression based quasi-experimental approach when randomisation is not an option: interrupted time series analysis. *BMJ*.

Ministry of Health. (2015). *Global AIDS Response Progress Report.* Maseru.

Ministry of Health. (2015). *Tuberculosis - 2015 Annual Report.* Maseru, Lesotho.

Ministry of Health and ICF International. (2016). *Lesotho Demographic and Health Survey 2014.* Maseru.

Shakarishvili, G., Lansang, M. A., Mitta, V., Bornemisza, O., Blakley, M., Kley, N., . . . Atun, R. (2011). Health systems strengthening: a common classification and framework for investment analysis. *Health Policy and Planning*, 316-326.

Swanson, R. C., Atun, R., Nishtar, S., & Chunharas, S. (2014). Systems thinking for the post-2015 agenda. *The Lancet*, 2124-5.

UNDP. (2016). *Human Development Report.* New York City.

WHO. (2010). *Monitoring the Building Blocks of Health Systems: A Handbook of Indicators and their Measurement Strategies.* Geneva.

WHO. (2014). *Global Health Expenditure Database.* Geneva.

World Health Organization. (2007). *Everybody's Business: Strengthening Health Systems to Improve Health Outcomes.* Geneva: World Health Organization.

World Health Organization. (2016). *Global Tuberculosis Report: 2016.* Geneva.
