# Supplementary material for: Improving access to health services through health reform in Lesotho: Progress made towards achieving Universal Health Coverage
Source: PLOS Glob Public Health. 2022 Nov 16;2(11):e0000985. doi: 10.1371/journal.pgph.0000985 (PMC10021396; doi:10.1371/journal.pgph.0000985)
Supplement: S4 Text — (PDF) [file pgph.0000985.s005.pdf]

DHMT Data Collection Sheets

Name of District: ____________________

Date of Data Collection: ____________________

Name of Person collecting Data: ____________________

Notes:

Record number	District	Clinic	Position	Start Date of Employment (Day/Month/Year)	End Date of Employment (Day/Month/Year)
1					
2					
3					
4					
5					
6					
7					
8					
9					
10					
11					
12					
13					
14					
15					
16					
17					
18					
19					
20					
21					
22					
23					
24					
25					
26					
27					

Number of reports received [Clinic name] ______________

	2013						2014						
	July	August	Sept	Oct	Nov	Dec	Jan	Feb	March	April	May	June	July
# of reports received													
# of reports received on time													
# of people in catchment area													
	2014						2015						
	July	August	Sept	Oct	Nov	Dec	Jan	Feb	March	April	May	June	July
# of reports received													
# of reports received on time													
# of people in catchment area													
	2015						2016						
	July	August	Sept	Oct	Nov	Dec	Jan	Feb	March	April	May	June	July
# of reports received													
# of reports received on time													
# of people in catchment area													
	2016						2017						
	July	August	Sept	Oct	Nov	Dec	Jan	Feb	March	April	May	June	July
# of reports received													
# of reports received on time													
# of people in catchment area													

Number of DHMT meetings held per Month													
	2013						2014						
	July	August	Sept	Oct	Nov	Dec	Jan	Feb	March	April	May	June	July
# of meetings													
	2014						2015						
	July	August	Sept	Oct	Nov	Dec	Jan	Feb	March	April	May	June	July
	2015						2016						
	July	August	Sept	Oct	Nov	Dec	Jan	Feb	March	April	May	June	July
# of meetings													
	2016						2017						
	July	August	Sept	Oct	Nov	Dec	Jan	Feb	March	April	May	June	July
# of meetings													

Number of VHWs for [Clinic name] _______________													
	2013						2014						
	July	August	Sept	Oct	Nov	Dec	Jan	Feb	March	April	May	June	July
# of MMRP VHWs													
# of HIV/TB VHWs													
	2014						2015						
	July	August	Sept	Oct	Nov	Dec	Jan	Feb	March	April	May	June	July
# of MMRP VHWs													
# of HIV/TB VHWs													
	2015						2016						
	July	August	Sept	Oct	Nov	Dec	Jan	Feb	March	April	May	June	July
# of MMRP VHWs													
# of HIV/TB VHWs													
	2016						2017						
	July	August	Sept	Oct	Nov	Dec	Jan	Feb	March	April	May	June	July
# of MMRP VHWs													
# of HIV/TB VHWs													

Clinic functionality [Clinic name] ______________

	2013						2014						
	July	August	Sept	Oct	Nov	Dec	Jan	Feb	March	April	May	June	July
Clinic has Water													
Clinic has power													
Clinic has medication													
Clinic has computer													
	2014						2015						
	July	August	Sept	Oct	Nov	Dec	Jan	Feb	March	April	May	June	July
Clinic has Water													
Clinic has power													
Clinic has medication													
Clinic has computer													
	2015						2016						
	July	August	Sept	Oct	Nov	Dec	Jan	Feb	March	April	May	June	July
Clinic has Water													
Clinic has power													
Clinic has medication													
Clinic has computer													
	2016						2017						
	July	August	Sept	Oct	Nov	Dec	Jan	Feb	March	April	May	June	July
Clinic has Water													
Clinic has power													
Clinic has medication													
Clinic has computer													

Script for Reform User Guide Interviews
8 Aug 2017

PART 1: WARM-UP

1. Let's start with you telling me a little about yourself: Your name, as well as where you are from and a little about your background and current job.

2. How have you been involved in the National Primary Healthcare Reform efforts?
   Note: If any of the following topics DON'T come up during discussion, probe further.
   a. With what institution(s) have you been involved in the Reform? Where?
   b. What role(s) have you played within that institution in relation to the Reform?
   c. When were you involved in these efforts? If in the past, when?
   Note: If the respondent has worked in multiple organizations or roles, probe further to ensure that the above questions are answered for every role/organization/area they have worked in.

[TRANSITION] Thank you. Now that I have an understanding of your background, expertise and role in the Reform, I'd like to ask some descriptive questions—just so we can better understand the Reform from your perspective.

PART 2: HISTORICAL OVERVIEW

1. Could you describe, as best as you can, your understanding of what the Health Reform is supposed to achieve for Lesotho? I know this is a big question, so feel free to take your time.

2. Are there aspects of the Reform that are not clear to you? If so, can you tell me more about what aspects are unclear?

[TRANSITION] Ok, thank you. Let's take a moment to talk a bit more about some specific areas of the health system. For each of these areas, it would be helpful to know – from *your* perspective on the Reform – what was supposed to happen, what has happened, and what still needs to be done. Because this next section covers a lot of material, you may not know the answer to some of the questions. If this is the case, please feel comfortable saying "I don't know."

iii. Pharmacy & lab staff - pharmacist, pharmacy technician, lab technicians
iv. Other personnel

3. Okay, thank you. Was the Reform supposed to introduce any changes in the way that staffing gaps are monitored and filled? If so, what changes were supposed to occur?

4. Was the Reform supposed to change anything about the way that health workers receive ongoing training (e.g. through workshops)? If so, can you tell me more about this?

5. Are there any other ways that the Reform was supposed to change the health workforce that you haven't mentioned yet, but would like to? If so, what are these?

[TRANSITION] Okay, great. Let's transition again and talk a bit about leadership in the health system.

PART 5: LEADERSHIP & GOVERNANCE

1. When the Reform was introduced, was anything supposed to change in terms of the roles served by the central MOH, DHMTs, facilities, or local governments? If so, how were roles supposed to change?

2. Was the Reform intended to change anything about the way that problems in the health sector are solved? If so, can you tell me more?

3. Was the Reform supposed to introduce any changes to health policies, such as national health strategies or policies for specific issues (e.g. TB, HIV/AIDS, or maternal-child health)? If so, can you tell me more?

4. Are there any other ways in which the Reform was supposed to change the leadership structure of the health system that we haven't discussed yet? If so, what are these?

[TRANSITION] Okay, thank you. Moving on, let's talk about the way that money is managed within the health system.

PART 6: FINANCING

1. Was the Reform supposed to change anything about the management of funds at the health center, district, or national levels? If so, can you tell me more?

2. Was the Reform supposed to change anything about the way that health providers – such as nurses, village health workers, or others – are paid? If so, what?

3. Was the Reform intended to introduce any changes in the amount of money spent on health at the facility, district or national levels? If so, what was supposed to change?
   Note: if the following areas are not mentioned, probe further:
   a. How about changes in the budget utilization rate? Was the Reform supposed to introduce any change in the percentage of the budget that is used each year? If so, what?
   b. What about changes in the distribution of money – was the Reform supposed to introduce any changes in the areas where money is spent on health? If so, what?

2. How about distribution – was the Reform supposed to change anything about the way that medicines or medical products are supplied to health centers? *For instance, equipment, infection control materials, or laboratory materials?* If so, what?

3. Was the Reform intended to change the frequency or duration of medication stock-outs? If so, how?
   a. Was the Reform supposed to introduce any new treatment regimens or medical equipment? If so, what?

4. Was the Reform supposed to introduce any changes to public health efforts – such as new outreaches or vaccination efforts? If so, can you tell me more?

5. Was the Reform intended to introduce any other changes to the availability of medicines or medical products? If so what were these?

PART 9: FINAL THOUGHTS

1. Great, thank you so much for your time. Before we wrap up, I'm curious if—based on all of our discussion—you have any final reflections you would like to share?

2. As a last request, do you have any historical documentation—including guidelines, protocols, or manuals—that have supported the planning or implementation of the Reform? If so, are you willing to share a copy with me?

3. Okay, great. And one last thing: Do you have any questions for me that I can answer?
