# Supplementary material for: Improving access to health services through health reform in Lesotho: Progress made towards achieving Universal Health Coverage
Source: PLOS Glob Public Health. 2022 Nov 16;2(11):e0000985. doi: 10.1371/journal.pgph.0000985 (PMC10021396; doi:10.1371/journal.pgph.0000985)
Supplement: Attachment — Submitted filename: Allan-Blitz - Response Letter - 5.31.22.docx [file pgph.0000985.s007.docx]

Dear editor of *PLOS Global Public Health,*

Thank you very much for the thoughtful comments on the paper “*Improving Access to Health Services Through Health Reform in Lesotho: Progress Made Towards Achieving Universal Health Coverage.*” We have reviewed and responded to the comments from the reviewers. Below are our responses to each comment. Please do not hesitate to contact us if anything further is needed. The manuscript has been revised accordingly, and we hope that this revised manuscript will be accepted for publication.

Sincerely,

Lao-Tzu Allan-Blitz

**Corresponding Author Contact Information:**

Dr. Lao-Tzu Allan-Blitz

Department of Medicine, Brigham and Women’s Hospital

75 Francis Street

Boston, MA, 02115, USA

Email: lallan-blitz@partners.org, phone: (805) 896-5313

**Editor’s Comments**

I agree with the reviewers that there is need to add more information in the methods section and be explicit on what was done and that should trickle to the presentation of the results which will also have a bearing on the discussion. Once this is clarified, it will highlight the assertions you are making in the conclusion.

**We have added the following to further elaborate on the methods used for study design and data analysis:**

**“Four districts were selected by the MOHSW to pilot the National Health Reform interventions: Berea, Leribe, Butha-Buthe and Mohale’s Hoek, which spanned an estimated catchment population of 815,520 individuals (40% of the country’s total population). All health facilities in those districts were included. A baseline assessment was undertaken in 2013 across 70 facilities in the four districts.”**

**“*Data Collection, Study Design and Data Analysis***

**Baseline data collection was done in all the health facilities in the four districts from July 2013 to June 2014. Routine data collection on the same indicators continued throughout the pilot phase of the health reform intervention. During the impact evaluation period, a monitoring and evaluation team from the PIH and MOHSW went to all the facilities and collected the data from the facility registers to double check and validate the routine data already collected and entered into national database. The outcome measures assessed included: average quarterly number of women attending their first and fourth antenatal care visits, average quarterly number of women attending their post-natal care visits, average quarterly number of health centers adequately equipped to provide a facility-based birth, average quarterly number of facility-based births that occurred, average quarterly number of maternal emergency referrals placed and transported to the health facility, the availability of essential medications, the proportion of patients living with HIV and enrolled in care who were lost to follow-up, the average quarterly number of HIV tests performed at health facilities, and the average quarterly number of new HIV cases detected.**

**We further utilized data from the World Bank (39) to evaluate population level demographics over time, in order to assess for potential confounding by differences in the population before and after the National Health Reform (Table 1). We then conducted a one arm pre- and post-intervention time series analysis, evaluating changes in the outcomes above after three years of operationalizing the National Health Reform compared to before (2013). Data were collected in 2017, thus that was considered the end point for the study, however the activities of the National Health Reform are ongoing. Data were analyzed using STATA software version 15.1 and interrupted time series were performed to assess the trends on different indicators.”**

JOURNAL REQUIREMENTS:

1. Please include a complete copy of PLOS’ questionnaire on inclusivity in global research in your revised manuscript. Our policy for research in this area aims to improve transparency in the reporting of research performed outside of researchers’ own country or community. The policy applies to researchers who have travelled to a different country to conduct research, research with Indigenous populations or their lands, and research on cultural artefacts. The questionnaire can also be requested at the journal’s discretion for any other submissions, even if these conditions are not met.  Please find more information on the policy and a link to download a blank copy of the questionnaire here: <https://journals.plos.org/globalpublichealth/s/best-practices-in-research-reporting>. Please upload a completed version of your questionnaire as Supporting Information when you resubmit your manuscript.

**We have submitted the questionnaire as supporting information.**

2. In the online submission form, you indicated that "Data are available upon request". All PLOS journals now require all data underlying the findings described in their manuscript to be freely available to other researchers, either 1. In a public repository, 2. Within the manuscript itself, or 3. Uploaded as supplementary information.
This policy applies to all data except where public deposition would breach compliance with the protocol approved by your research ethics board. If your data cannot be made publicly available for ethical or legal reasons (e.g., public availability would compromise patient privacy), please explain your reasons by return email and your exemption request will be escalated to the editor for approval. Your exemption request will be handled independently and will not hold up the peer review process, but will need to be resolved should your manuscript be accepted for publication. One of the Editorial team will then be in touch if there are any issues.

**We have uploaded the raw data as a supplemental file.**

3. We noticed that you used “data not shown”/"unpublished data" in the manuscript. We do not allow these references, as the PLOS data access policy requires that all data be either published with the manuscript or made available in a publicly accessible database. Please amend the supplementary material to include the referenced data or remove the references.

**We have now uploaded the report as a supplemental file.**

4. All figures and supporting information files will be published under the Creative Commons Attribution License ([creativecommons.org/licenses/by/4.0/](http://creativecommons.org/licenses/by/4.0/)). Authors retain ownership of the copyright for their article and are responsible for third-party content used in the article.

Figure 1: please (a) provide a direct link to the base layer of the map used and ensure this is also included in the figure legend; (b) provide a link to the terms of use / license information for the base layer. We cannot publish proprietary or copyrighted maps (e.g. Google Maps, Mapquest) and the terms of use for your map base layer must be compatible with our CC-BY 4.0 license.

**The map was created by the Partners in Health team. However, in light of the table/figure restrictions, and the request from reviewer one to expand on how comparable the population pre- and post-intervention, we have elected to remove the map and instead include a table on demographic population level data over time.**

5. Please provide separate figure files in .tif or .eps format. Kindly, removed them from the manuscript file.

**We have now uploaded the figures in separate files.**

6. We have noticed that you have uploaded Supporting Information files, but you have not included a list of legends. Please add a full list of legends for your Supporting Information files after the references list.

**We have now added a list of legends after the refences list. That list reads:**

**Legend Table 1: The table shows national population level trends in birth rate, incidence of HIV, proportion of the total population living in rural areas, proportion of the total population identifying as female, annual health expenditure per capita, and gross domestic product.**

**Legend Figure 1: Figure 1a shows the changes in quarterly average number of women attending prenatal care visits in four rural districts across Lesotho. Figure 1b shows the changes in quarterly average number of women presenting for their first and fourth antenatal care visits in four rural districts across Lesotho. Both figures show the change over time beginning in the third quarter of 2013 (before the National Health Reform) through the second quarter of 2017 (nearly three years into the National Health Reform).**

**Legend Figure 2: Figure two shows the percentage of births occurring within health centers in four rural districts across Lesotho, comparing pre- and post-National Health Reform interventions.**

**Legend Figure 3: Figure three shows the quarterly average number of children fully immunized at one year in each of four rural districts across Lesotho in which National Health Reform interventions took place, comparing pre- and post-intervention results.**

**Legend Figure 4: Figure four shows the trend in the number of HIV tests performed outside of the hospital across four rural districts in Lesotho over time, plotted against the targets for HIV testing established by the Universal Health Coverage goals. The figure shows the change over time beginning in the third quarter of 2013 (before the National Health Reform) through the fourth quarter of 2016 (more than two years into the National Health Reform).**

**Legend for Supplemental Table 1: The table above shows the three core areas identified as needs during the National Health Reform: Service Delivery, Health System Managerial Capacity, and the Professional Village Health Worker Program, as well as the specific areas for targeted for improvement within each area. Those areas became the focus of the interventions within the National Health Reform.**

**Legend for Supplemental Table 2: The table contains the raw, facility-level data, which was used to generate the results in the manuscript.**

**Legend for Supplemental File – Reference 24: This reference is the 2018 annual report on technical support authored by Partners in Health regarding the pharmacy and medical supply chain management detailing augmentation of cold chain capacity for five health centers to appropriately store several life-saving medicines.**

**Reviewer’s Comments:**

Reviewer #1: Thank you for this opportunity to provide a peer review for this interesting manuscript titled “Improving Access to Health Services through Health Reform in Lesotho: progress made towards achieving Universal Health Coverage.” The study basically set out to assess changes in quality metrics from Pre- to Post-National Health Reform during the period of 2013 to 2017 in the Kingdom of Lesotho. A baseline assessment was done in 70 facilities in four districts of Lesotho (making up catchment area of about 40% of the country’s population). The study observed improvements in Maternal health outcomes, HIV diagnostic capacity, and Childhood Immunization Rates. This study makes a case for encouraging and supporting further research into strategies utilized for implementation of multi-faceted health system strengthening interventions especially in developing countries. The fact that the study aims to assess the impact of Health Reforms in a developing country; an area where there is paucity of data is something to be encouraged. There are however areas that the Authors need to clarify in my opinion.

1. In the Methods Section, three areas were referred to as having deficits; namely supply-chain, district managerial capacity and Village Health Worker program. My observation is that there are no results to show if there was any difference in these areas after the National Health Reforms were introduced.

**Thank you for highlighting this point of confusion. In the methods section we identified the three domains you highlighted as key areas that we thought should be bolstered in order to improve the outcomes that were noted to be dramatically worse in Lesotho compared to other resource-limited settings. We thus considered the conducted interventions to bolster the supply chain, improve managerial capacity, and improve the Village Health Worker program and monitored the outcome of those intervention in terms of improvements in access to care for maternal health, improvements in childhood immunizations, and number of HIV tests conducted. We recognize the limitations in assuming causation based on that intervention, which we highlight in the limitations section.**

**We agree that it is important to make the above explicit, and have modified the methods section to now read: “Through the baseline auditing and consultation, PIH and the MOHSW identified three inter-related deficits, which, if improved might translate to improved access to and quality of care delivered.”**

**Further to make explicit what outcome measures we assessed, we included the following in the methods section: “The outcome measures assessed included: average quarterly number of women attending their first and fourth antenatal care visits, average quarterly number of women attending their post-natal care visits, average quarterly number of health centers adequately equipped to provide a facility-based birth, average quarterly number of facility-based births that occurred, average quarterly number of maternal emergency referrals placed and transported to the health facility, the availability of essential medications, the proportion of patients living with HIV and enrolled in care who were lost to follow-up, the average quarterly number of HIV tests performed at health facilities, and the average quarterly number of new HIV cases detected.”**

**Finally, we do report on the number of health facilities with improved health services delivery (inclusive of supply chain) when we report the following: “Importantly, the number of health centers adequately equipped to provide a facility-based delivery increased from 3% to 95% with an associated increase in deliveries occurring at a health facility from 2% to 33%” as well as: “Emergency referral and transportation of obstetric complications increased from 2.3 transports pre-intervention to 42 total transportations post-intervention, with similar increases noted across all districts.” And finally, “Additionally, by the end of the National Health Reform, the availability of essential medical supplies, tracer drugs, antiretroviral therapies, and vaccines to 90% or above.”**

2. The authors may wish to explain how they were able to arrive at the number of Health Facilities to evaluate, and how they came about the specific facilities and the Regions in Lesotho.

**Thank you for this suggestion. All facilities within the selected districts were included in the reform efforts. The districts were assigned by the Ministry of Health in Lesotho. We agree that making the above explicit will be important, and we have thus added the following to the introduction section: “Four districts were selected by the MOHSW to pilot the national health reform interventions: Berea, Leribe, Butha-Buthe and Mohale’s Hoek, which spanned an estimated catchment population of 815,520 individuals (40% of the country’s total population). All health facilities in those districts were included.”**

3. The authors may wish to mention measures taken to ensure comparability between the results of the two assessments (Pre and Post National Health Reform).

**Thank you for this suggestion. Unfortunately, no census data are available for the rural districts in Lesotho, thus we are unable to compare the demographics of the district in order to ensure the populations are comparable. We have done two things to address this limitation.**

**First, we have noted it explicitly as a limitation in the manuscript, stating: “Finally, one challenge we encountered was a paucity of district level census demographic data, making determination of the rate of change not possible. Such a challenge is likely to be evident in other similarly resource limited settings. Future efforts should strive for developing improved infrastructure for both population and disease surveillance, which may facilitate a more accurate understanding of population level rates of disease, as well as provide the foundation for public health responses to outbreaks.”**

**Second, we have included a table of population level demographics over time (from 2011 – 2019) in order to demonstrate overall trends in demographics, noting: “We further utilized data from the World Bank (39) to evaluate population level demographics over time, in order to assess for potential confounding by differences in the population before and after the National Health Reform (Table 1).”**

**Overall, the population level demographics do not appear to have changed substantially, except for a subtle decrease in birth rate over time and a subtle decrease in the incidence of HIV over time at the national population level. Both of those factors we would postulate would, if anything result in slight decreases in healthcare utilization, whereas our manuscript noted an increase in utilization, thus we do not suspect (to the extent that we are able to determine) that the demographics within the rural districts changed substantially over time. We have added the following reflections to the discussion section: “Likewise, changes in demographics and risk-factors at the district level over time may have also confounded our results; however, in the absence of district-level data, the population level trends in demographics (Table 1) did not suggest significant difference before and after the interventions.”**

4. The conclusion by the authors that there was “improvement in health outcomes among Maternal and Child health, as well as HIV testing and availability of essential health commodities” may be difficult to justify, as essentially what the study reported was an increase in utilization rates.

**We agree that based on our study design we cannot conclude that we improved health outcomes as a consequence of utilization metrics. We have modified the conclusion to now read: “We observed increases in maternal antenatal and post-natal care visits as well as emergency referrals, increases in child immunization rates, and improvements in retention in care among patients living with HIV as well as increased HIV testing. We also noted increased availability of essential health commodities. Although surrogates for concrete outcomes, those correlations are encouraging and support further research into the strategies employed with consideration of implementation of similarly multi-pronged health system strengthening interventions in other settings.”**

**We have also modified the conclusion in the abstract to now read: “We observed increases in maternal antenatal and post-natal care visits, child immunization rates, and HIV testing during a period of national health reformation in the Kingdom of Lesotho. We further noted increased availability of essential health commodities. While surrogates for concrete health outcomes, those correlations are encouraging and support further research into the strategies employed with consideration of implementation of similarly multi-pronged health system strengthening interventions in other settings.”**

Reviewer #2: Provide study data
1. What was the study design? Was this a quasi-experimental study? (A controlled or uncontrolled before-and-after study)? If so, please state the duration of the reform period and at what point the endline assessment was done.

**Thank you for these important questions. The study design was a one arm pre- and post-intervention time series analysis. We have added the following to the methods section: “We then conducted a one arm pre- and post-intervention time series analysis, evaluating changes in the outcomes above after three years of operationalizing the National Health Reform compared to before (2013). Data were collected in 2017, thus that was considered the end point for the study, however the activities of the National Health Reform are ongoing.”**

2. Study results in abstract are reported as absolute numbers yet project aimed to improve metrics that are reported in rates (to cater for population size changes). Please revise e.g. instead of reporting on number of new ANC attendances, you could report on proportion of expected new ANC attendance per number of estimated pregnancies; or for immunisation, immunisation coverage instead of absolute numbers.

**Thank you for this suggestion. We agree that it would be ideal to report on rates rather than absolute numbers. Unfortunately, one of the challenges faced in Lesotho is the absence of census data on overall proportions of such events (e.g., the number of pregnant women in the districts, number of children eligible for immunizations, number of people eligible for HIV testing, etc. are not known). Therefore, we do not have a denominator with which to calculate a rate.**

**We have now made the above explicit in the future section, stating: “Finally, one challenge we encountered was a paucity of district level census demographic data, making determination of the rate of change not possible. Such a challenge is likely to be evident in other similarly resource limited settings. Future efforts should strive for developing improved infrastructure for both population and disease surveillance, which may facilitate a more accurate understanding of population level rates of disease, as well as provide the foundation for public health responses to outbreaks.”**
