# Supplementary material for: Improving access to health services through health reform in Lesotho: Progress made towards achieving Universal Health Coverage
Source: PLOS Glob Public Health. 2022 Nov 16;2(11):e0000985. doi: 10.1371/journal.pgph.0000985 (PMC10021396; doi:10.1371/journal.pgph.0000985)
Supplement: Attachment — Submitted filename: Allan-Blitz - Response Letter - 9.28.22.docx [file pgph.0000985.s008.docx]

Dear editor of *PLOS Global Public Health,*

Thank you very much for the thoughtful comments on the paper “*Improving Access to Health Services Through Health Reform in Lesotho: Progress Made Towards Achieving Universal Health Coverage.*” We have reviewed and responded to the comments from the editor, and have reformatted the manuscript accordingly. Below are our responses to each comment. Please do not hesitate to contact us if anything further is needed. We hope that this revised manuscript will be accepted for publication.

Sincerely,

Lao-Tzu Allan-Blitz

**Corresponding Author Contact Information:**

Dr. Lao-Tzu Allan-Blitz

Department of Medicine, Brigham and Women’s Hospital

75 Francis Street

Boston, MA, 02115, USA

Email: lallan-blitz@partners.org, phone: (805) 896-5313

Editor Comments

1. Please refer to a reporting standard, such as [STROBE](https://www.equator-network.org/reporting-guidelines/strobe/) or [TREND](https://www.equator-network.org/reporting-guidelines/improving-the-reporting-quality-of-nonrandomized-evaluations-of-behavioral-and-public-health-interventions-the-trend-statement/). Include the completed reporting standard checklist with your revised submission.

**We have now included a completed STROBE checklist.**

2. Pay particular attention to the reporting elements needed in the Methods section. You have provided a very long description of the development and content of the intended intervention - this can be heavily cut (with details in supplement). You have provided almost no information on the outcomes, data collection, or analysis. For example, what were the primary/secondary outcomes, why did you select these (and not others). You say you did 'time series' analysis, but provide no details - and the results do not present any time series analysis results. Please address each of the essential reporting elements in full.

**Thank you for highlighting these deficiencies. We agree that much more precision in reporting the methods used for data collection, outcome definition, and data analysis were needed. We have re-written the methods section, moving much of the text to an appendix as suggested. We have further specifically defined the primary and secondary outcome measures, as well as provided justification for those selections.**

**We have included the following detail data collection methods:**

**“Baseline data collection was done in all the health facilities in the four districts from July 2013 to June 2014 via standardized collection tools. Those tools included standardized interviews with key stakeholders, as well as population-level assessment of key facility health metrics that were used as our outcomes of interest as described in detail below (Appendix 1). Those data were collected by a monitoring and evaluation team from the PIH and MOHSW who went to all facilities and collected data from the facility registers over a period of six months. The collection of the data was done on password protected and encrypted electronic tablets.”**

**The following are the changes made to highlight the outcomes of interest:**

**“The primary outcomes of interest were defined in three domains: 1) utilization of healthcare services, 2) children receiving immunizations at the facility, and 3) HIV testing and retention in care. Those domains were selected as they were most readily accessible for analysis, and most likely directly impacted by the specific interventions (Appendix 2). With regards to maternal health, the outcome measures were the change pre- and post-intervention in average quarterly number of women attending their first and fourth antenatal care visits, average quarterly number of women attending their post-natal care visits, average quarterly number of health centers adequately equipped to provide a facility-based birth, average quarterly number of facility-based births that occurred. With regards to childhood immunizations, we evaluated the change in the average quarterly number of children who were immunized at one year of life at the clinic. Complete immunization at one year of life was defined as having received BCG, Hepatitis B, Polio, Diphtheria-Tetanus-Pertussis, *Haemophilus influenzae* type B, Pneumococcal (conjugate), Rotavirus, Measles-Mumps-Rubella, and Human Papilloma Virus (22). Finally, with regards to HIV testing and retention in care, our primary outcomes of interest were the proportion of patients living with HIV and enrolled in care who were lost to follow-up, the average quarterly number of HIV tests performed at health facilities, and the average quarterly number of new HIV cases detected.”**

**“Our secondary outcomes of interest were average quarterly number of maternal emergency referrals placed and transported to the health facility and the availability of essential medications. Those two metrics were selected as there was no emergency referral system prior to the national health reform, and the availability of essential medications was subject to numerous confounding variables that we were unable to control for, thus it was selected as a secondary outcome."**

**We do wish to clarify, however, the statistical approach used. A one arm pre- and post- time series analysis is the evaluation of the change in an outcome at one time point compared to another. That is the analysis that was performed and reported. There are other, more complex, time series analyses, however the data did not lend themselves to those procedures, unfortunately.**

3. The Introduction can be heavily cut. Please include a clear Objective and/or Hypothesis that you seek to test.

**Thank you for this suggestion. We have edited down the introduction section, and have now included the specific objective as follows: “The present report aims to evaluate the impact of the numerous interventions of the national health reform on various outcome metrics.”**

4. Please include the study protocol as a supplemental file, and data analysis plan if available. And make clear in the reporting where you deviated from the pre-specified protocol (including how the intervention evolved over time, and differences in the data you collected, how you analysed it and reported it). Most health systems interventions will not have been delivered (or evaluated) exactly as planned - and this is important information to disclose.

**Thank you for this suggestion. We very much agree that including more detailed information on the protocol, deviations from the protocol, and differences in the analysis are important for understanding the results of this type of study. We have now included the protocol as an appendix, which includes report of the plan for the analysis, however there was not a separate analysis plan. We have further included the following details on how the study was implemented and how that implementation deviated from the protocol:**

**“Over the period of the intervention, there were several deviations from the protocol (Appendix 3) both from an implementation standpoint as well as from an analysis standpoint. First, in addition to the four pilot villages, the health reform interventions were initially planned for implementation in the central ministry of health. Frequent internal turnover of government staff precluded that implementation. From an analysis standpoint, we were unable to collect the population level metrics on tuberculosis outcomes, thus those outcomes were excluded from the present report. Similarly, the data collected regarding initiation of antiretroviral therapy among patients diagnosed with HIV contained numerous missing entries, thus was excluded from the analysis to avoid introducing bias.”**

5. Your authorship list is overwhelmingly US-centric. You cite involvement of the MOHSW in planning, implementation, and data collection. I find it concerning that you did not involve MOHSW in authorship, both from a research validity perspective (they will understand things you do not) and from a research impact perspective (they will be prime users of this data). Please provide explanation in your Reflexivity statement about why you included only a single author from Lesotho and no government partners.

**Thank you for this comment. We entirely agree with the importance of including authors from the local context for both research validity and research impact. Since Partners in Health is an international organization, we had included the affiliation for all local authors under one single Partners in Health, but we agree that separating them out will be meaningful. To note explicitly, 10 of the study authors (including one of the co-first authors) are Basotho and other African nationals who live and work in Lesotho and were a part of the study implementation. That has now been made explicit.**

**We further recognize the importance of direct collaboration with the Lesotho government. During the work itself, countless government health workers and high-level officials were directly involved on a day-to-day basis. Indeed, this work is the result of our formal public-private partnership between Partners In Health in Lesotho and the Lesotho Ministry of Health and Social Welfare. Unfortunately, given the time that has elapsed since project implementation, and the restrictions around government staff authoring articles, we were not able to identify any of our government colleagues who were willing/able to be formally listed as an author.**

Journal Requirements:

1. "Allan-Blitz - Figure 1 - 5.25.22.tiff", "Allan-Blitz - Figure 2 - 5.25.22.tiff", "Allan-Blitz - Figure 3 - 5.25.22.tiff", and "Allan-Blitz - Figure 4 - 5.25.22.tiff" files are over our file size limit of 10MB. Please reduce the file size to no more than 10MB. For further help on compressing figures visit: <https://journals.plos.org/globalpublichealth/s/figures>

**We have now compressed all figures to be less than 10 MB.**

2. Please include your main table (Table 1) as part of your main manuscript and remove the individual file. Please note that supplementary tables should remain as separate "Supporting Information" files.

**We have now included Table 1 in the main document and removed the individual file.**

3. We noticed that you used “unpublished”/“unpublished data” in the manuscript. We do not allow these references, as the PLOS data access policy requires that all data be either published with the manuscript or made available in a publicly accessible database. Please amend the supplementary material to include the referenced data or remove the references.

**Thank you for this clarification. We have removed those references and now refer to them in the manuscript as supplemental document, which has been uploaded.**

4. Please update your online Competing Interests statement. If you have no competing interests to declare, please state: “The authors have declared that no competing interests exist.”

**We have updated the online competing interests statement to read: The authors have declared that no competing interests exist.”**

5. Please amend your online detailed Financial Disclosure statement. This is published with the article. It must therefore be completed in full sentences and contain the exact wording you wish to be published.

a. State the initials, alongside each funding source, of each author to receive each grant.
b. State what role the funders took in the study. If the funders had no role in your study, please state: “The funders had no role in study design, data collection and analysis, decision to publish, or preparation of the manuscript.”

**We have now amended the financial disclosures statement to read: “Funding for this study came from the Skoll Foundation (grant number 19-44478) (JM) and the Wagner Foundation (project code AVA0436) (JM). The funders had no role in study design, data collection and analysis, decision to publish, or preparation of the manuscript.”**
